# Determination of enantiomeric excess of carboxylates by fluorescent macrocyclic sensors†
†Electronic supplementary information (ESI) available: Synthesis and characterization of **S1–S4**, fluorescence spectra, experimental detail of microarray, and results of multivariate analysis and DFT calculations. CCDC 1435022. See DOI: 10.1039/c5sc04235f


**DOI:** 10.1039/c5sc04235f

**Published:** 2015-12-14

**Authors:** Ali Akdeniz, Tsuyoshi Minami, Sagiri Watanabe, Maki Yokoyama, Tadashi Ema, Pavel Anzenbacher

**Affiliations:** a Department of Chemistry and Center for Photochemical Sciences , Bowling Green State University , Bowling Green , Ohio 43403 , USA . Email: pavel@bgsu.edu; b Division of Applied Chemistry , Graduate School of Natural Science and Technology , Okayama University , Tsushima , Okayama 700-8530 , Japan . Email: ema@cc.okayama-u.ac.jp

## Abstract

Chiral fluorescent chemosensors featuring macrocycles comprising BINOL auxiliary and an array of hydrogen bond donors were synthesized. To enhance fluorescence of the chemosensors, conjugated moieties were attached to the 3,3′-positions of the BINOL auxiliary. The resulting chemosensors recognize a number of carboxylates, namely, enantiomers of ibuprofen, ketoprofen, 2-phenylpropanoate, mandelate, and phenylalanine in a stereoselective fashion. Depending on the structure of the chemosensor, the presence of carboxylate yields fluorescence quenching or amplification. This information-rich signal can be used to determine the identity of the analyte including the sense of chirality. Quantitative experiments were performed aimed at analysis of enantiomeric excess of chiral carboxylates. The quantitative analysis of enantiomeric composition of ibuprofen, ketoprofen, and phenylalanine shows that the sensors correctly identify mixtures with varying enantiomeric excess and correctly predict the enantiomeric excess of unknown samples with error of prediction <1.6%.

## Abstract 2

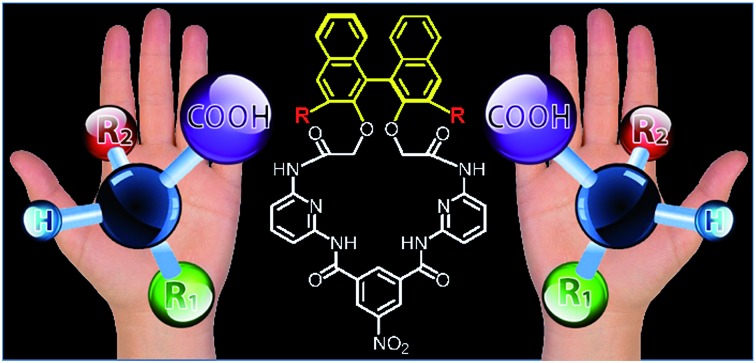
Chiral fluorescent macrocycles enable detection of carboxylate enantiomers using naked eye, which allows for quantitative measurement of the enantiomeric excess with high accuracy (error < 1.6%).

## Introduction

The carboxylate anions play important roles in a number of natural processes and therefore have a tremendous impact on biotechnology and the pharmaceutical industry.^1^,^2^ This is particularly true for chiral carboxylates, which have become a central focus for applications in asymmetric synthesis, chiral catalysis, and drug development. Thus, the volume of studies devoted to quantification of enantiomeric excess (ee) of chiral compounds has rapidly increased.^3^ Despite all of these efforts, methods for the determination of the enantiomeric composition are labor intensive, require expensive instrumentation such as chiral HPLC,^4^ circular dichroism^5^ or may involve derivatization or use of chiral solvating agents (NMR),^6^ and chromatographic purification of the product.^4^ Recently, chiral optical sensors have attracted significant attention due to their easy implementation and potential application in high-throughput assays.^7^ In this regard, a number of examples of ee determination of analytes containing carboxylic acids, amines, alcohols and ketones have been demonstrated.^8^

**Fig. 1:**
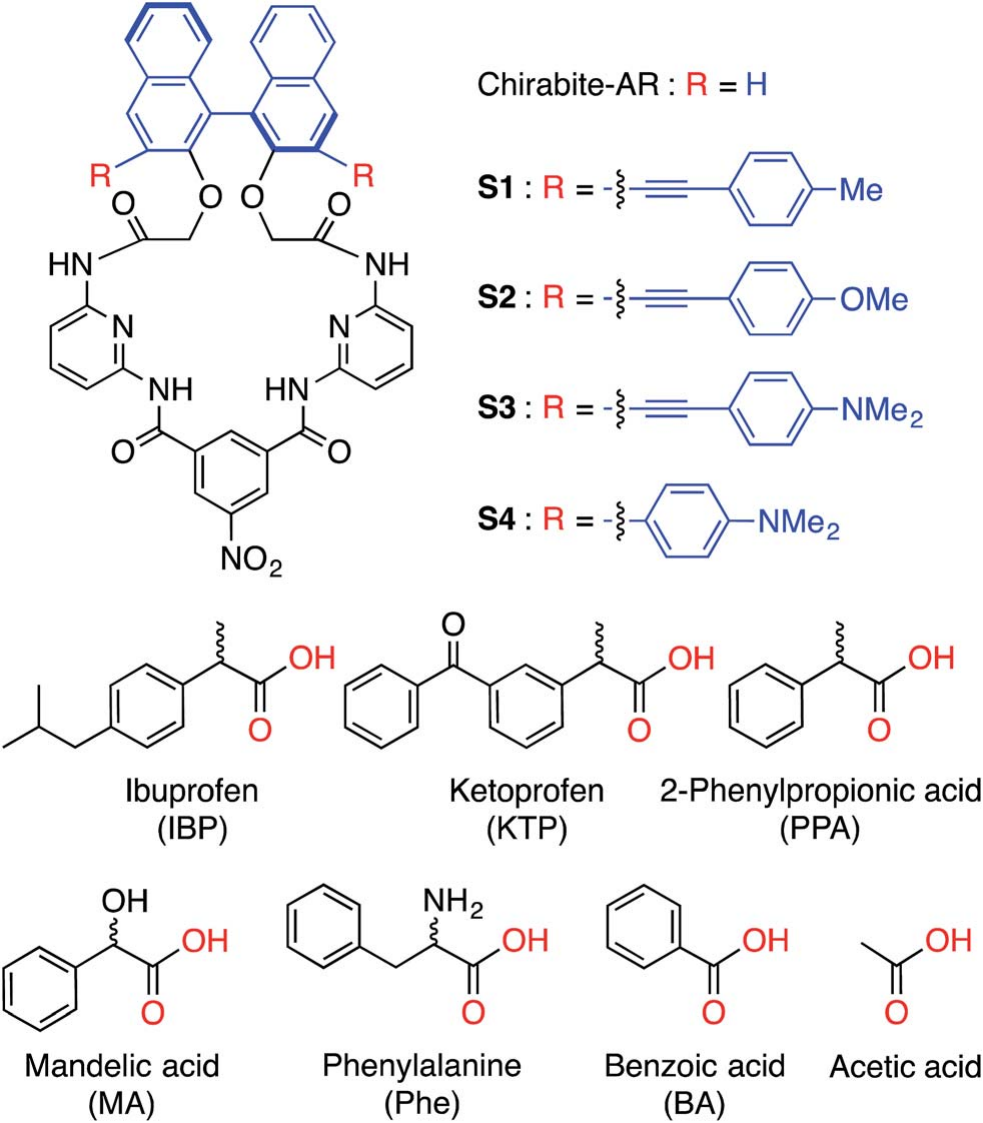
Structures of hosts (**S1–S4**) (top) and guests (bottom) used in this study. Corresponding anions were used as tetrabutylammonium salts.

In the last decade, a number of BINOL-based optical sensors have been reported.^9^ Recently, we have demonstrated on chirabite-AR (Fig. 1), a macrocyclic ligand that features chiral naphthalene auxiliary^10^ and hydrogen bonding donors to achieve formation of the complex with carboxylic acids.^11^ The intrinsic chirality of the macrocycle impacts the stability of the complexes with chiral carboxylates while tuning the size and shape of the macrocyclic cavity can improve overall recognition ability and enantioselectivity.^12^,^13^ The previous results suggested that increasing the ability of the receptors to measure ee of the chiral carboxylates in a quantitative manner requires dramatic changes in the design of the sensors (Fig. 1). Firstly, we decided to investigate the incorporation of conjugated substituents to the 3,3′-positions of the binaphthalene moiety of chirabite-AR to achieve improved chiral induction by limiting access to the chiral cavity of the macrocycle. Secondly, we designed the fluorophores in a way that some sensors display a fluorescence quenching in the presence of the analyte while the others display an increase in the fluorescence.

Ibuprofen and ketoprofen, non-steroidal anti-inflammatory drugs (NSAID), are extensively used in human and veterinary medicine. NSAIDs inhibit cyclooxygenase enzymes and consequently prostaglandin biosynthesis.^14^ Even though the inhibition ability of the enantiomers of these drugs are significantly different, ibuprofen and ketoprofen are marketed as racemates. For example, the (*S*)-enantiomer of ibuprofen shows 160 fold inhibition ability in prostaglandin syntheses *in vitro* comparing the (*R*)-enantiomer.^15^ For similar reasons the Federal Drug Administration (FDA) started requiring manufacturers to provide drugs in enantiomerically pure form.

Here we report on new fluorescent chemosensors **S1–S4** capable of discriminating carboxylates (Fig. 1). The four probe array (**S1–S4**) and in a special case even a single chemosensor (**S4**) achieved 100% correct classification of the analytes and precise determination of enantiomeric compositions, demonstrating their excellent chiroptical abilities.

## Results and discussion

**Scheme 1:**
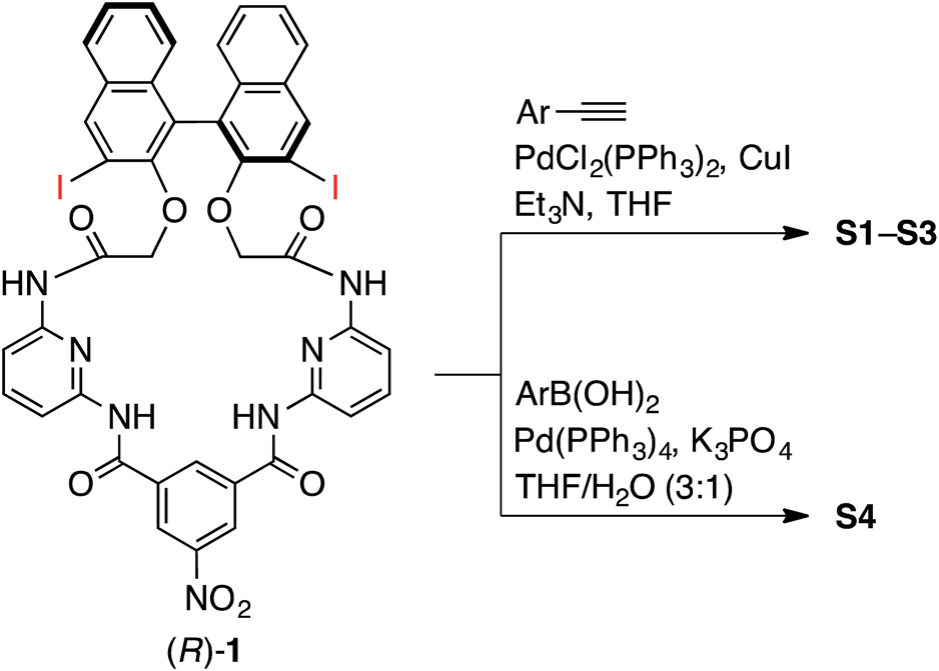
Synthesis of chemosensors **S1–S4**.

The synthesis of macrocycles **S1–S4** is shown in Scheme 1. This synthetic method is characterized by rapid access to a variety of derivatives from a single precursor, (*R*)-**1**, *via* cross-coupling reactions (for the synthesis of (*R*)-**1**, see ESI†). Indeed, the Sonogashira cross-coupling reactions of (*R*)-**1** with aryl acetylenes gave macrocycles with arylethynyl substituents at the 3,3′-positions of the binaphthyl moiety, while the Suzuki–Miyaura cross-coupling reactions of (*R*)-**1** with aryl boronic acids gave macrocycles with aryl substituents at the 3,3′-positions of the binaphthyl moiety. Among them, fluorescent compounds **S1–S4** were selected and used for the recognition of carboxylates.

**Fig. 2:**
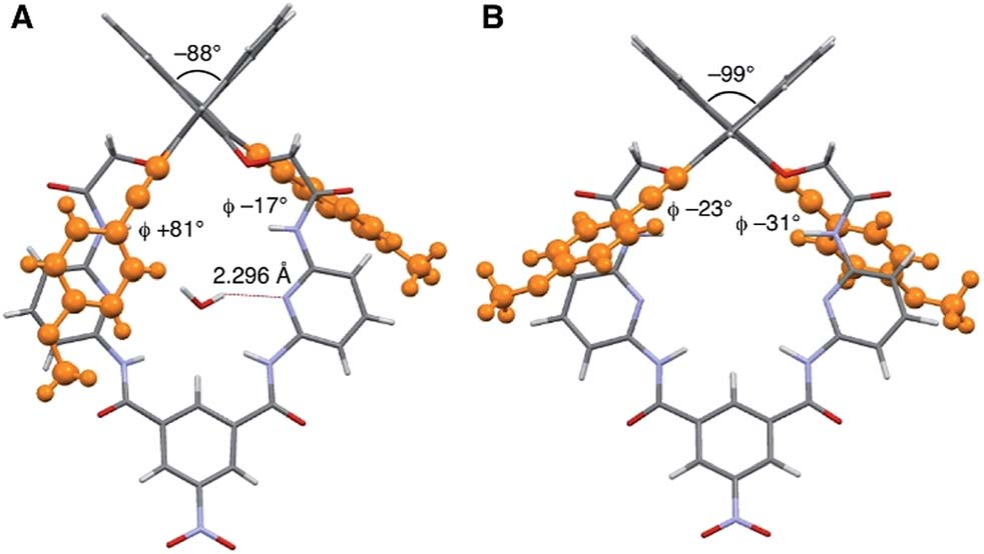
Structures of macrocycle **S2**, where the 3,3′-substituents are highlighted by orange ball-and-stick representation. (A) X-ray crystal structure. A water molecule in the binding cavity is shown, while a chloroform molecule located outside the cavity is omitted for clarity. (B) DFT-optimized structure at the B3LYP/6-31G* level. Ø denotes the dihedral angle between the 4-methoxyphenyl group and the naphthyl group.

We explored the structures of the macrocycles by X-ray crystallography, DFT calculations, and NMR spectroscopy. Fig. 2A shows a crystal structure of macrocycle **S2**. The binding cavity is well restricted by the arylethynyl substituents at the 3,3′-positions of the binaphthyl moiety orthogonal to the lower segment of the macrocycle. The fact that the arylethynyl moiety, which constitutes the fluorophore in the chemosensor, is close to the binding cavity is significant because fluorescence response can be sensitive to analyte binding. In the crystalline state, the two 4-methoxyphenyl groups adopt different conformations; the right one interacts with an adjacent **S2** molecule *via* π–π stacking (not shown) and is conjugated with the naphthalene ring, whereas the left one is parallel to the lower segment and almost orthogonal to the naphthalene ring connected by a triple bond. Fig. 2B shows a structure of **S2** optimized by DFT calculations. The two 4-methoxyphenyl groups adopt similar conformations, and the dihedral angle of the binaphthyl moiety (–99°) of **S2** is close to that (–101°) of chirabite-AR (X-ray crystal structure).^13^^1^H and ^13^C NMR spectra indicated that the conjugated moieties (arylethynyl or aryl substituents) of **S1–S4** rapidly rotate in the solution at room temperature (ESI†).

**Fig. 3:**
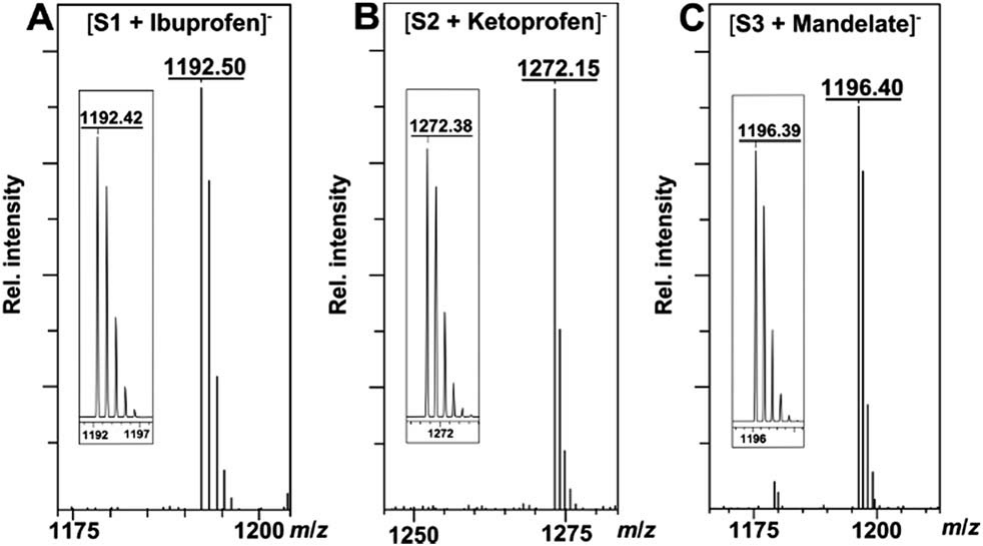
(A) ESI mass spectrum of the complex of **S1** and ibuprofen. Inset: calculated isotope pattern for C_73_H_58_N_7_O_10_^–^. (B) ESI mass spectrum of the complex of **S2** and ketoprofen. Inset: calculated isotope pattern for C_76_H_54_N_7_O_13_^–^. (C) ESI mass spectrum of the complex of **S3** and mandelate. Inset: calculated isotope pattern for C_70_H_54_N_9_O_11_^–^.

**Fig. 4:**
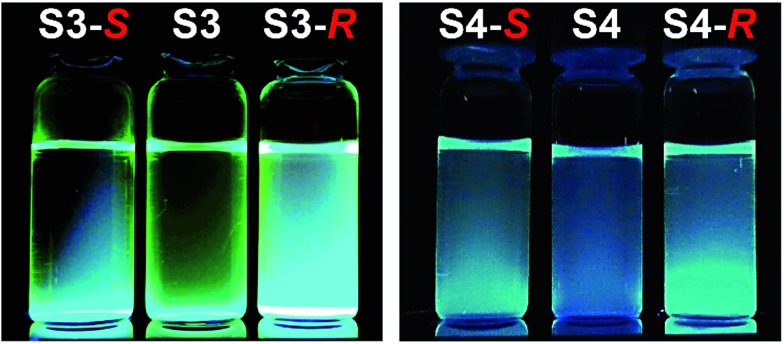
Examples of naked-eye detection of differential recognition of enantiomers of ibuprofen. Left panel: sensor **S3** + (*S*)-ibuprofen, **S3** alone, **S3** + (*R*)-ibuprofen. Right panel: sensor **S4** + (*S*)-ibuprofen, **S4** alone, **S4** + (*R*)-ibuprofen. Chemosensors **S3** and **S4** were excited by black light (365 nm).

The binding ability and stoichiometry of chemosensors to selected carboxylates were tested using electrospray ionization (ESI) mass spectrometry. ESI MS spectra revealed the formation of strong complexes between the macrocycles and guests with 1 : 1 stoichiometry (Fig. 3). The preliminary simple vial experiments (Fig. 4) show that for example, chemosensors **S3** and **S4** display different responses to the presence of (*R*)- or (*S*)-enantiomers of ibuprofen. In both photographs the solution of the sensor alone is shown in the center. Here, (*R*)-ibuprofen increases the fluorescence intensity of **S3** and **S4** significantly more than the (*S*)-enantiomer.

The design of the fluorophores followed a simple design considerations: from the previous unpublished work we realized that **S1** and **S2** show fluorescence quenching, presumably due to photoinduced electron transfer (PET) upon addition of incremental amounts of analyte. In contrast, electron-rich fluorophores (**S3** and **S4**) displayed red-shifted emission and a low quantum yield (<10%). This is in agreement with the energy gap law.^16^ In the presence of bound anion the fluorescence increases. The latter is most likely due to the formation of a rigid complex that prevents the dissipation of the excited state energy *via* vibrational and rotational modes. Thus, to increase the information density in the response of the sensor array we decided to include two sensors that display fluorescence quenching (**S1** and **S2**) and two sensors that show fluorescence amplifications (**S3** and **S4**). The different responses of the chemosensors upon addition of enantiomers of the same compounds can also reveal the enantiomeric composition of the samples even in analytes that otherwise display similar association constants (*K*_assoc_). To quantify the binding affinity of the analytes, values of *K*_assoc_ were calculated and are listed in Table 1. The values of enantiomeric fluorescence difference ratio (ef) are reported in the ESI.†

**Table 1:** Association constants (*K*_assoc_, M^–1^) determined by fluorescence titrations^*a*^

Guest	**S1**	**S2**	**S3**	**S4**
(*R*)-IBP	6.7 × 10^4^	1.6 × 10^4^	3.0 × 10^4^	3.4 × 10^5^
(*S*)-IBP	4.8 × 10^4^	6.2 × 10^4^	3.1 × 10^4^	5.1 × 10^6^
(*R*)-KTP	1.4 × 10^5^	8.2 × 10^4^	5.7 × 10^4^	ND^*b*^
(*S*)-KTP	6.2 × 10^4^	6.2 × 10^4^	4.1 × 10^4^	6.3 × 10^4^
(*R*)-PPA	3.1 × 10^4^	2.6 × 10^4^	3.9 × 10^4^	4.0 × 10^6^
(*S*)-PPA	4.5 × 10^4^	2.7 × 10^4^	8.0 × 10^5^	2.0 × 10^5^
(*R*)-MA	8.4 × 10^3^	1.9 × 10^4^	2.9 × 10^3^	6.8 × 10^4^
(*S*)-MA	1.4 × 10^4^	2.7 × 10^4^	3.0 × 10^3^	5.7 × 10^4^
(*R*)-Phe	6.8 × 10^4^	4.6 × 10^4^	2.7 × 10^5^	3.8 × 10^5^
(*S*)-Phe	7.0 × 10^4^	4.3 × 10^4^	1.6 × 10^5^	3.9 × 10^5^
BA	2.0 × 10^4^	1.2 × 10^5^	4.0 × 10^4^	1.1 × 10^5^
Acetate	2.5 × 10^4^	3.3 × 10^4^	3.5 × 10^4^	1.0 × 10^5^

^*a*^Fluorescence titrations were performed in propionitrile at 22 °C. All guests were added as tetrabutylammonium salts. Association constants were calculated by the nonlinear least-squares method.^17^ The errors of the curve fitting <15%. For details, see ESI.

^*b*^Association constant could not be calculated due to small changes in fluorescence response.

**Fig. 5:**
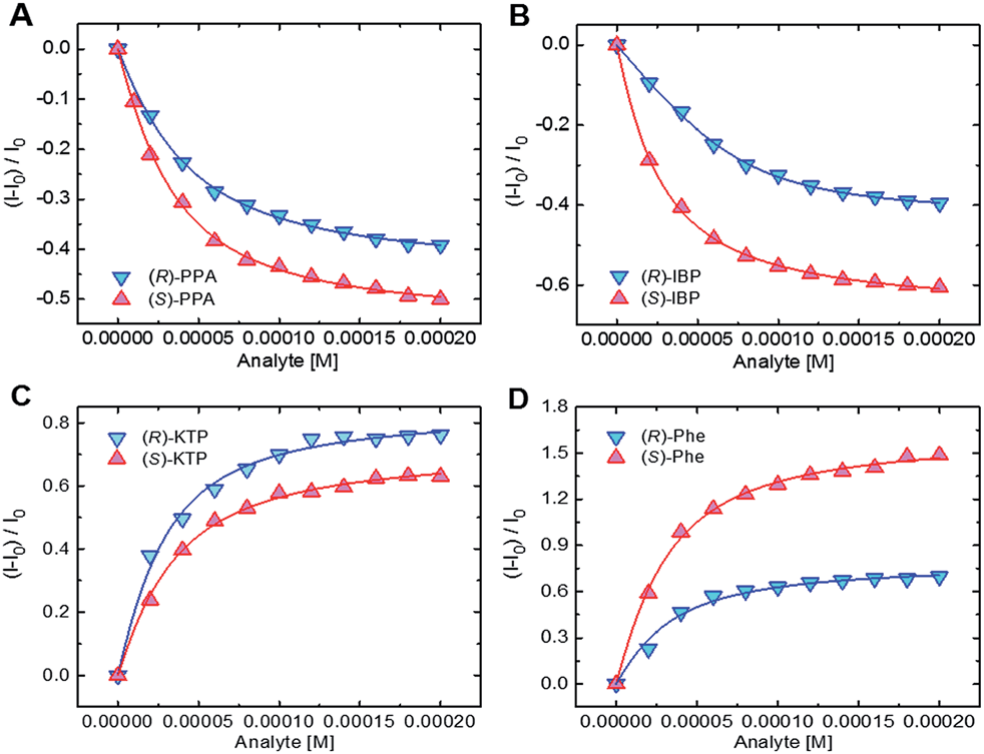
Binding isotherms for the complexation of (A) **S1** with PPA, (B) **S2** with IBP, (C) **S3** with KTP, and (D) **S4** with Phe. [**S1–S3**] = 20 μM, [**S4**] = 40 μM.

To obtain an insight into the recognition process and quantify the chiral discrimination of the analyte enantiomers, we performed a series of titration experiments for each chemosensor using the carboxylates, specifically, enantiomers of ibuprofen (IBP), ketoprofen (KTP), 2-phenylpropanoate (PPA), mandelate (MA), and phenylalanine (Phe) (Fig. 1). Acetate and benzoate (BA) were also included for reference purposes and as potential impurities. For details on the titration experiments see the ESI.† In the titration experiments, fluorescence spectra were recorded after addition of incremental amount of carboxylate guests. Binding isotherms were obtained from fluorescence changes as a function of guest concentrations (Fig. 5).

As one can see, in most cases the values of the binding constants are slightly different for enantiomers. The differences in binding constants and fluorescence responses are specific for each analyte. Furthermore, the magnitudes of binding constants also show that the chemosensors are cross-reactive as one chemosensor can bind several guests, albeit in most cases with different affinities. Finally, the combination of the binding affinities and the fluorescence responses yields a combination of data unique for each compound. Because the chemosensors are chiral, the individual enantiomers of the same compounds are distinguished from each other. This is an important feature as it suggests that discrimination among multiple chiral analytes may be possible in an array-based assay.

To test this hypothesis, mixtures of chemosensors and chiral carboxylates were dissolved in propionitrile and pipetted into conventional 384 well plates. Fluorescence intensities at 370 nm, 390 nm, 410 nm, 430 nm for **S1** and **S2**, and 480 nm, 490 nm, 500 nm, 520 nm for **S3** and **S4** were recorded using a standard plate reader (for details see ESI†). The fluorescence outputs were analyzed using linear discriminant analysis (LDA).^18^ LDA is a frequently used supervised pattern recognition method for reduction of dimensionality and classification of the multivariate data. LDA models the similarity by maximizing the distance between the classes and minimizing the distance between the trials within the clusters. Cross-validation procedure, consisting of a model development and model testing, is performed to ascertain the level of correct classification of the observations within the clusters.

**Fig. 6:**
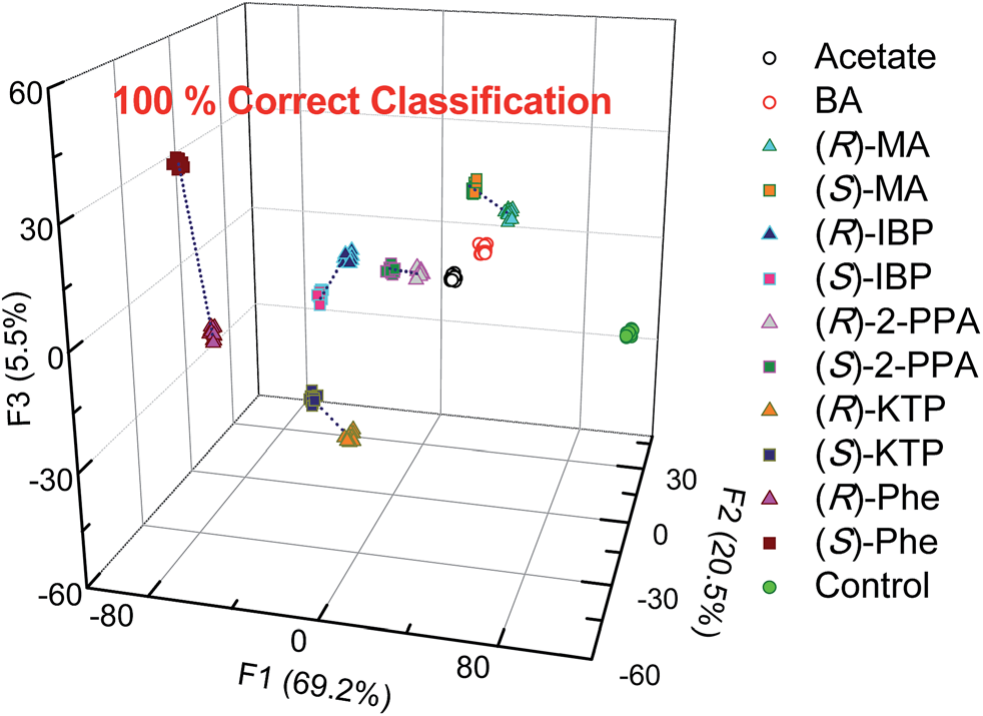
Linear discriminant analysis (LDA) of twelve carboxylates show 100% correct classification by employing the **S1–S4** array. [**S1–S3**] = 20 μM, [**S4**] = 40 μM, [analyte] = 100 μM.

Thus, LDA was performed to investigate analyte clustering and classification. Fig. 6 shows response space defined by the first three canonical factors (F1–F3). Excellent recognition capability of the probes is reflected by the 100% correct classification of twelve guests and control (Fig. 6). Importantly, enantiomers of the same chiral compounds are resolved. Here, the distance between two enantiomers of the same compound reflects the difference in the spectral behavior of chemosensors and analyte enantiomers.

**Fig. 7:**
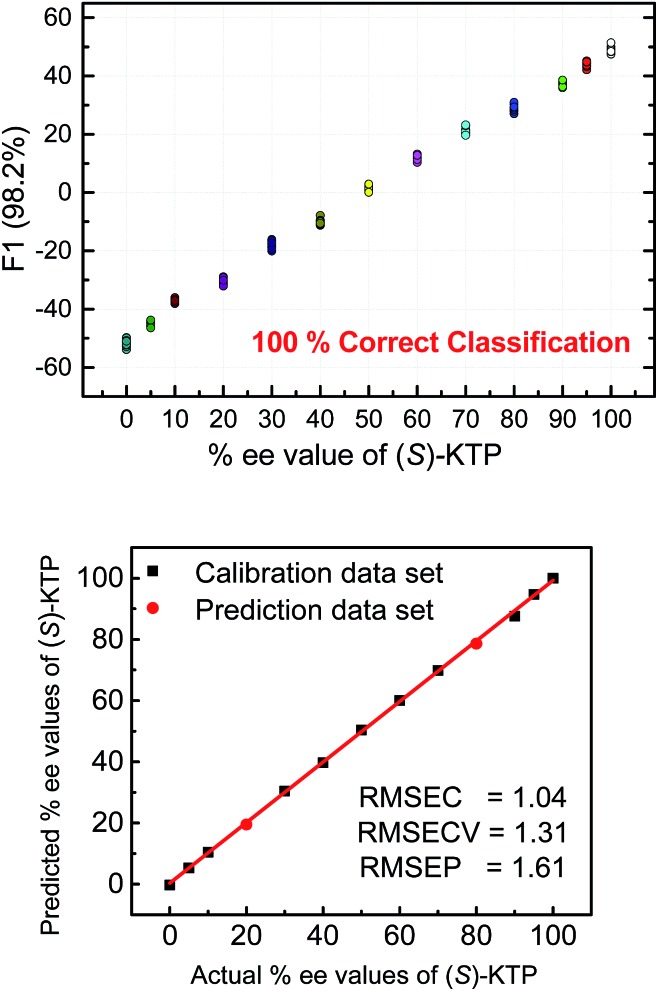
Top: LDA corresponding to semiquantitative assay of enantiomeric composition of KTP. Bottom: quantitative analysis of enantiomeric composition of KTP by using SVM. The analysis was achieved by the **S1–S4** array. [**S1**] = [**S2**] = [**S3**] = 20 μM, [**S4**] = 40 μM, [(*R*) + (*S*) analyte] = 100 μM.

**Fig. 8:**
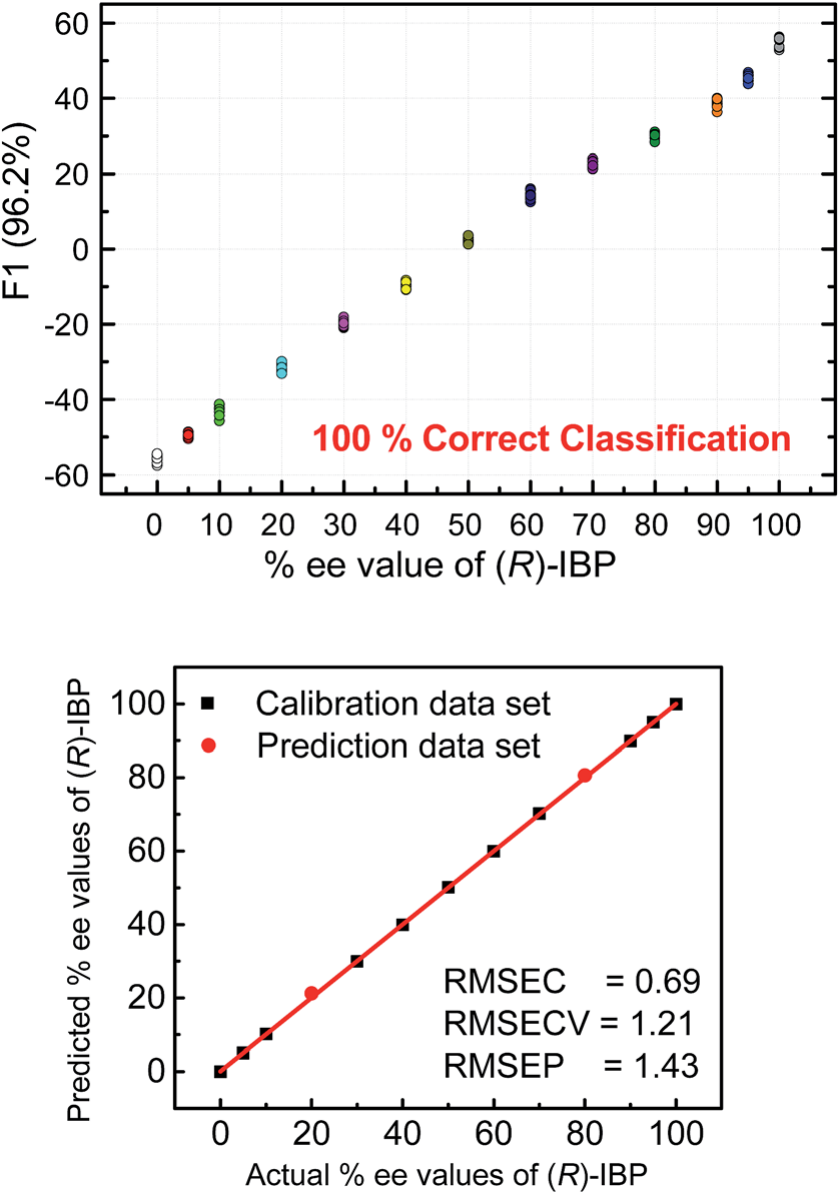
Top: LDA corresponding to semiquantitative assay of enantiomeric composition of IBP. Bottom: quantitative analysis of enantiomeric composition of IBP by using SVM. The analysis was achieved by the **S1–S4** array. [**S1**] = [**S2**] = [**S3**] = 20 μM, [**S4**] = 40 μM, [(*R*) + (*S*) analyte] = 100 μM.

The next step following the successful qualitative analysis was to elucidate the enantiomeric purity of analytes in a semiquantitative fashion. Thus, we performed semiquantitative analysis of enantiomeric composition of ketoprofen (Fig. 7 top) and ibuprofen (Fig. 8 top) samples. LDA of the enantiomeric compositions of analytes reflects the dependence of fluorescence response changes on the enantiomeric compositions. The results show 100% correct classification of all enantiomeric compositions with a linear trend in the position of the clusters corresponding to their ee values.

The linear trend in the evolution of the data in the semiquantitative analysis encouraged us to perform regression analysis to determine the enantiomeric compositions of unknown samples. Using 13 data points for calibration and 2 data points as unknown, we performed regression analysis utilizing support vector machine (SVM) algorithm (Fig. 7 and 8 bottom).^19^ Briefly, SVM is a supervised classification method that seeks to separate classes by mapping the input into an n-dimensional vector space using kernel functions. The data points are linearly separated in the n-dimensional feature space. The SVM regression method constructs calibration models serving to predict the ee values of unknown samples. Here, the SVM regression of the mixtures of various enantiomeric compositions in samples of ibuprofen, ketoprofen, and phenylalanine was successful and allowed for simultaneous prediction of multiple enantiomeric compositions. Here, the four sensors provided a very accurate regression analysis with deviations <1.6%. Notably, the presence of carboxylate or phosphate impurities does not preclude an accurate ee determination of chiral carboxylates. Examples to illustrate this point are shown in the ESI.†

**Fig. 9:**
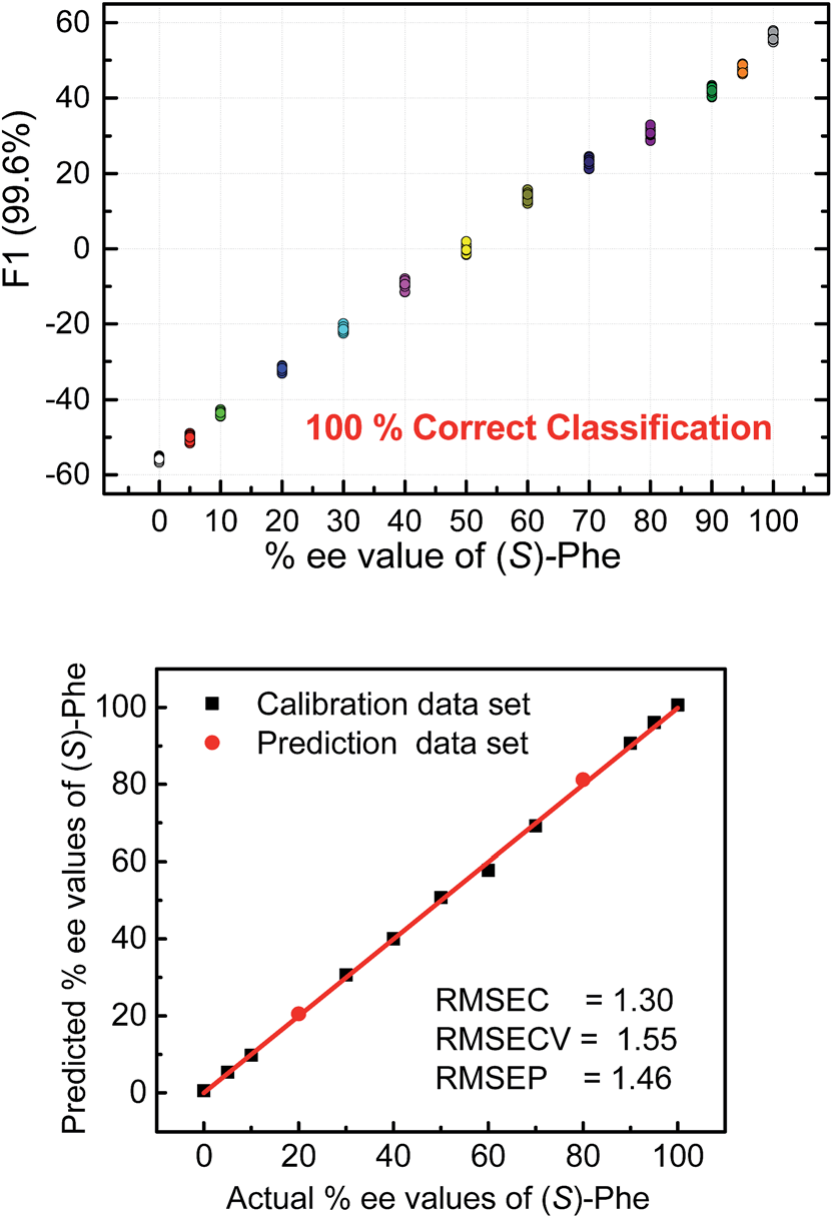
Analysis of the fluorescence signatures from a single chemosensor (**S4**) enables semiquantitative LDA (top) and quantitative SVM analysis (bottom) of the enantiomeric purity in the phenylalanine samples. The low values of root mean square error of prediction 1.5% (RMSEP) confirms high accuracy of the ee analysis. [**S4**] = 40 μM, [(*R*) + (*S*) phenylalanine] = 100 μM.

To further illustrate the chiral recognition powers of the present chemosensors we selected one chemosensor (**S4**), which showed in some cases the best fluorescence response to elucidate the enantiomeric compositions of phenylalanine (Fig. 9 top). Here again, the LDA semiquantitative study revealed full separation of the clusters and 100% correct classification of the individual measurements and a smooth trend suggesting a high potential for successful quantitative analysis. Indeed, SVM linear regression yielded an excellent calibration curve and enabled classification of two samples of unknown enantiomeric purity (Fig. 9 bottom, red circles).

## Conclusions

In summary, we have synthesized four chemosensor derivatives of chirabite-AR. The new macrocyclic chemosensors **S1–S4** comprise a chiral binaphthalene auxiliary modified in the 3,3′-positions with a conjugated moiety for enhanced fluorescence. The new chemosensors bind chiral carboxylates as shown by ESI MS. Furthermore, the binding studies in solution using fluorescence titration experiments show the fluorescence changes depending on the structure and chirality of the analytes thereby providing information-rich response data. The fluorescence output data from four probe arrays were analyzed for analyte recognition and determination of enantiomeric composition. Linear discriminant analysis revealed that the cross reactive probes were able to recognize a number of analytes, namely, enantiomers of ibuprofen, ketoprofen, 2-phenylpropanoate, mandelate, and phenylalanine. Importantly, enantiomers of chiral analytes were also well resolved with 100% correct classification.

Finally, semiquantitative and quantitative experiments were performed aimed at analysis of enantiomeric excess of chiral carboxylates. The quantitative analysis of enantiomeric composition of ibuprofen, ketoprofen, and phenylalanine show that the sensors **S1–S4** are capable of correctly identifying mixtures with varying enantiomeric excess and correctly predict the enantiomeric excess for unknown samples with root mean square error of prediction (RMSEP) <1.6%. This is, to our best knowledge, one of the most accurate determination of ee using optical sensors.^7^,^20^ Furthermore, in some cases a single chemosensor (**S4**) achieved 100% correct classification of the analytes and precise determination of enantiomeric compositions. This method is robust as the presence of anionic impurities such as carboxylates and phosphates did not preclude successful ee determination.

Overall, the present results show that our macrocyclic hosts **S1–S4** display a high potential as fluorescent chemosensors for detection of enantiomeric composition of chiral carboxylates in a high-throughput fashion. Because chirabite-AR can discriminate between enantiomers of a wide range of compounds including carboxylic acids, oxazolidinones, lactones, alcohols, sulfoxides, sulfoximines, isocyanates, and epoxides,^11^ we expect that the new fluorescent derivatives **S1–S4** and other tailored congeners will find wide applicability in the field of microarray sensing.

## Supplementary Material

Supplementary information

Crystal structure data
