# Supplementary material for: Determination of enantiomeric excess of carboxylates by fluorescent macrocyclic sensors
Source: Chem Sci. 2015 Dec 14;7(3):2016–22. doi: 10.1039/c5sc04235f (PMC5968554; doi:10.1039/c5sc04235f)
Supplement: Supplementary file 1 [file SC-007-C5SC04235F-s001.pdf]

Electronic Supplementary Material (ESI) for Chemical Science.
This journal is © The Royal Society of Chemistry 2015

Supplementary Information

Determination of enantiomeric excess of carboxylates by fluorescent macrocyclic sensors

Ali Akdeniz,[†] Tsuyoshi Minami,[†] Sagiri Watanabe,[‡] Maki Yokoyama,[‡] Tadashi Ema,[*,‡] Pavel Anzenbacher, Jr.[*,†]

[†]Department of Chemistry and Center for Photochemical Sciences, Bowling Green State University, Bowling Green, Ohio 43403, USA E-mail: pavel@bgsu.edu

[‡]Division of Applied Chemistry, Graduate School of Natural Science and Technology, Okayama University, Tsushima, Okayama 700-8530, Japan E-mail: ema@cc.okayama-u.ac.jp

Contents

General S2

Synthesis S4

Photophysical properties of S1-S4 S22

Fluorescence titrations S23

Mass spectrometric study of sensor and guest complexes S40

Qualitative linear discriminant analysis (LDA) S41

Quantitative analysis of phenylalanine S43

Quantitative analysis of ibuprofen S45

Quantitative analysis of ketoprofen S50

DFT calculations S51

References S54

General

Mass spectrometry study with complexes was performed using a Shimadzu LCMS-2010A spectrometer (ESI). Solutions for optical measurements were prepared using freshly distilled propionitrile. Photophysical experiments were carried out by using optically dilute solutions. Fluorescence emission spectra were acquired using the Edinburgh single photon counting spectrofluorimeter (FLSP 920). Fluorescence emission spectra were recorded between 350 nm and 500 nm for **S1** and **S2**, 400 nm and 600 nm for **S3** and **S4**. Fluorescence measurements were performed on a single photon counting spectrofluorimeter from Edinburgh Analytical Instruments (FL/FS 920). The emission from probes was scanned in 2 nm steps. The dwell time was 0.30 sec. Scans were taken under ambient room conditions. Guest titrations were performed in propionitrile (Host concentrations: [**S1**], [**S2**], [**S3**] = 20 μM, [**S4**] = 40 μM). Sensor solutions were excited at 330 nm for **S1**, 335 nm for **S2**, 375 nm for **S3**, 330 nm for **S4**, respectively. Titration isotherms were constructed from changes in the fluorescence maximum at 372 nm for **S1**, 380 nm for **S2**, 492 nm for **S3**, 470 nm for **S4**, respectively. Binding constants were calculated by nonlinear least-square methods using an equation for 1:1 binding model.[1] All the calculations were performed using Origin.[2]

$$[G] = \frac{K_a[G]_t - K_a[S]_t - 1 + \{(K_a[S]_t - K_a\,[G]_t + 1)^2 + 4K_a\,[G]_t\}^{1/2}}{2K_a}$$

$$[SG] = \frac{K_a[G][S]_t}{1 + K_a[G]} = \frac{F - F_0}{F_i - F_0}$$

K_a: Binding constant
$[S]_t$: Concentration of sensor
$[G]_t$: Concentration of the guest
$[G]$: Unknown concentration of Guest
$[SG]$: Unknown concentration of complex
$[F]$: Guest-dependent fluorescence
$[F]_0$: Fluorescence intensity of sensor only
$[F]_i$: Saturated fluorescence intensity of sensor

Quantum yield measurements were acquired using a Hamamatsu absolute quantum yield spectrometer C11347.

The array experiments were performed in 384-well plates. Each experiment was performed in 12 repetitions. Each well received 35 µL sensor-guest solution in propionitrile after 10 min incubation. The plates were measured immediately after pipetting by BMG CLARIOstar. The coefficient of variability (CV) among the data within the class of 12 repetitions was lower than 1%. Thus, obtained data for quantitative and semi-quantitative analysis were then analyzed using linear discriminant analysis (LDA) without any further pretreatment. A support vector machine (SVM) with preprocessing of LDA and a log decay scaling (scale = 0.1450) was used.

Synthetic Procedures for S1–S4.

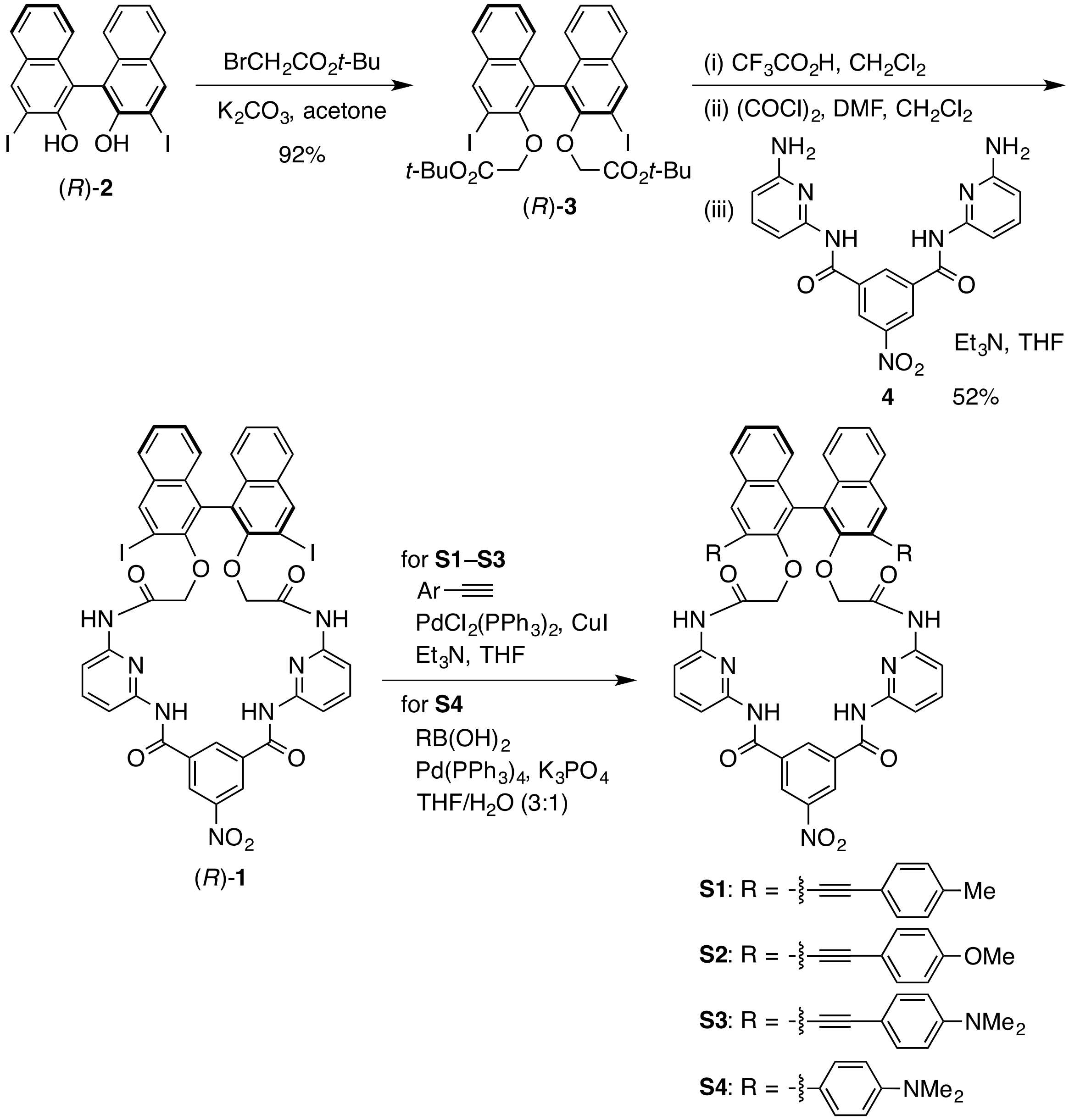

(*R*)-2,2'-Bis[(*tert*-butoxycarbonyl)methoxy]-3,3'-diiodo-1,1'-binaphthyl ((*R*)-3). A mixture of (*R*)-**2** (7.19 g, 13.4 mmol),[3] *tert*-butyl bromoacetate (4.11 mL, 28.0 mmol), and K_2CO_3 (4.06 g, 29.4 mmol) in acetone (80 mL) was heated at reflux for 13 h under N_2. The mixture was filtered and concentrated. Purification by silica gel column chromatography (hexane/EtOAc (5:1)) gave (*R*)-**3** as a white solid (9.37 g, 92%): $[\alpha]^{28}_{D}$ –22.0 (*c* 1.00, $CHCl_3$); ^1H NMR (d_6-acetone, 400 MHz) δ 1.23 (s, 18H), 4.16 (d, J = 15.4 Hz, 2H), 4.59 (d, J = 15.5 Hz, 2H), 7.01 (dd, J = 1.0, 8.5

Hz, 2H), 7.35 (dt, J = 1.4, 7.7 Hz, 2H), 7.50 (dt, J = 1.2, 7.6 Hz, 2H), 7.97 (d, J = 8.2 Hz, 2H), 8.67 (s, 2H); ^{13}C NMR (d_6-acetone, 100 MHz) δ 28.1, 70.9, 81.6, 92.2, 126.3, 126.4, 126.9, 128.1, 128.3, 133.5, 134.5, 141.0, 154.2, 167.2; IR (KBr) 3067, 2978, 2911, 1753, 1564, 1412, 1393, 1368, 1348, 1090, 1070, 1051, 1022, 1001, 889, 881, 851, 802, 748, 613 cm^{-1}; Anal. Calcd for $C_{32}H_{32}I_2O_6$: C, 50.15; H, 4.21. Found: C, 50.25; H, 4.29; HRMS (FAB) calcd for $C_{32}H_{33}I_2O_6$ 767.0366, found 767.0372 ([M + H]$^+$).

Chiral Macrocycle (*R*)-1. To a solution of diester (*R*)-**3** (12.0 g, 15.6 mmol) in CH_2Cl_2 (80 mL) was added CF_3CO_2H (28 mL), and the mixture was stirred at room temperature for 18 h. The solution was concentrated and dried in vacuo to give the corresponding diacid as a yellow solid. To a suspension of the diacid (5.11 g, 7.81 mmol) in dry CH_2Cl_2 (145 mL) were added $(COCl)_2$ (7.3 mL, 86 mmol) and DMF (2 drop). The mixture was stirred at room temperature for 14 h. The volatiles were removed by rotary evaporation, and the residue was dried in vacuo for 3 h. The obtained acid chloride was used without further purification. A solution of the acid chloride (5.40 g, 7.81 mmol) in dry THF (250 mL) and a solution of diamine **4**[4] (3.69 g, 9.37 mmol) and Et_3N (2.2 mL, 16 mmol) in dry THF (250 mL) were added dropwise simultaneously to dry THF (100 mL) at room temperature with vigorous stirring over 6 h. The mixture was stirred for an additional 13 h, and the volatiles were removed by rotary evaporation. The solid residue was dissolved in CH_2Cl_2, and the solution was washed with brine, dried over Na_2SO_4, and concentrated. Purification by silica gel column chromatography (CH_2Cl_2/THF (30:1)) gave (*R*)-**1** as a yellow solid (4.13 g, 52%): $[\alpha]^{27}_{D}$ +170 (c 1.00, $CHCl_3$); ^{1}H NMR (d_6-acetone, 400 MHz) δ 3.75 (d, J = 15.1 Hz, 2H), 4.40 (d, J = 15.1 Hz, 2H), 7.29 (dd, J = 0.9, 8.5 Hz, 2H), 7.51 (dt, J = 1.3, 7.7 Hz, 2H), 7.63 (dt, J = 1.3, 7.6 Hz, 2H), 7.85 (dd, J = 0.9, 8.0 Hz, 2H), 7.91 (t, J = 8.0 Hz, 2H), 8.08 (d, J = 8.2 Hz, 2H), 8.13 (dd, J = 0.9, 7.8 Hz, 2H), 8.77 (s, 2H), 8.83 (s, 2H), 8.98 (d, J

= 1.5 Hz, 2H), 9.64 (t, J = 1.4 Hz, 1H), 10.15 (s, 2H); ^{13}C NMR ($CDCl_3$, 100 MHz) δ 71.7, 90.7, 109.8, 110.3, 125.3, 125.7, 126.9, 127.0, 127.5, 128.3, 128.8, 132.6, 133.1, 135.7, 140.9, 141.4, 148.5, 149.0, 149.9, 151.5, 161.0, 166.0; IR (KBr) 3393, 3059, 2914, 1697, 1585, 1456, 1395, 1342, 1315, 1244, 1221, 1150, 1080, 1051, 1022, 889, 802, 752, 737, 719 cm^{-1}; HRMS (FAB) calcd for $C_{42}H_{28}I_2N_7O_8$ 1012.0089, found 1012.0082 ($[M + H]^+$).

Chiral Macrocycle S1. A mixture of (R)-**1** (253 mg, 0.250 mmol), p-tolylacetylene (152 μL, 1.20 mmol), $PdCl_2(PPh_3)_2$ (8.80 mg, 12.5 μmol), and CuI (2.46 mg, 12.9 μmol) in degassed THF (5 mL) and Et_3N (0.75 mL) was heated at 65 °C for 5 h under N_2. The reaction mixture was filtered, and the volatiles were removed by rotary evaporation. Purification by silica gel column chromatography (CH_2Cl_2/THF (100:1)) gave **S1** as a yellow solid (179 mg, 73%): mp 271 °C; $[\alpha]^{28}_D$ +187 (c 1.00, $CHCl_3$); ^{1}H NMR ($CDCl_3$, 400 MHz) δ 2.23 (s, 6H), 4.03 (d, J = 15.4 Hz, 2H), 4.64 (d, J = 15.2 Hz, 2H), 6.87 (d, J = 7.8 Hz, 4H), 7.17 (d, J = 7.6 Hz, 4H), 7.25 (d, J = 10.3 Hz, 2H), 7.40 (t, J = 7.6 Hz, 2H), 7.51 (t, J = 7.5 Hz, 2H), 7.73 (t, J = 8.0 Hz, 2H), 7.88 (d, J = 7.5 Hz, 2H), 7.90 (d, J = 7.3 Hz, 2H), 8.01 (d, J = 8.0 Hz, 2H), 8.26 (s, 2H), 8.47 (s, 1H), 8.70 (s, 2H), 9.07 (s, 2H), 9.11 (s, 2H); ^{13}C NMR ($CDCl_3$, 100 MHz) δ 21.5, 72.7, 84.2, 95.6, 109.6, 110.4, 116.8, 119.0, 125.3, 125.4, 126.6, 127.2, 127.8, 128.1, 128.4, 129.1, 131.0, 131.3, 133.3, 134.8, 135.9, 139.2, 141.2, 148.89, 148.94, 150.2, 153.3, 160.8, 167.2; IR (KBr) 3391, 3080, 3065, 3028, 2916, 2872, 2210, 1699, 1585, 1539, 1512, 1452, 1423, 1346, 1306, 1244, 1206, 1150, 1086, 1040, 1022, 993, 910, 897, 816, 802, 746, 731, 719 cm^{-1}; HRMS (FAB) calcd for $C_{60}H_{42}N_7O_8$ 988.3095, found 988.3094 ($[M + H]^+$).

Chiral Macrocycle S2. A mixture of (R)-**1** (509 mg, 0.503 mmol), 4-ethynylanisole (0.30 mL, 2.3 mmol), $PdCl_2(PPh_3)_2$ (17.8 mg, 25.4 μmol), and CuI (4.72 mg, 24.8 μmol) in degassed THF

(10 mL) and Et_3N (1.5 mL) was heated at 50 °C for 5 h under Ar. The reaction mixture was filtered, and the volatiles were removed by rotary evaporation. Purification by silica gel column chromatography (CH_2Cl_2/THF (30:1)) gave **S2** as a yellow solid (471 mg, 92%): mp 274 °C (dec); $[\alpha]^{27}_{D}$ +153 (*c* 1.00, $CHCl_3$); ^1H NMR ($CDCl_3$, 400 MHz) δ 3.70 (s, 6H), 4.02 (d, *J* = 15.4 Hz, 2H), 4.63 (d, *J* = 15.4 Hz, 2H), 6.58 (d, *J* = 8.9 Hz, 4H), 7.21 (d, *J* = 8.9 Hz, 4H), 7.24 (d, *J* = 9.0 Hz, 2H), 7.40 (dt, *J* = 1.3, 7.8 Hz, 2H), 7.50 (dt, *J* = 1.1, 7.5 Hz, 2H), 7.72 (t, *J* = 8.1 Hz, 2H), 7.87 (d, *J* = 7.6 Hz, 2H), 7.89 (d, *J* = 7.9 Hz, 2H), 8.01 (d, *J* = 7.5 Hz, 2H), 8.23 (s, 2H), 8.61 (s, 1H), 8.87 (s, 2H), 9.02 (s, 2H), 9.09 (d, *J* = 1.3 Hz, 2H); ^{13}C NMR ($CDCl_3$, 100 MHz) δ 55.3, 72.6, 83.6, 95.6, 109.7, 110.5, 114.0, 114.1, 116.9, 125.3, 125.5, 126.7, 127.3, 128.0, 128.2, 128.4, 131.0, 133.0, 133.2, 134.7, 135.9, 141.4, 148.9, 149.1, 150.1, 153.2, 160.0, 161.1, 167.2; IR (KBr) 3391, 3086, 3057, 2955, 2916, 2835, 2207, 1697, 1605, 1585, 1539, 1510, 1452, 1425, 1346, 1306, 1248, 1207, 1173, 1150, 1105, 1086, 1024, 993, 953, 893, 831, 802, 748, 729, 719, 692 cm^{-1}; HRMS (FAB) calcd for $C_{60}H_{42}N_7O_{10}$ 1020.2993, found 1020.2972 ($[M + H]^+$).

Chiral Macrocycle S3. A mixture of (*R*)-**1** (253 mg, 0.250 mmol), 4-ethynyl-*N*,*N*-dimethylaniline (175 mg, 1.20 mmol), $PdCl_2(PPh_3)_2$ (8.81 mg, 12.5 μmol), and CuI (2.39 mg, 12.5 μmol) in degassed THF (5 mL) and Et_3N (0.75 mL) was heated at 65 °C for 5 h under N_2. The reaction mixture was filtered, and the volatiles were removed by rotary evaporation. Purification by silica gel column chromatography (CH_2Cl_2/THF (50:1)) gave **S3** as a brown solid (189 mg, 72%): mp 221–224 °C (dec); $[\alpha]^{27}_{D}$ +54.2 (*c* 1.02, $CHCl_3$); ^1H NMR ($CDCl_3$, 400 MHz) δ 2.86 (s, 12H), 4.00 (d, *J* = 15.4 Hz, 2H), 4.66 (d, *J* = 15.4 Hz, 2H), 6.36 (d, *J* = 8.5 Hz, 4H), 7.14 (d, *J* = 8.8 Hz, 4H), 7.23 (d, *J* = 8.4 Hz, 2H), 7.37 (t, *J* = 7.6 Hz, 2H), 7.49 (t, *J* = 7.5 Hz, 2H), 7.73 (t, *J* = 8.1 Hz, 2H), 7.88 (d, *J* = 7.6 Hz, 2H), 7.90 (d, *J* = 8.0 Hz, 2H), 8.02 (d, *J* = 8.0 Hz, 2H), 8.20 (s, 2H), 8.59 (s, 1H), 8.95 (s, 2H), 9.06 (s, 2H), 9.11 (s, 2H); ^{13}C NMR ($CDCl_3$,

100 MHz) δ 40.0, 72.5, 83.1, 97.2, 108.5, 109.6, 110.4, 111.6, 117.5, 125.2, 125.5, 126.4, 127.2, 127.7, 128.1, 128.3, 131.1, 132.7, 132.9, 134.3, 136.1, 141.1, 149.0, 149.1, 150.16, 150.23, 153.1, 161.1, 167.3; IR (KBr) 3385, 3090, 3057, 2913, 2884, 2860, 2803, 2197, 1697, 1653, 1609, 1585, 1558, 1522, 1452, 1425, 1362, 1348, 1314, 1244, 1219, 1190, 1165, 1150, 1084, 1040, 1022, 993, 945, 912, 893, 816, 802, 746, 719 cm^{-1}; HRMS (FAB) calcd for $C_{62}H_{48}N_9O_8$ 1046.3626, found 1046.3628 ($[M + H]^+$).

Chiral Macrocycle S4. A mixture of (*R*)-**1** (102 mg, 0.101 mmol), 4-(dimethylamino)phenylboronic acid (52.7 mg, 0.303 mmol), K_3PO_4 (65.6 mg, 0.309 mmol), and $Pd(PPh_3)_4$ (11.4 mg, 9.86 μmol) in degassed THF/H_2O (3:1) (1.2 mL) was heated at 65 °C for 6 h under Ar. The volatiles were removed by rotary evaporation, and the solid residue was dissolved in CH_2Cl_2. The solution was washed with brine, dried over Na_2SO_4, and concentrated. Purification by silica gel column chromatography (CH_2Cl_2/THF (30:1)) gave **S4** as a dark orange solid (95.9 mg, 95%): $[\alpha]^{23}_{D}$ –81.6 (*c* 1.01, $CHCl_3$); ^1H NMR ($CDCl_3$, 400 MHz) δ 2.72 (s, 12H), 3.72 (d, *J* = 15.4 Hz, 2H), 3.86 (d, *J* = 15.4 Hz, 2H), 6.69 (s, 4H), 7.21 (d, *J* = 8.5 Hz, 2H), 7.33 (dt, *J* = 1.1, 7.7 Hz, 2H), 7.46–7.52 (m, 6H), 7.74 (d, *J* = 4.2 Hz, 4H), 7.92 (d, *J* = 8.2 Hz, 2H), 7.95 (s, 2H), 8.06 (t, *J* = 4.4 Hz, 2H), 8.49 (s, 2H), 8.90 (s, 1H), 9.17 (d, *J* = 1.1 Hz, 2H), 9.26 (s, 2H); ^{13}C NMR ($CDCl_3$, 100 MHz) δ 40.3, 71.6, 109.6, 110.4, 112.5, 125.4, 125.6, 125.9, 126.8, 127.2, 128.2, 128.8, 130.0, 130.8, 131.4, 132.7, 134.7, 136.0, 141.3, 148.7, 149.0, 149.8, 150.0, 152.1, 161.3, 166.7; IR (KBr) 3375, 3086, 2911, 1701, 1611, 1585, 1526, 1452, 1418, 1346, 1314, 1246, 1223, 1200, 1150, 1080, 1053, 1022, 820, 802, 750, 735, 719 cm^{-1}; Anal. Calcd for $C_{58}H_{47}N_9O_8$: C, 69.80; H, 4.75; N, 12.63. Found: C, 69.55; H, 4.77; N, 12.33; HRMS (FAB) calcd for $C_{58}H_{48}N_9O_8$ 998.3626, found 998.3600 ($[M + H]^+$).

^{1}H and ^{13}C NMR Spectra

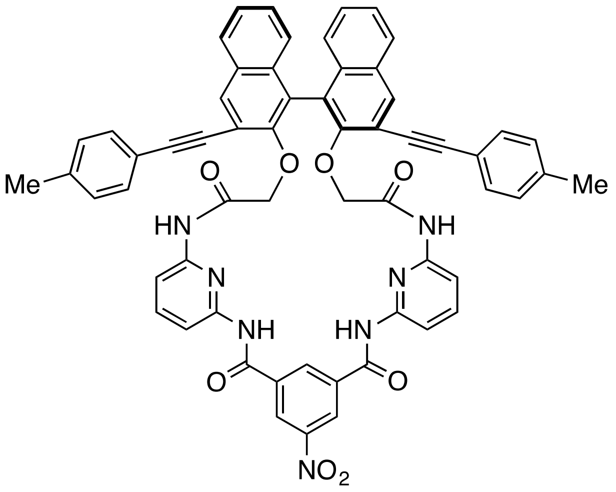

S1

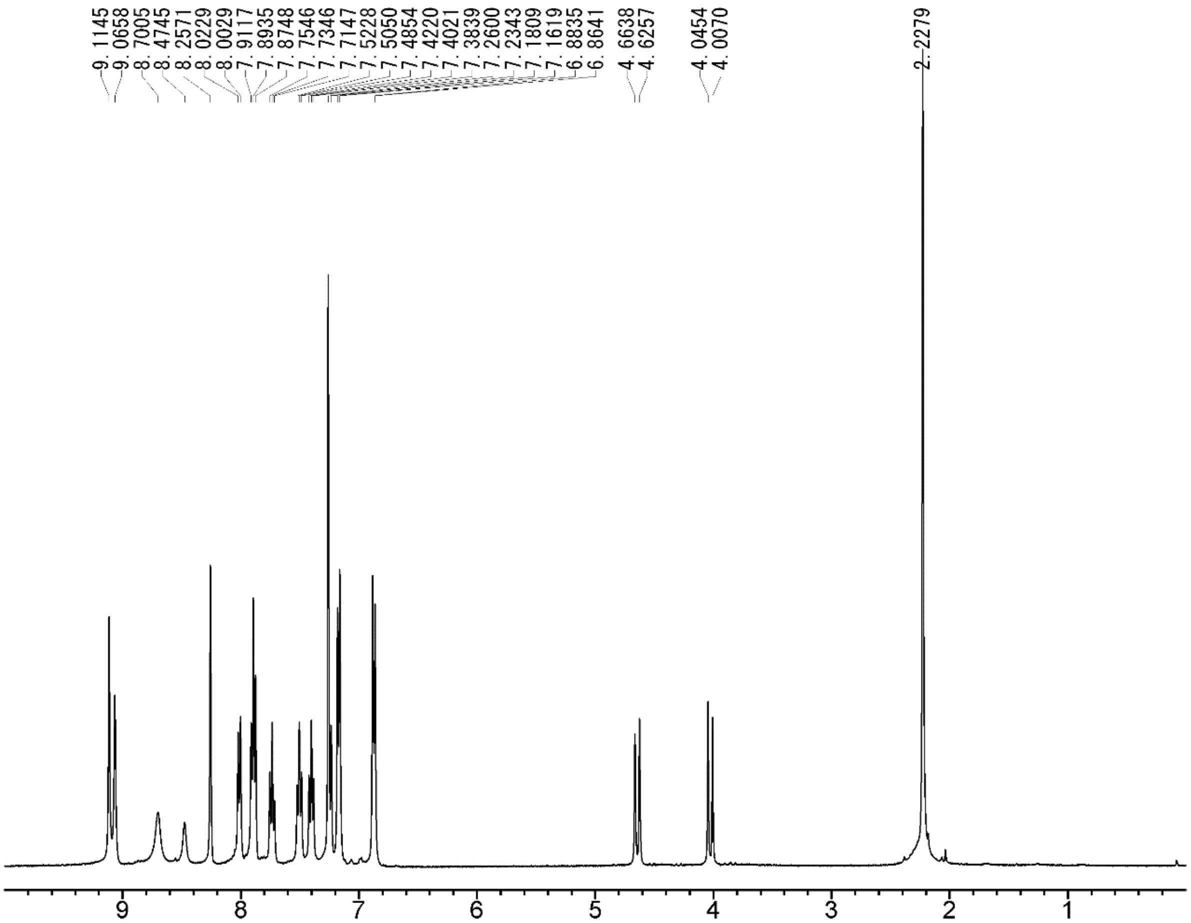

400 MHz ^{1}H NMR spectrum of **S1** in $CDCl_3$.

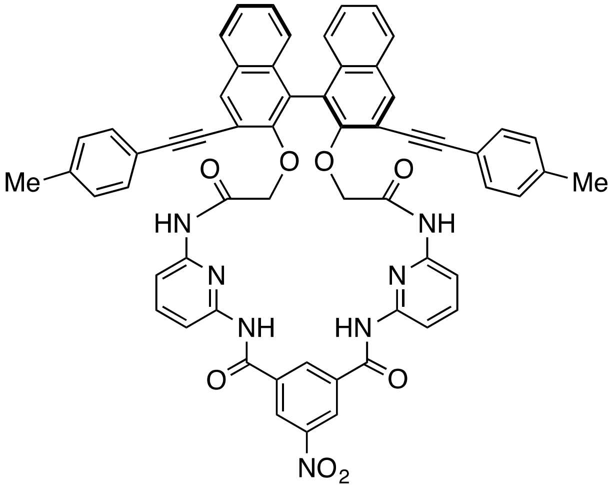

S1

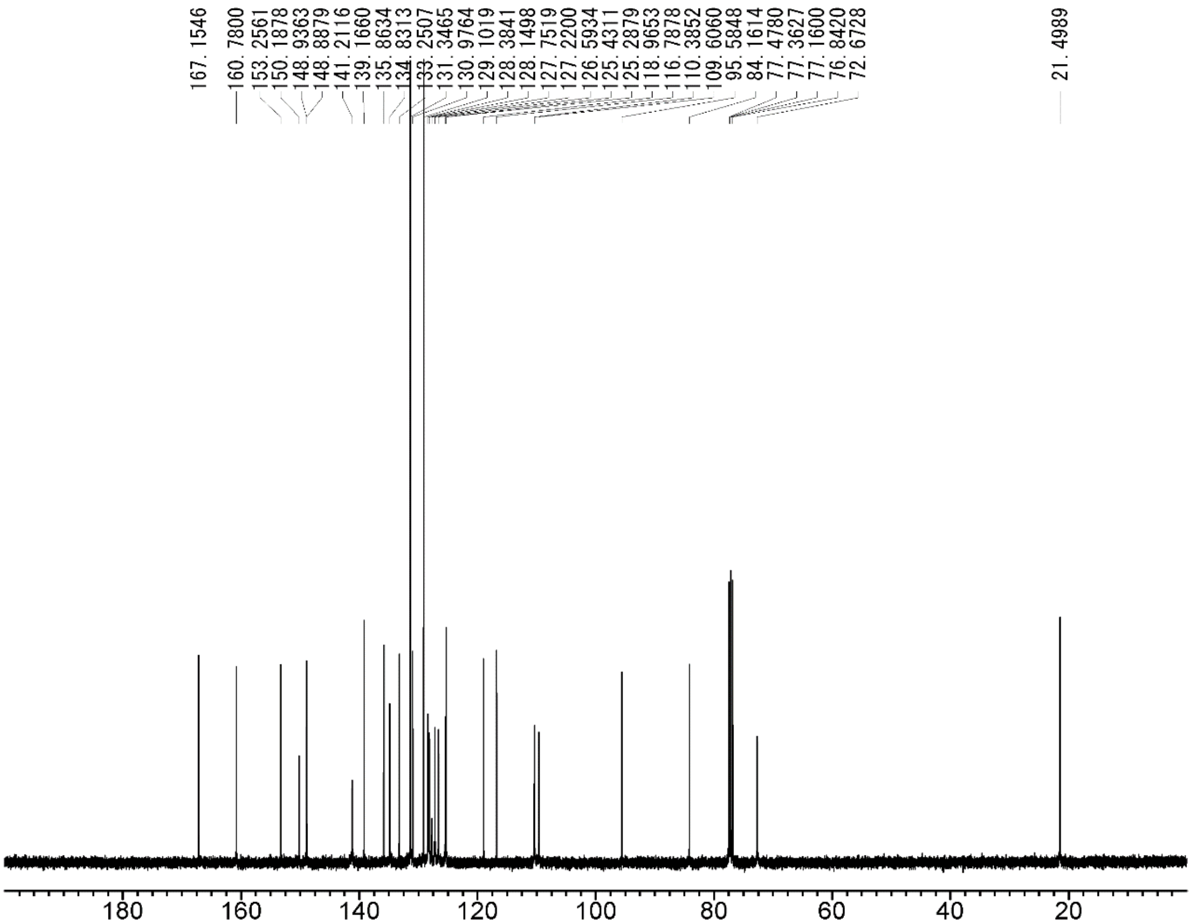

100 MHz ^{13}C NMR spectrum of **S1** in $CDCl_3$.

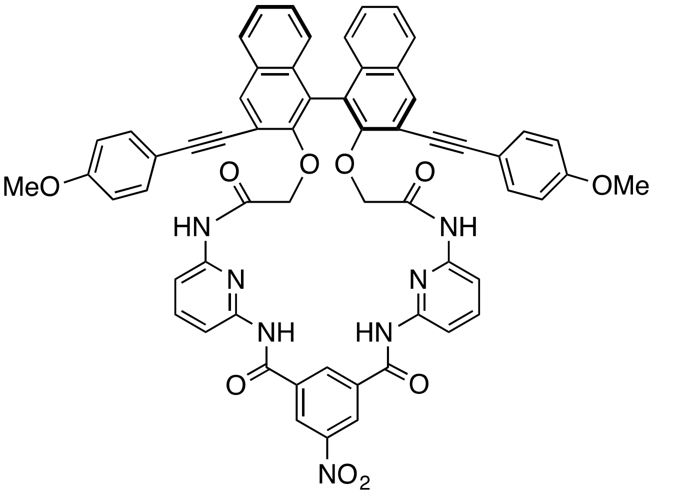

S2

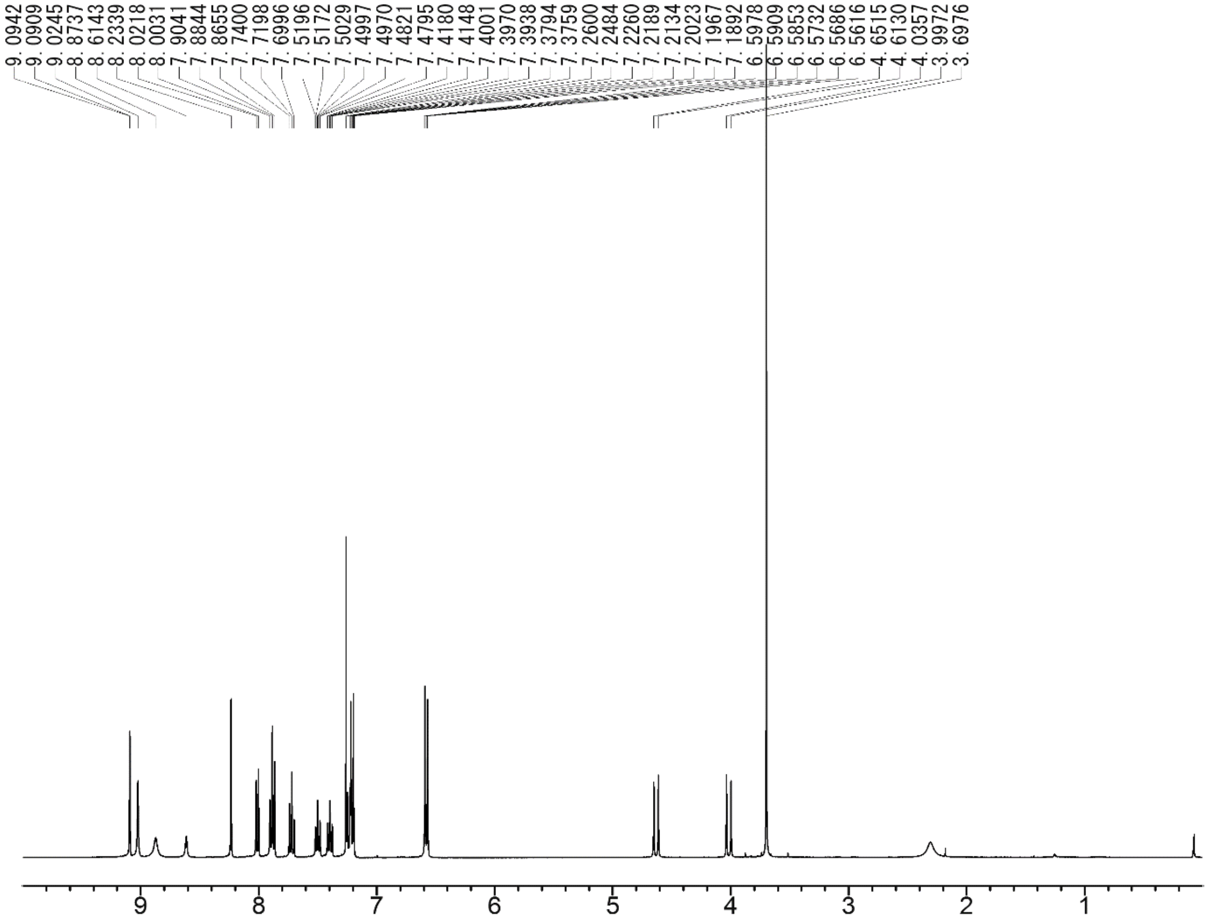

400 MHz ^{1}H NMR spectrum of **S2** in $CDCl_3$.

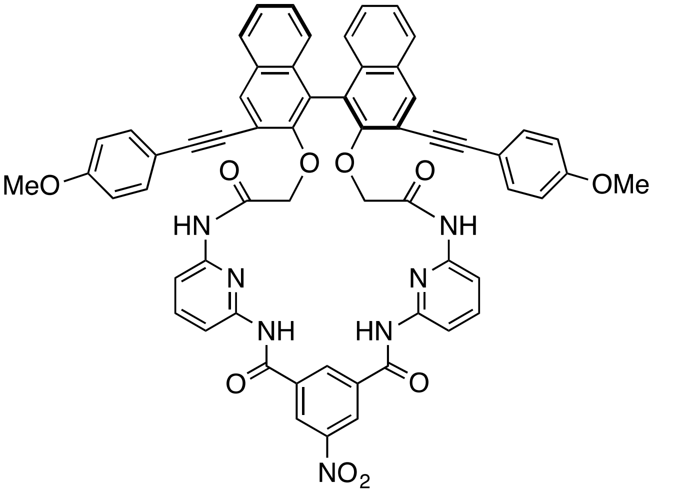

S2

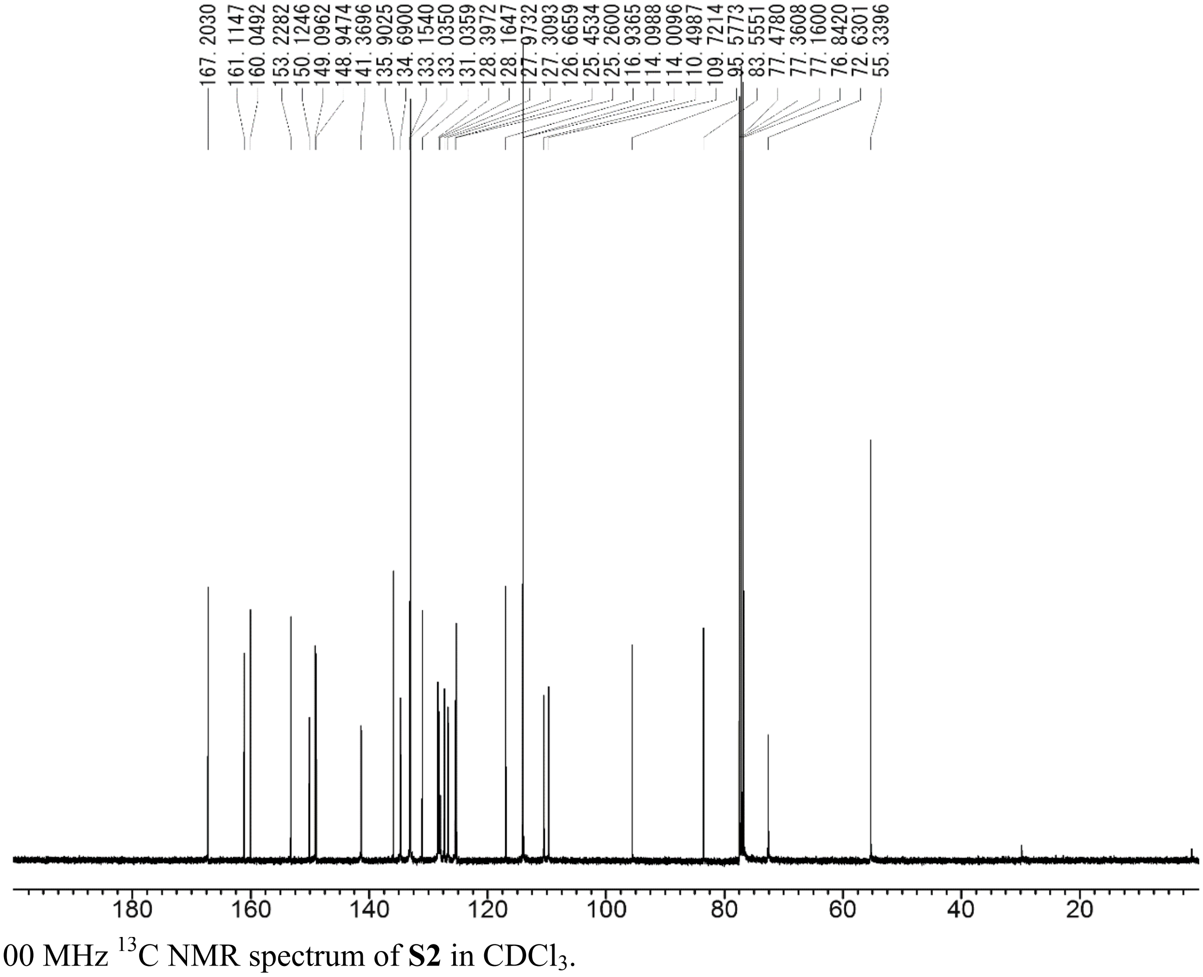

100 MHz ^{13}C NMR spectrum of **S2** in $CDCl_3$.

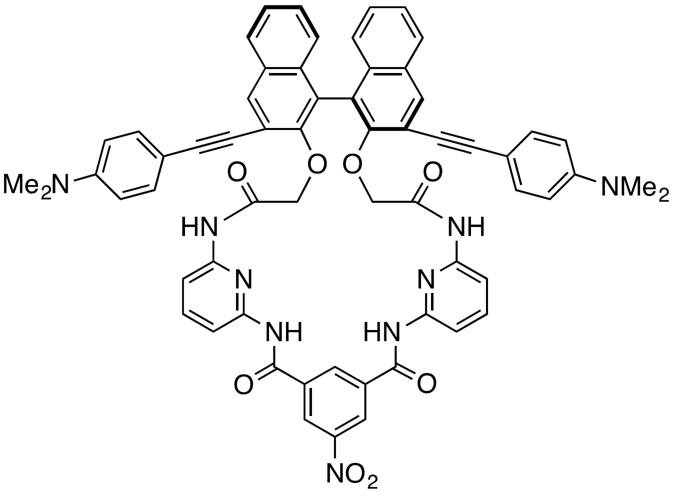

S3

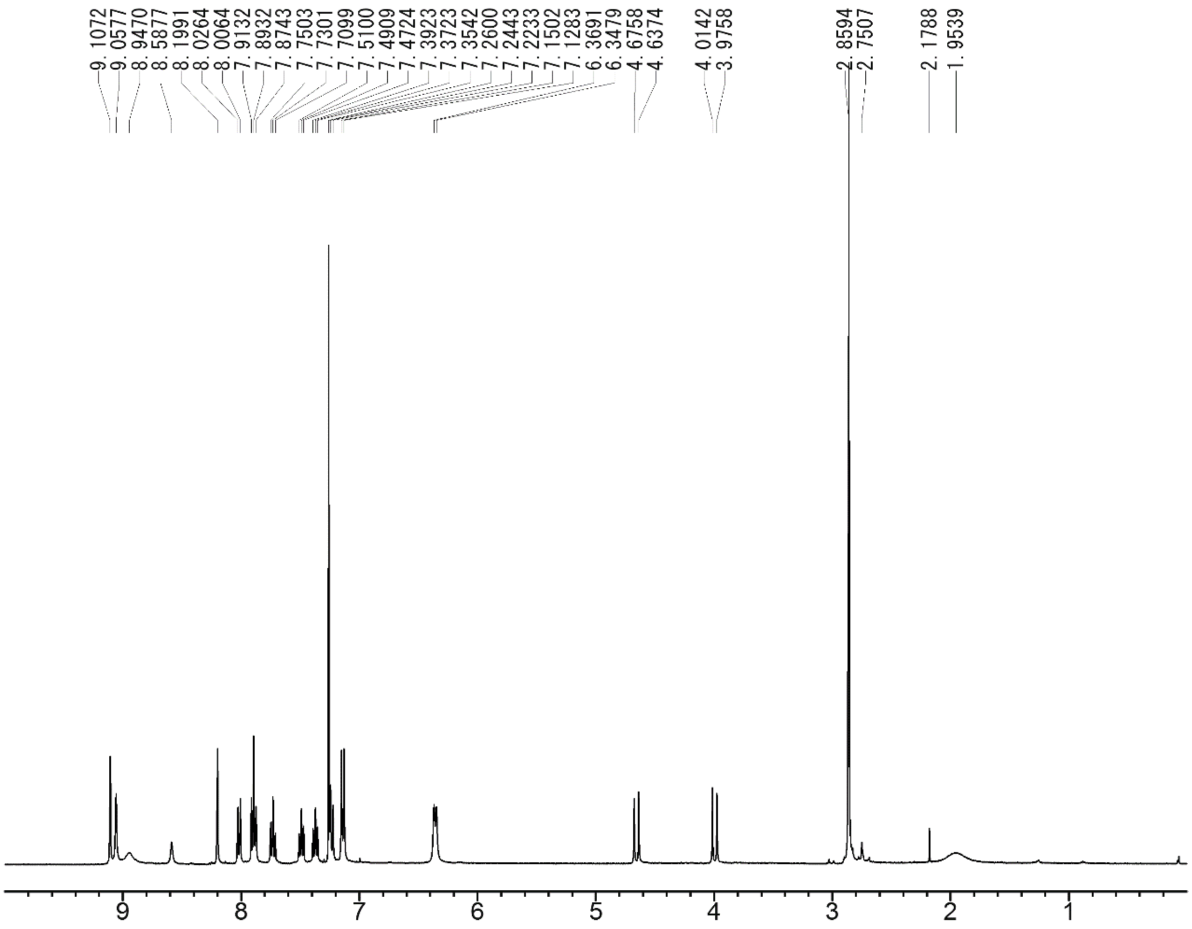

400 MHz ^{1}H NMR spectrum of **S3** in $CDCl_3$.

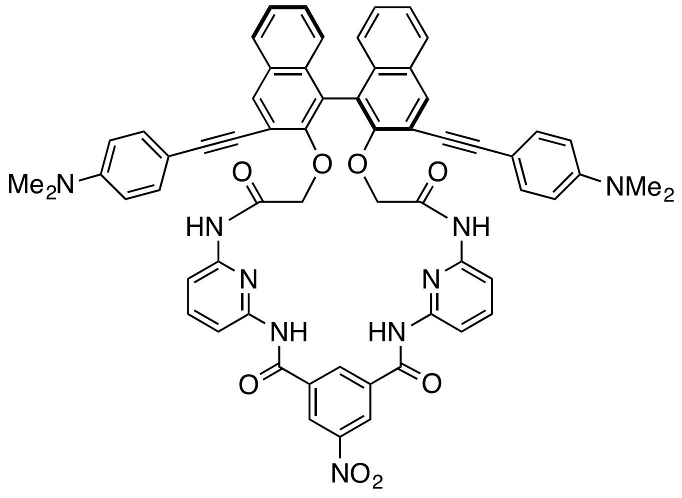

S3

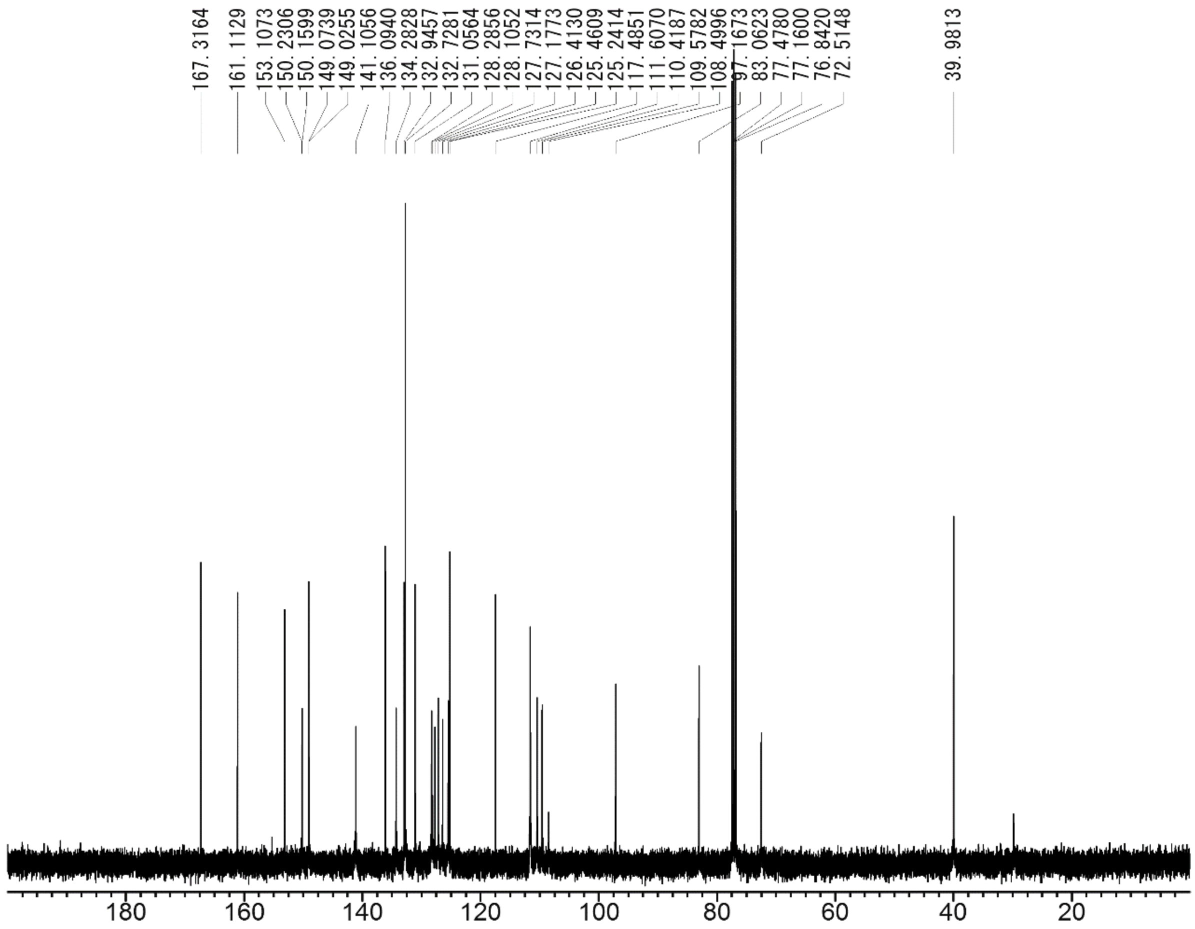

100 MHz ^{13}C NMR spectrum of **S3** in $CDCl_3$.

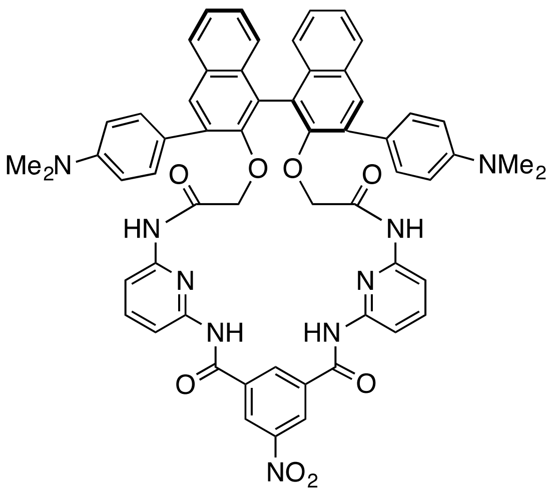

S4

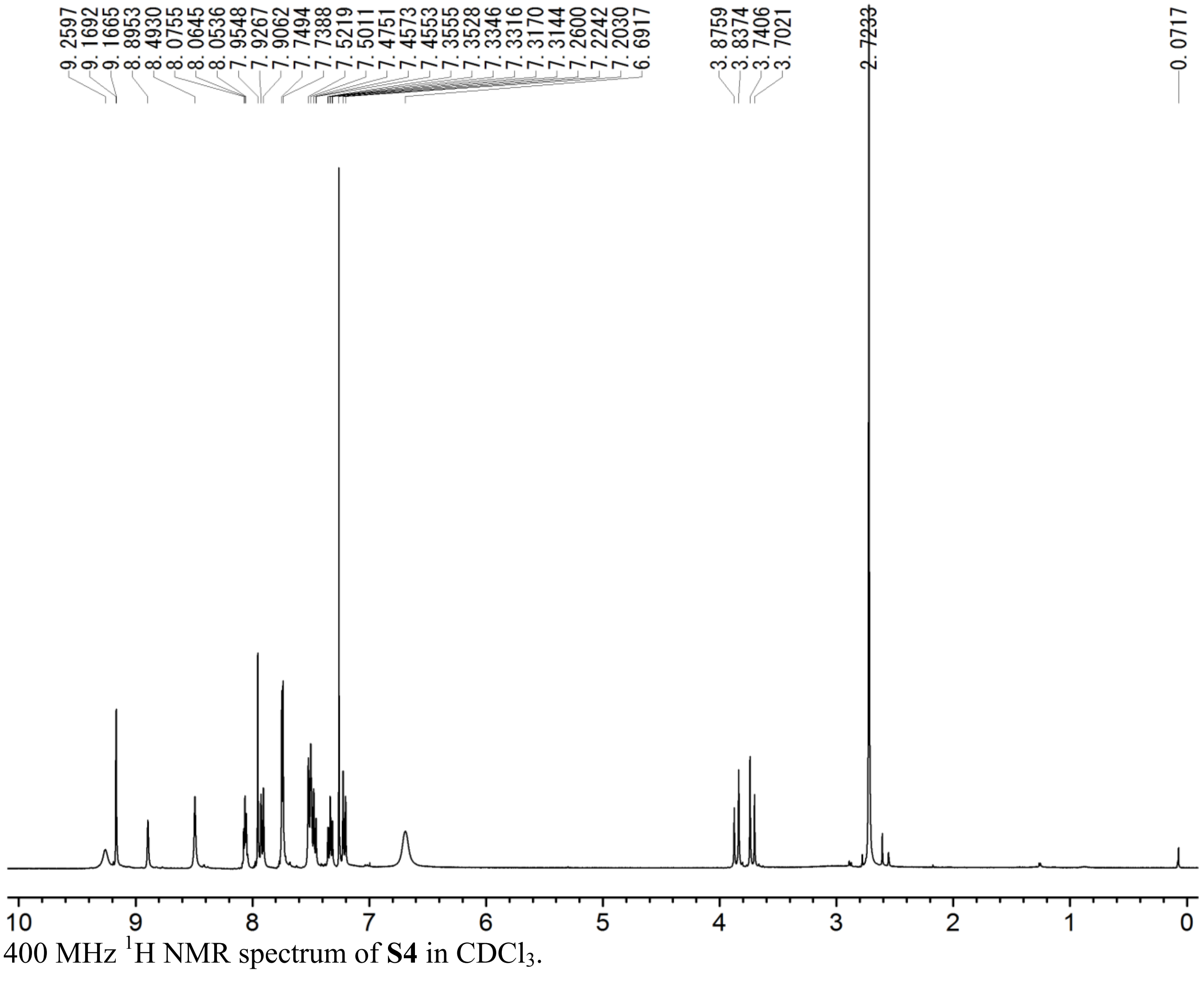

400 MHz ^{1}H NMR spectrum of **S4** in $CDCl_3$.

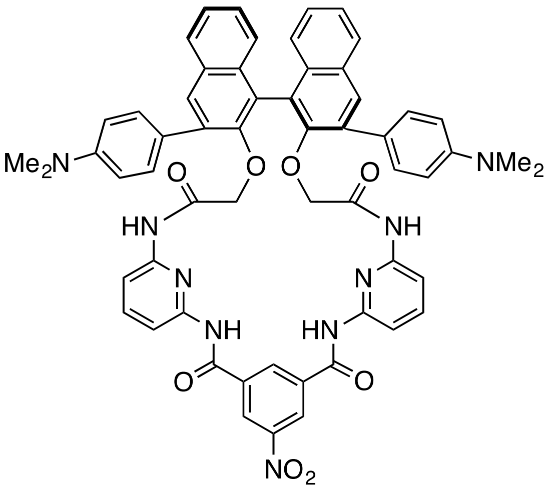

S4

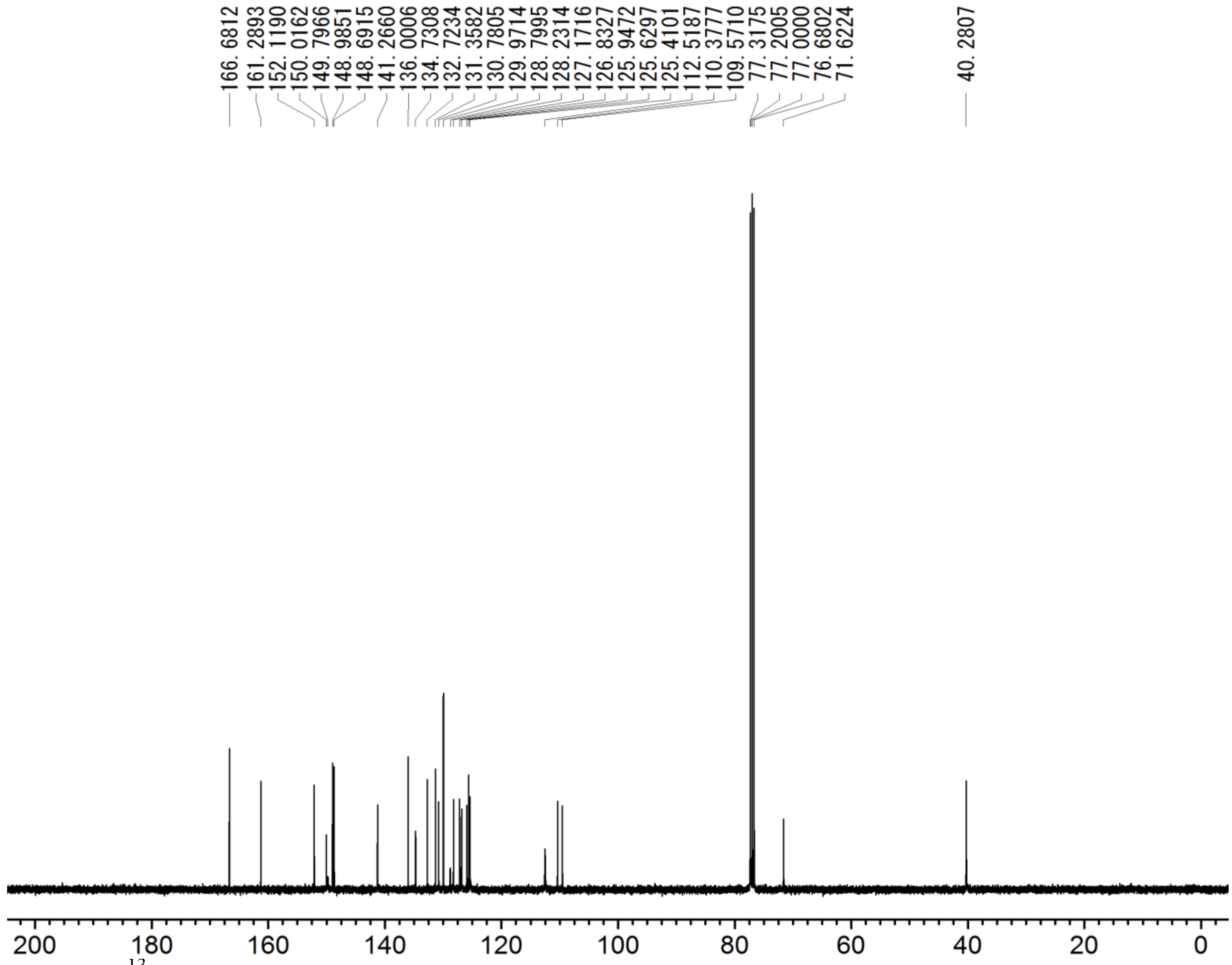

100 MHz ^{13}C NMR spectrum of **S4** in $CDCl_3$.

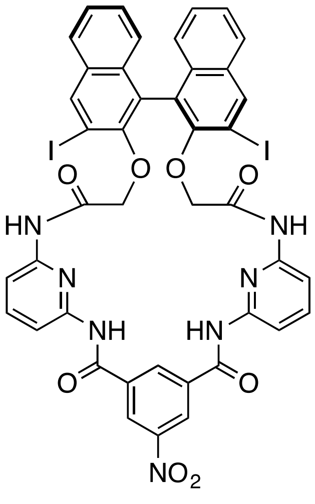

(*R*)-**1**

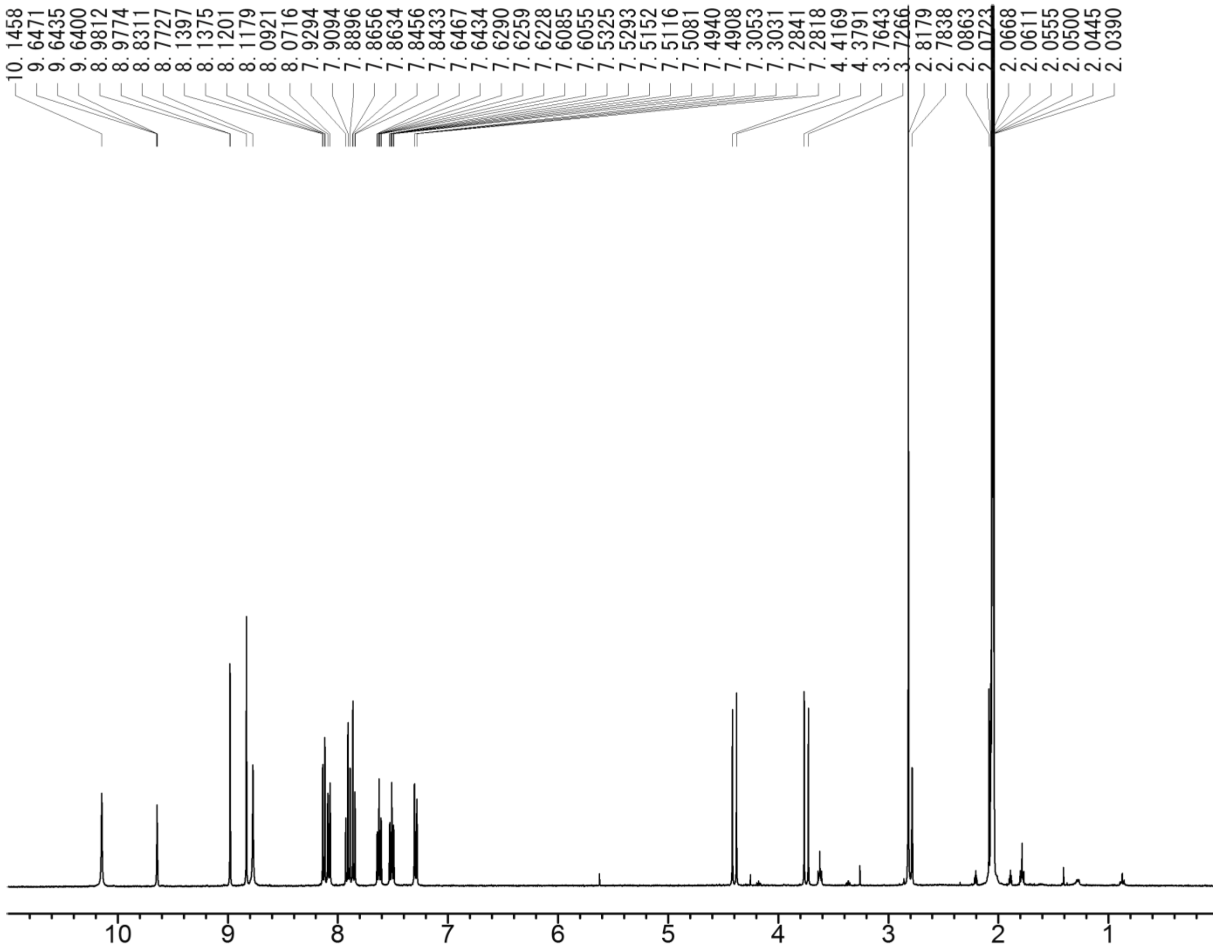

400 MHz ^{1}H NMR spectrum of (*R*)-**1** in d_6-acetone.

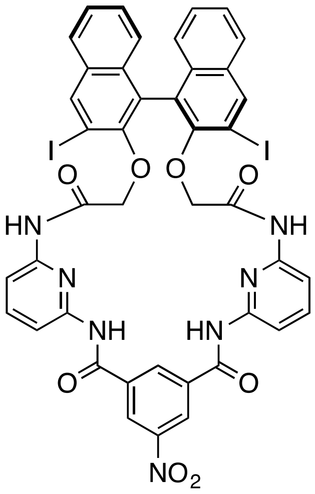

(*R*)-**1**

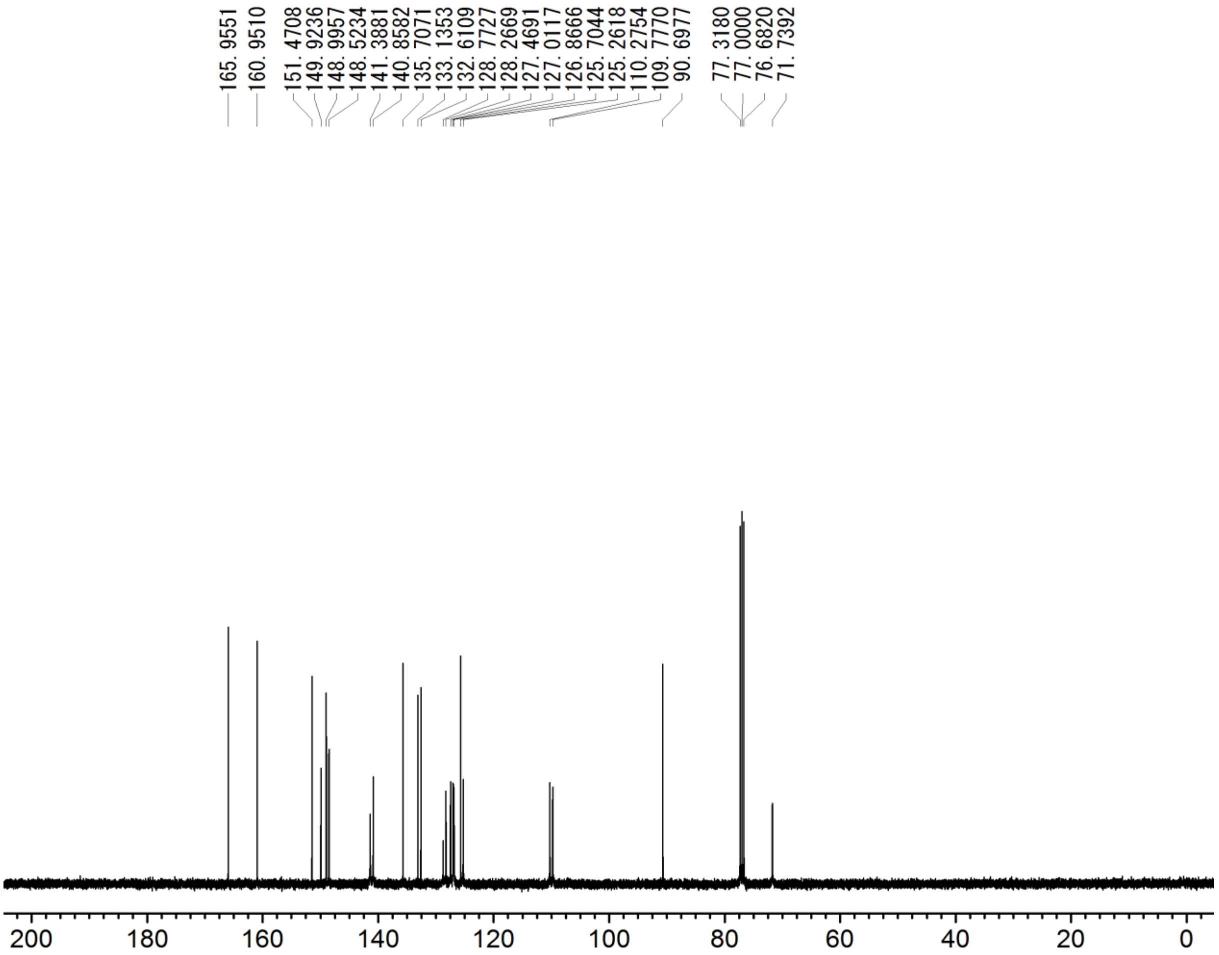

100 MHz ^{13}C NMR spectrum of (*R*)-**1** in $CDCl_3$.

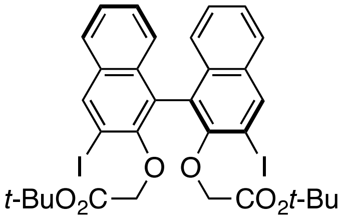

(*R*)-**3**

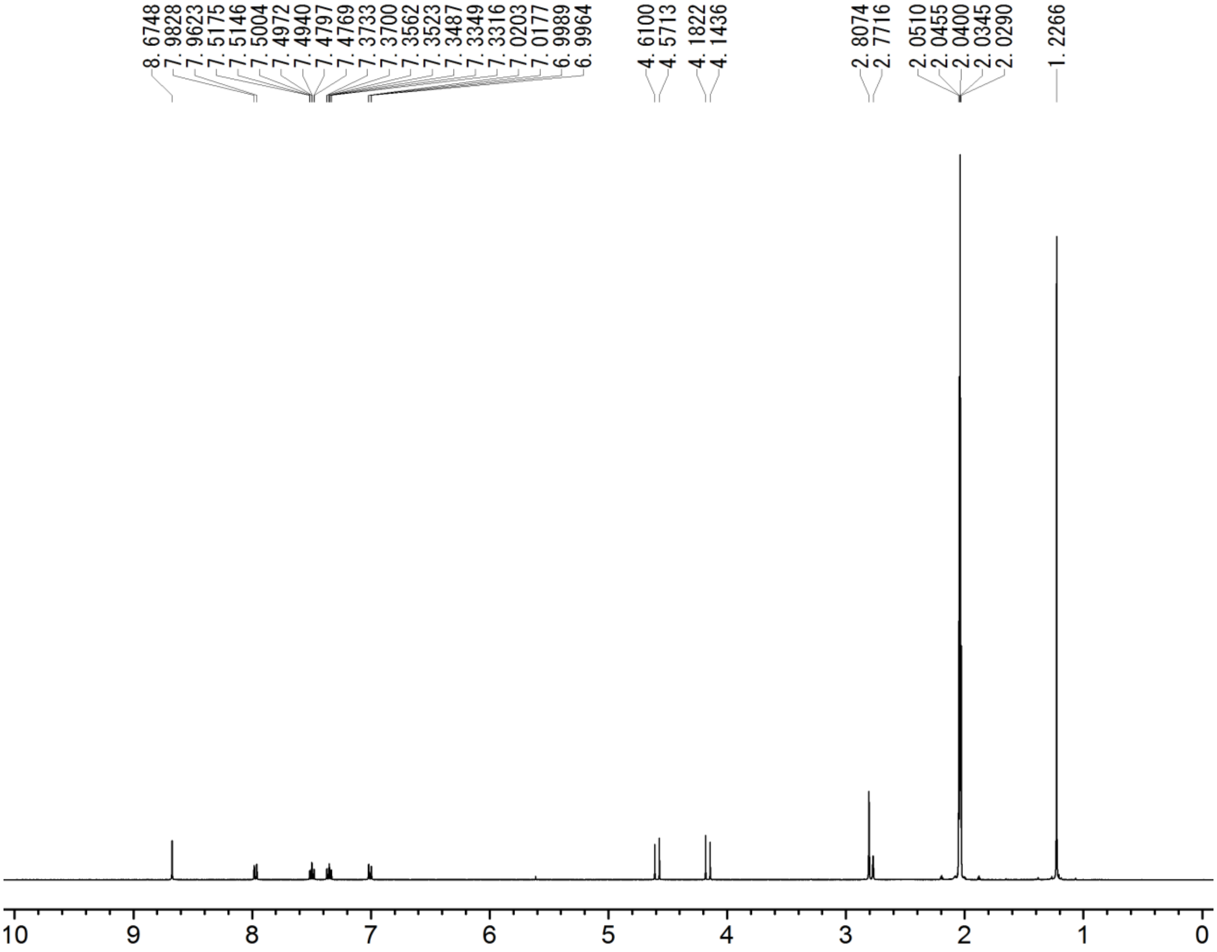

400 MHz ^{1}H NMR spectrum of (*R*)-**3** in d_6-acetone.

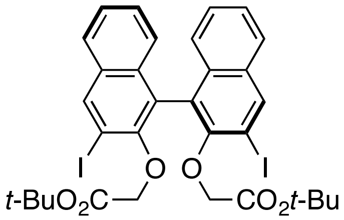

(*R*)-**3**

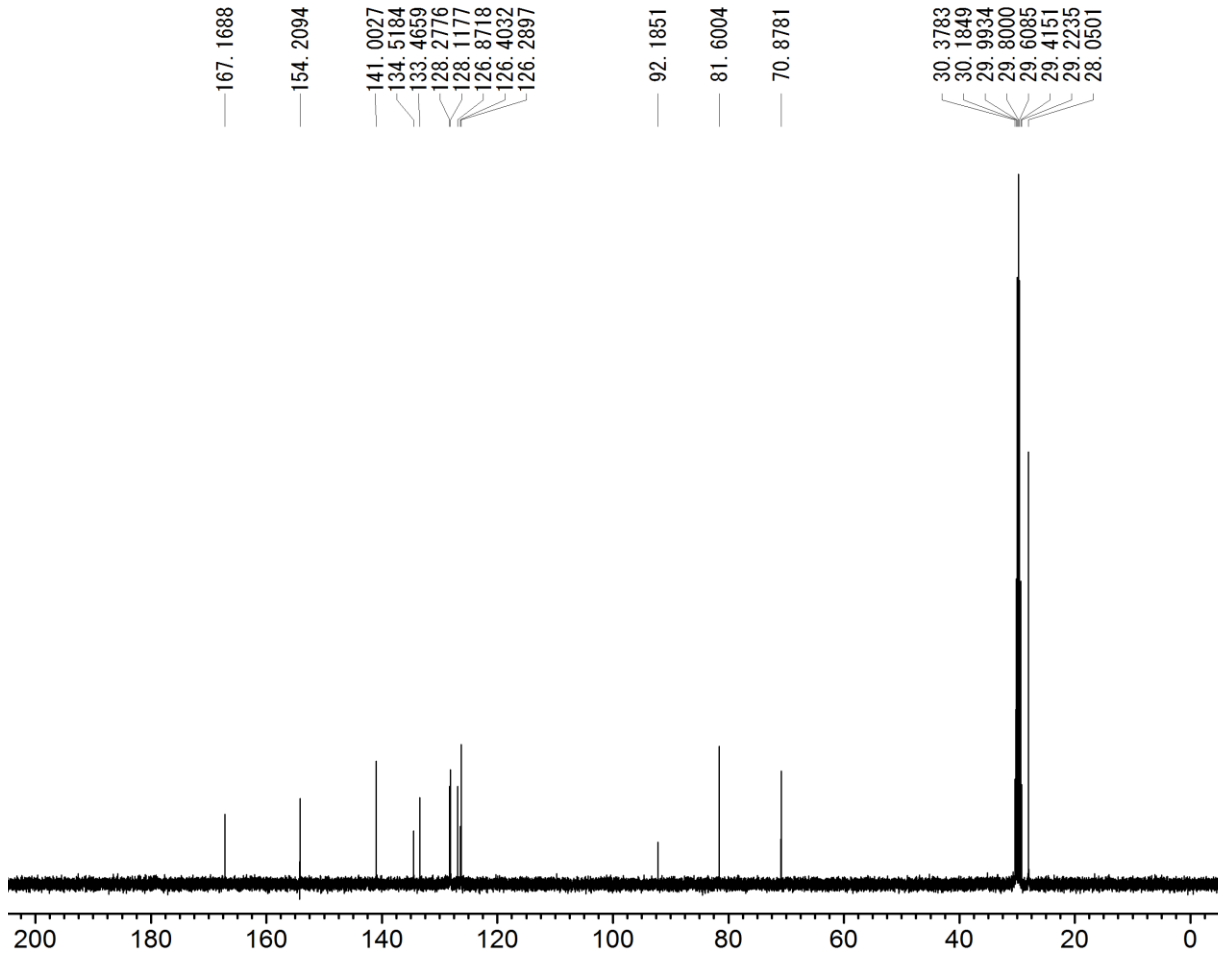

100 MHz ^{13}C NMR spectrum of (*R*)-**3** in d_6-acetone.

Preparation of tetrabutylammonium salts of guests

Acid forms of the analytes were dissolved or dispersed in water. The solutions were titrated with 0.5 M tetrabutylammonium hydroxide (TBA-OH). After addition of 1: 1 molar ratio of TBA-OH and acid forms of analytes, water was thus removed by lyophilization.

Photophysical properties of S1-S4

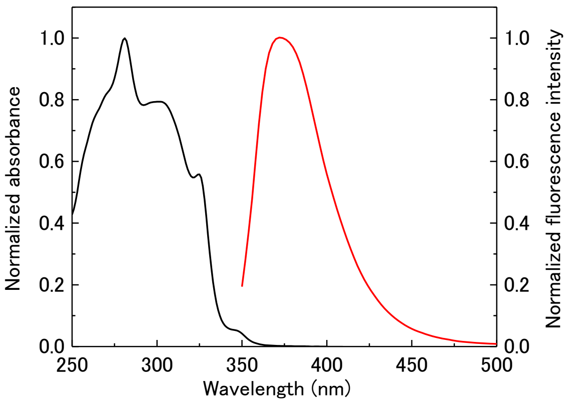

UV-vis absorption and fluorescence spectra of **S1** in propionitrile.

Φ = 3.5%; τ = 1.88 ns 75.17 %

5.93 ns 24.83 % X^2 = 1.01

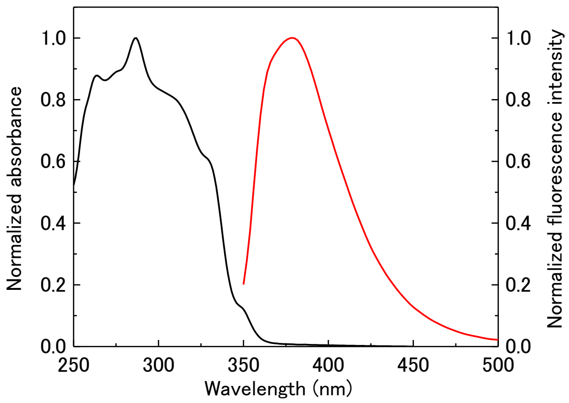

UV-vis absorption and fluorescence spectra of **S2** in propionitrile.

Φ = 3.2%; τ = 0.67 ns 84.53 %

5.17 ns 15.47 % X^2 = 0.99

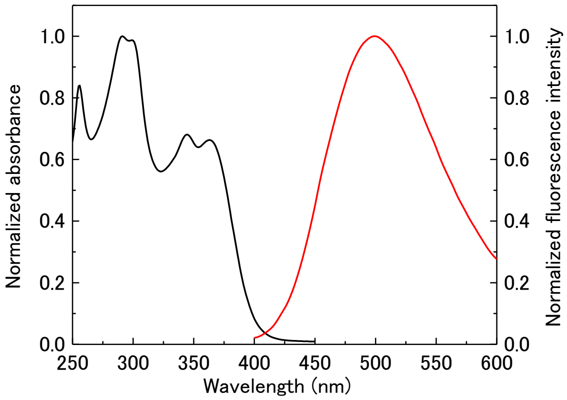

UV-vis absorption and fluorescence spectra of **S3** in propionitrile.

Φ = 1.0%; τ = 3.17 ns 78.30 %

8.61 ns 21.70 % X^2 = 1.35

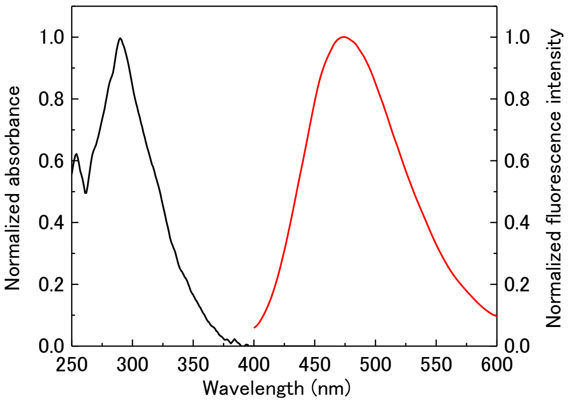

UV-vis absorption and fluorescence spectra of **S4** in propionitrile.

Φ = 0.7%; τ = 2.89 ns 71.93 %

8.22 ns 28.07 % X^2 = 1.24

Fluorescence titrations (S1)

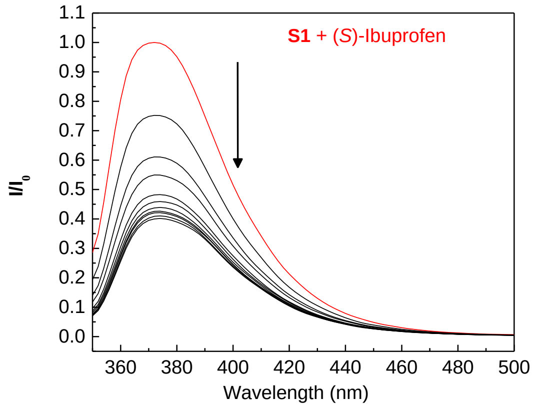

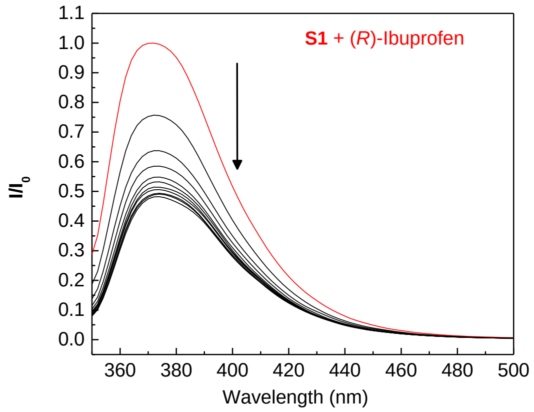

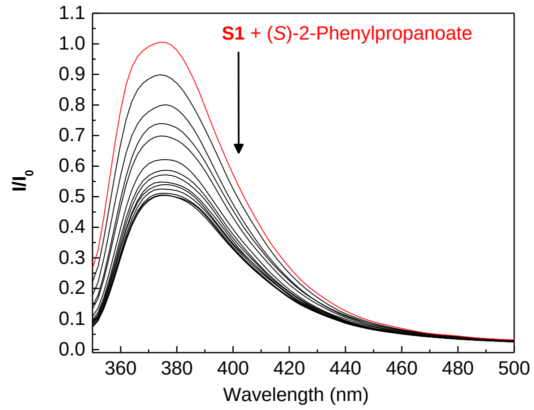

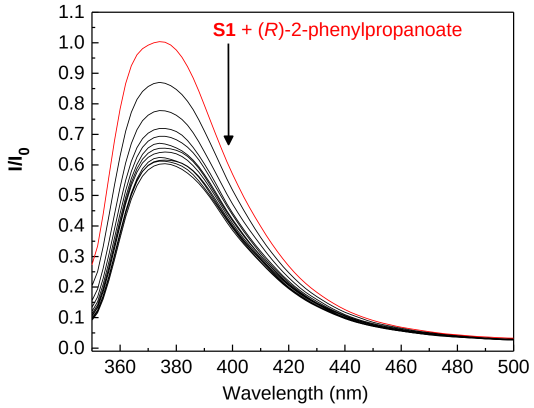

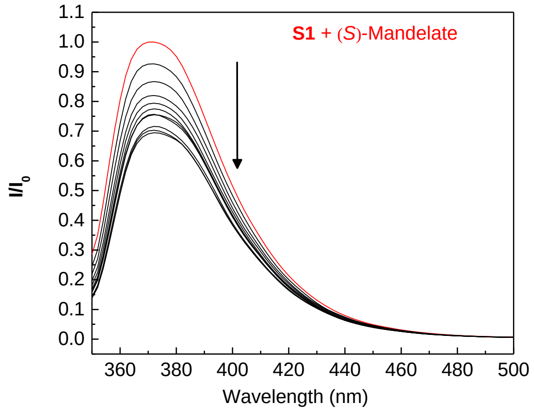

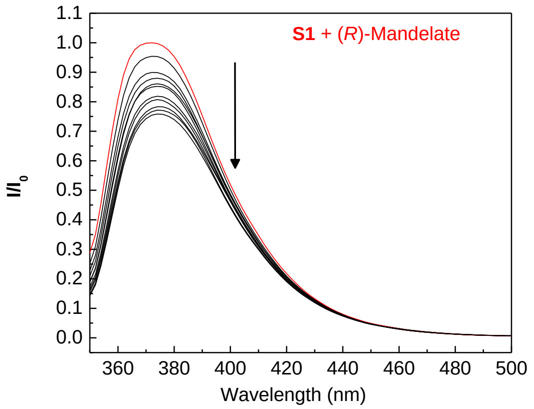

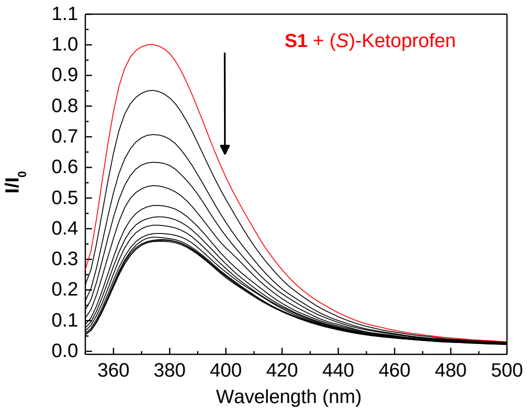
S1 + (S)-Ketoprofen
I/I0
Wavelength (nm)

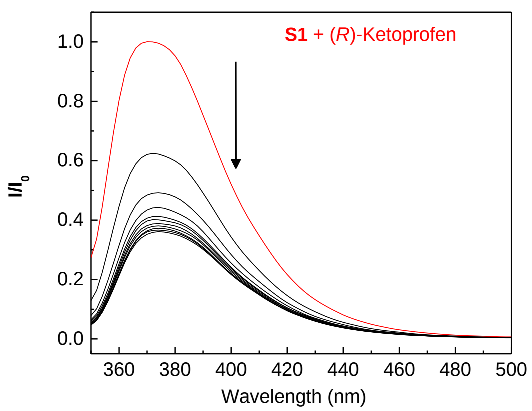
S1 + (R)-Ketoprofen
I/I0
Wavelength (nm)

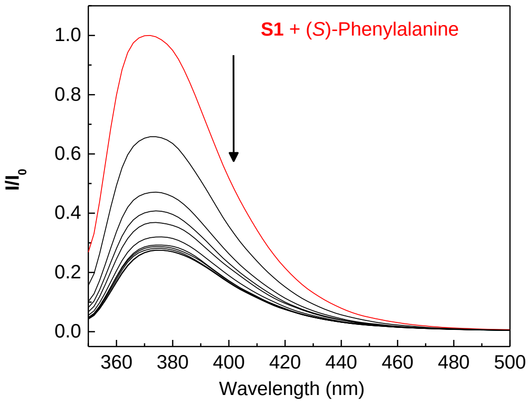
S1 + (S)-Phenylalanine
I/I0
Wavelength (nm)

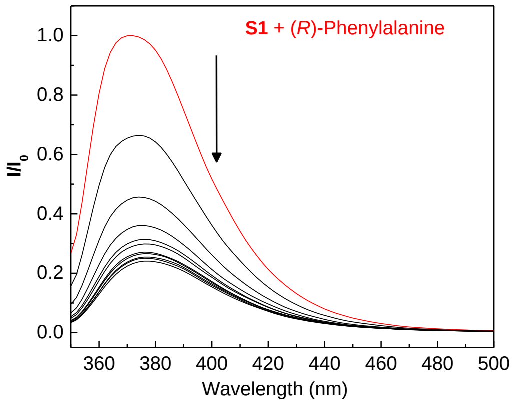
S1 + (R)-Phenylalanine
I/I0
Wavelength (nm)

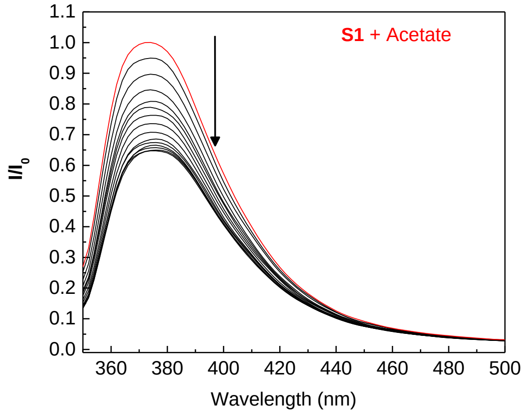
S1 + Acetate
I/I0
Wavelength (nm)

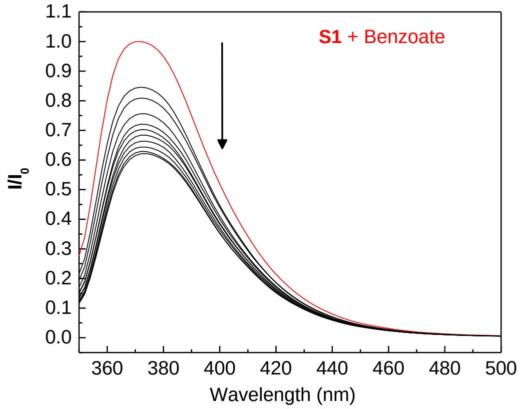
S1 + Benzoate
I/I0
Wavelength (nm)

Binding isotherms (S1)

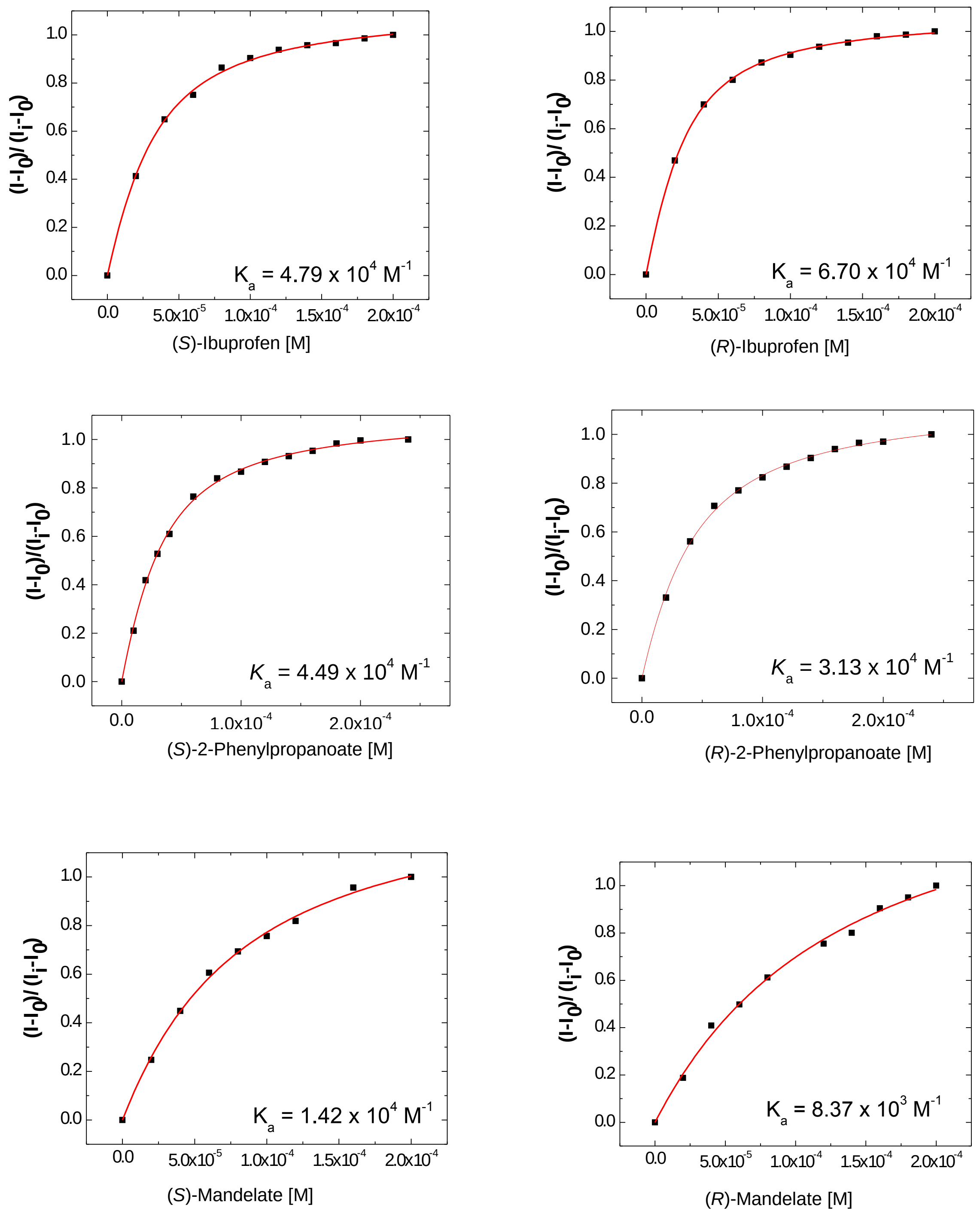

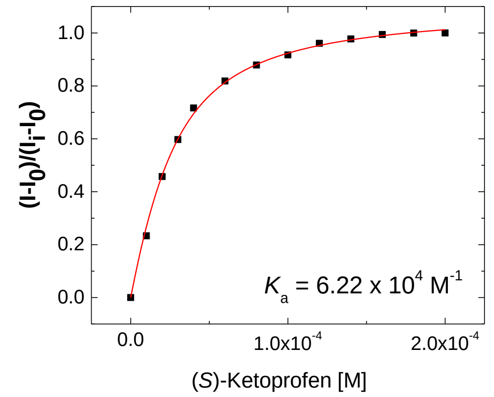
(I-I0)/(Ii-I0)
Ka = 6.22 x 10^4 M^-1
(S)-Ketoprofen [M]

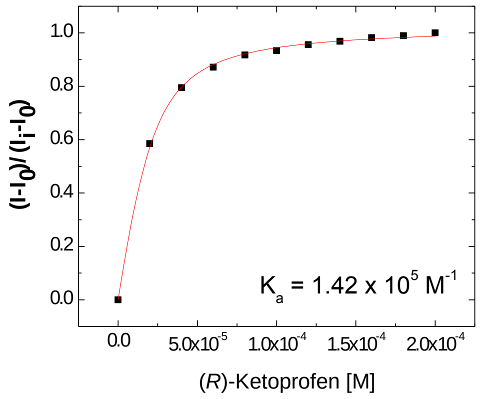
(I-I0)/(Ii-I0)
Ka = 1.42 x 10^5 M^-1
(R)-Ketoprofen [M]

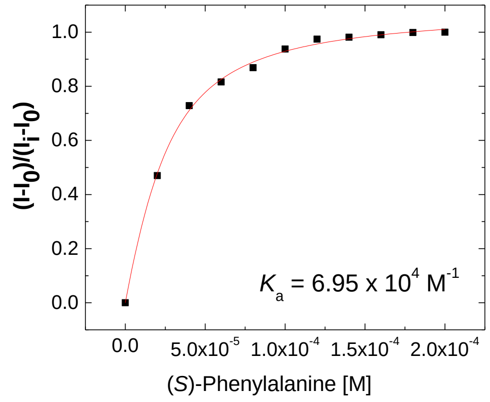
(I-I0)/(Ii-I0)
Ka = 6.95 x 10^4 M^-1
(S)-Phenylalanine [M]

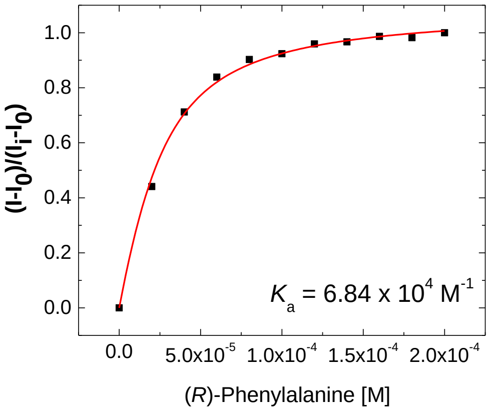
(I-I0)/(Ii-I0)
Ka = 6.84 x 10^4 M^-1
(R)-Phenylalanine [M]

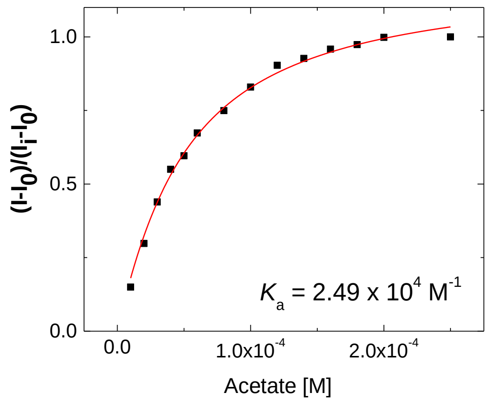
(I-I0)/(Ii-I0)
Ka = 2.49 x 10^4 M^-1
Acetate [M]

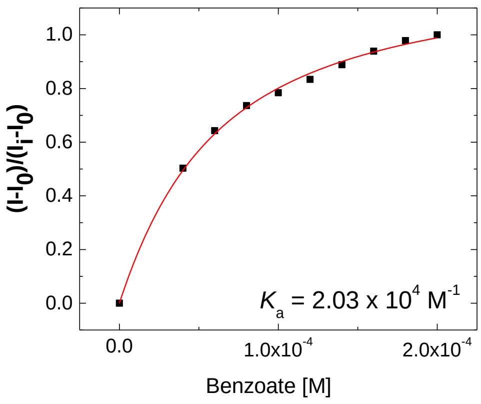
(I-I0)/(Ii-I0)
Ka = 2.03 x 10^4 M^-1
Benzoate [M]

Fluorescence titrations (S2)

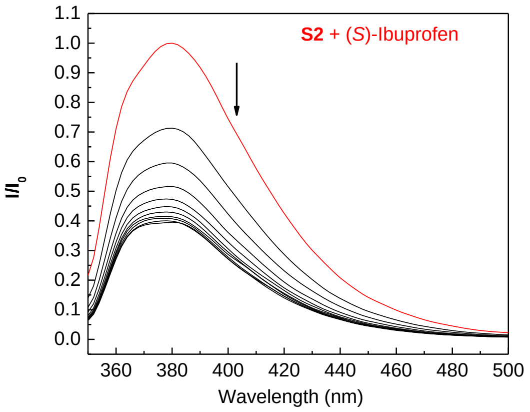

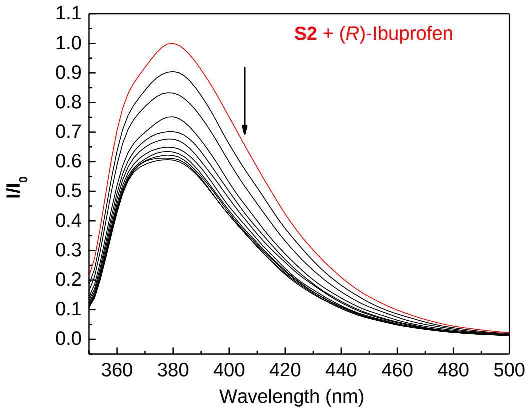

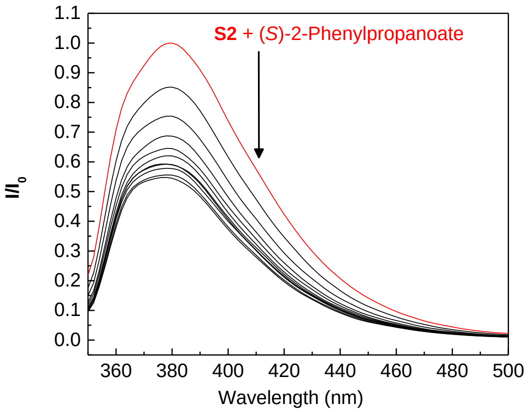

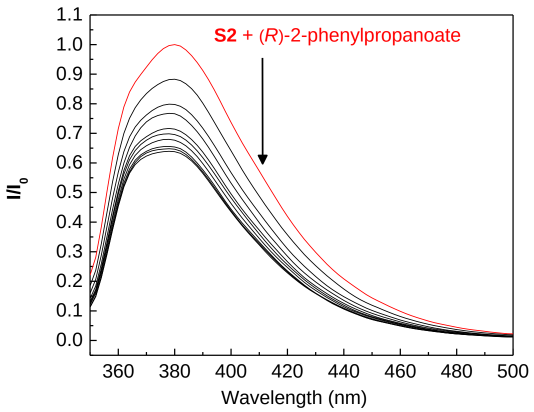

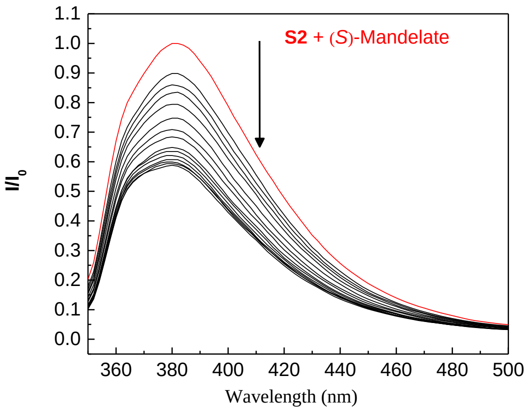

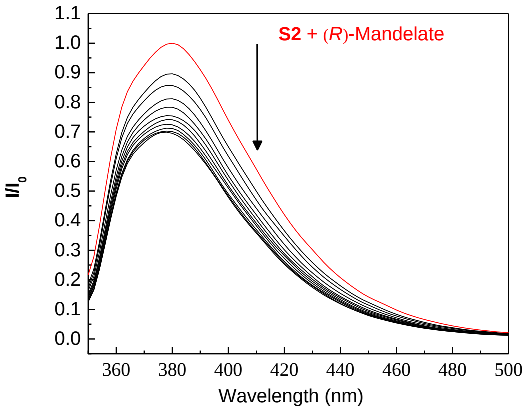

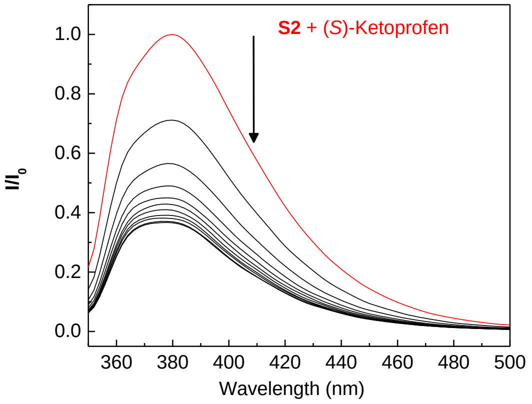
S2 + (S)-Ketoprofen
I/I0
Wavelength (nm)

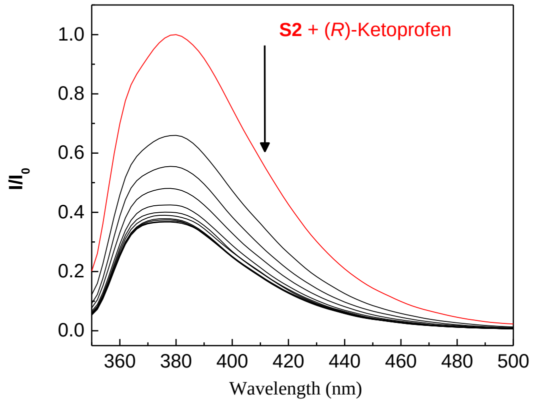
S2 + (R)-Ketoprofen
I/I0
Wavelength (nm)

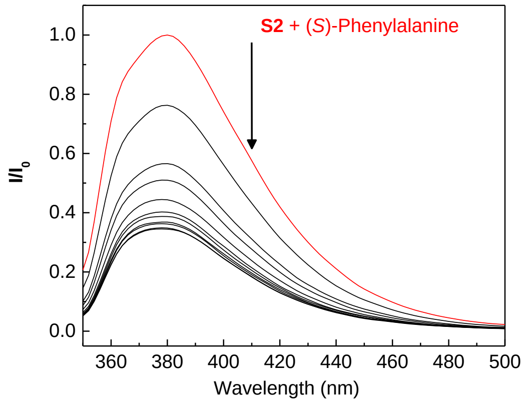
S2 + (S)-Phenylalanine
I/I0
Wavelength (nm)

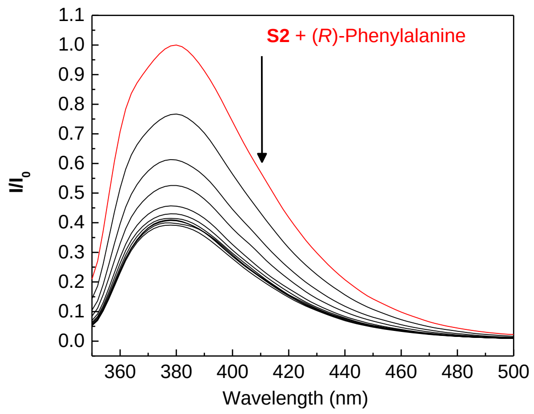
S2 + (R)-Phenylalanine
I/I0
Wavelength (nm)

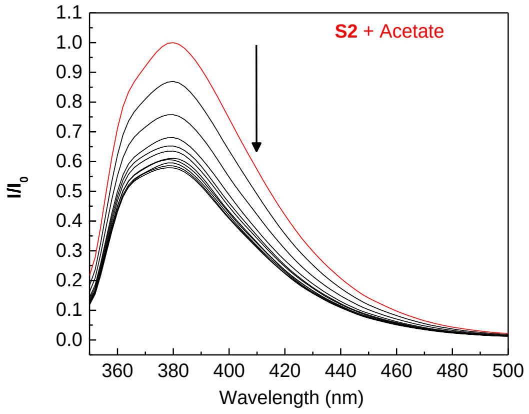
S2 + Acetate
I/I0
Wavelength (nm)

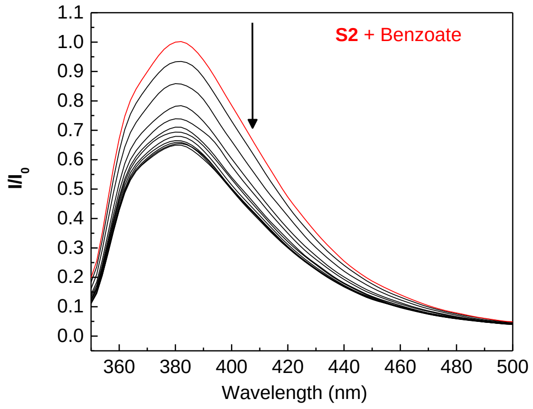
S2 + Benzoate
I/I0
Wavelength (nm)

Binding isotherms (S2)

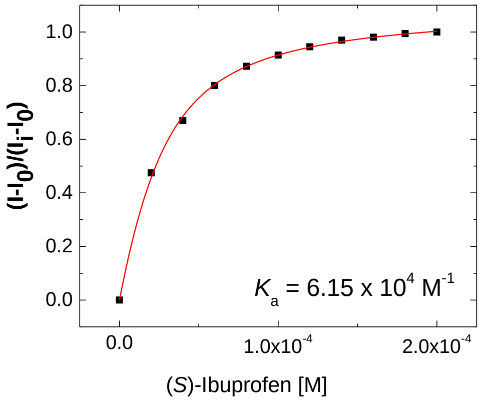

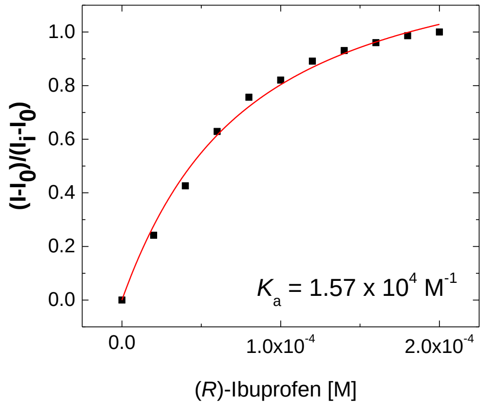

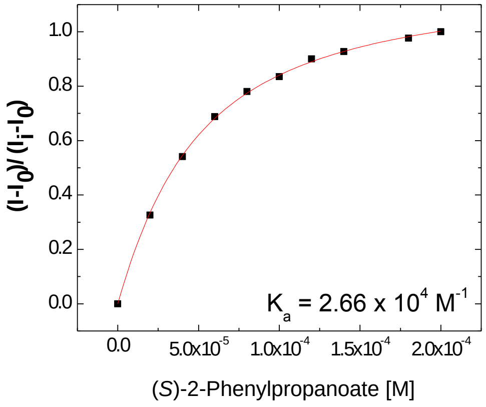

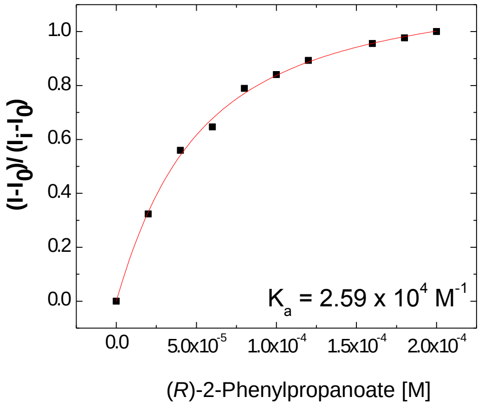

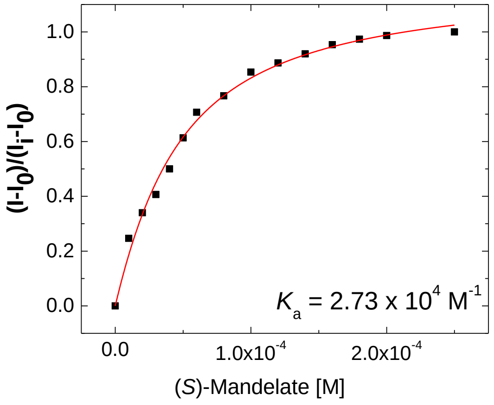

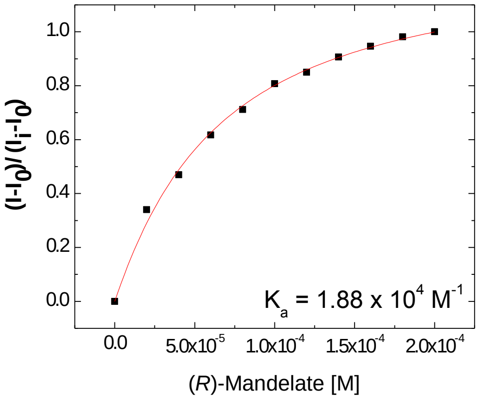

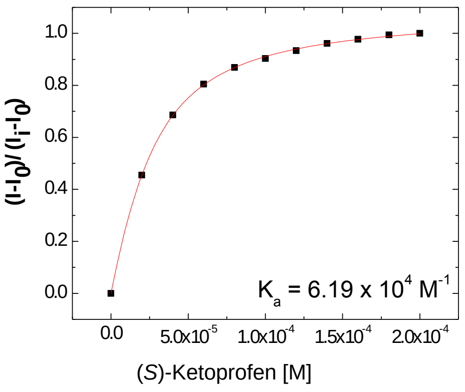
(I-I0)/(Ii-I0)
Ka = 6.19 x 10^4 M^-1
(S)-Ketoprofen [M]

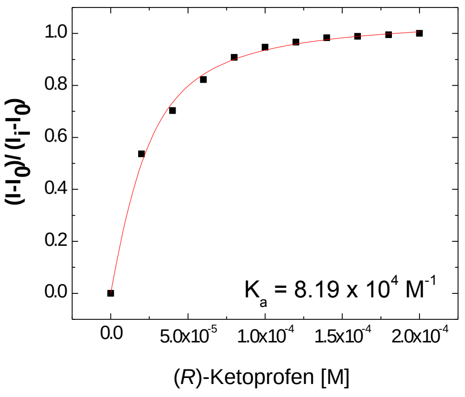
(I-I0)/(Ii-I0)
Ka = 8.19 x 10^4 M^-1
(R)-Ketoprofen [M]

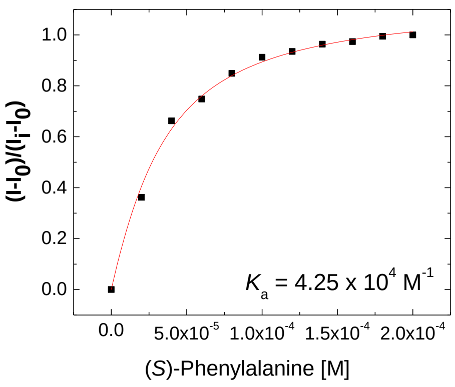
(I-I0)/(Ii-I0)
Ka = 4.25 x 10^4 M^-1
(S)-Phenylalanine [M]

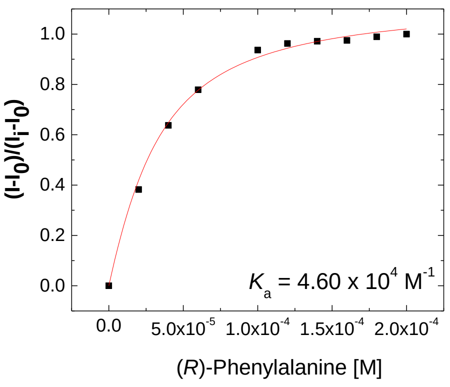
(I-I0)/(Ii-I0)
Ka = 4.60 x 10^4 M^-1
(R)-Phenylalanine [M]

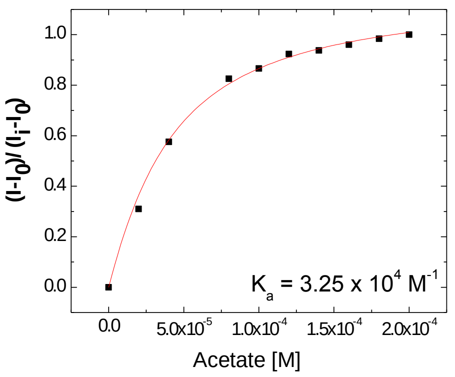
(I-I0)/(Ii-I0)
Ka = 3.25 x 10^4 M^-1
Acetate [M]

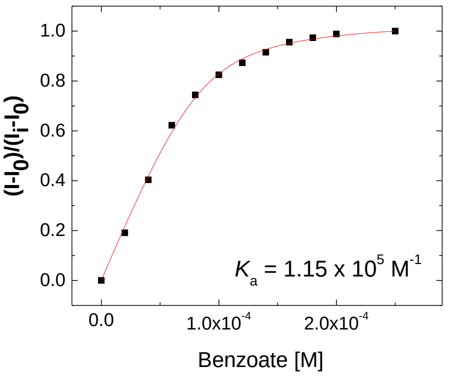
(I-I0)/(Ii-I0)
Ka = 1.15 x 10^5 M^-1
Benzoate [M]

Fluorescence titrations (S3)

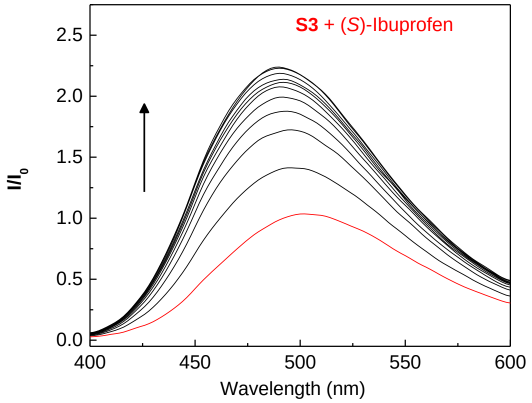

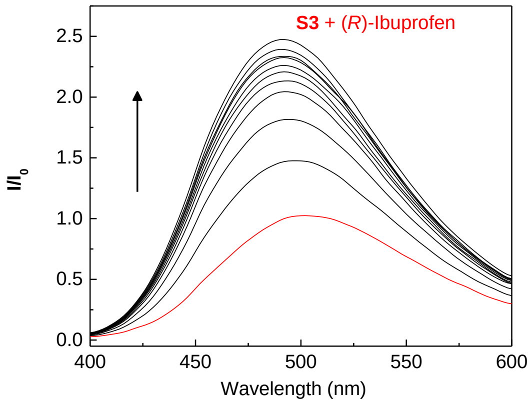

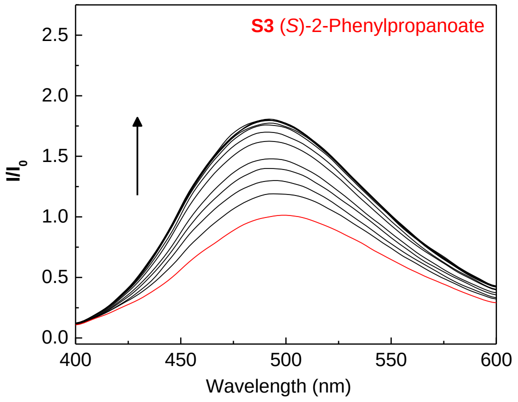

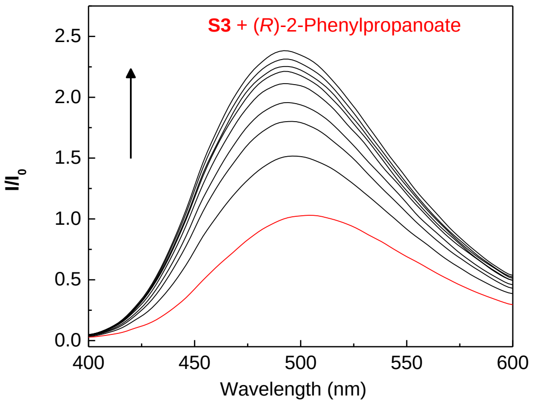

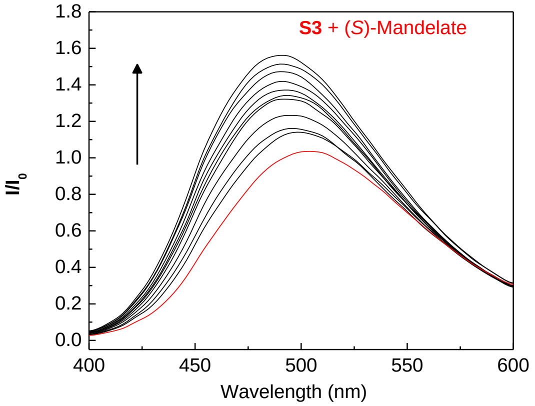

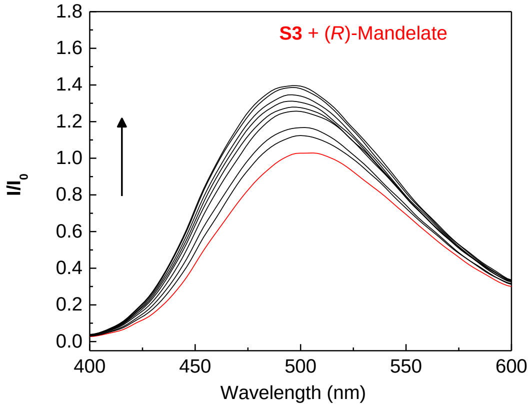

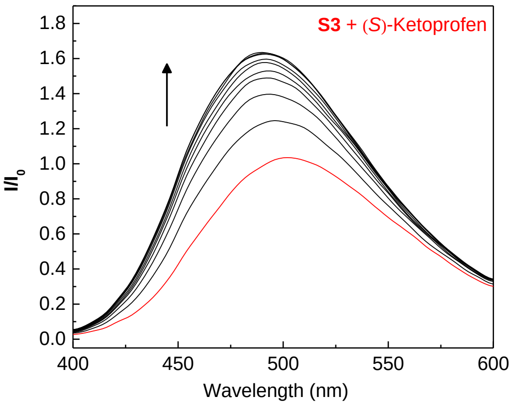
S3 + (S)-Ketoprofen
I/I0
0.0 0.2 0.4 0.6 0.8 1.0 1.2 1.4 1.6 1.8
400 450 500 550 600
Wavelength (nm)

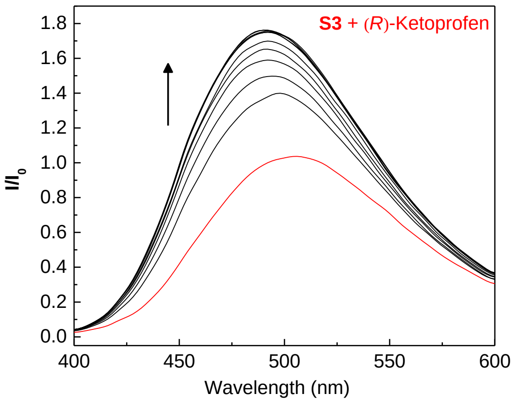
S3 + (R)-Ketoprofen
I/I0
0.0 0.2 0.4 0.6 0.8 1.0 1.2 1.4 1.6 1.8
400 450 500 550 600
Wavelength (nm)

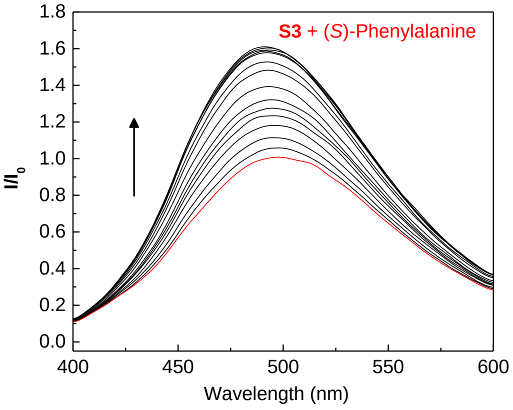
S3 + (S)-Phenylalanine
I/I0
0.0 0.2 0.4 0.6 0.8 1.0 1.2 1.4 1.6 1.8
400 450 500 550 600
Wavelength (nm)

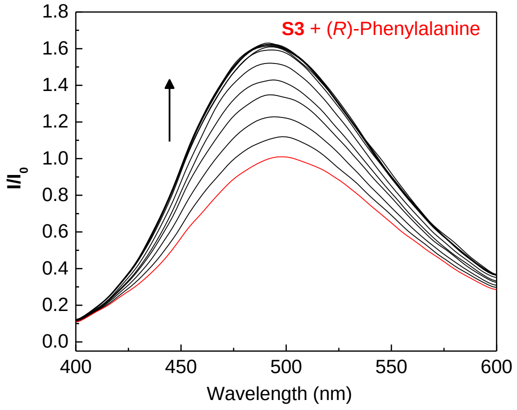
S3 + (R)-Phenylalanine
I/I0
0.0 0.2 0.4 0.6 0.8 1.0 1.2 1.4 1.6 1.8
400 450 500 550 600
Wavelength (nm)

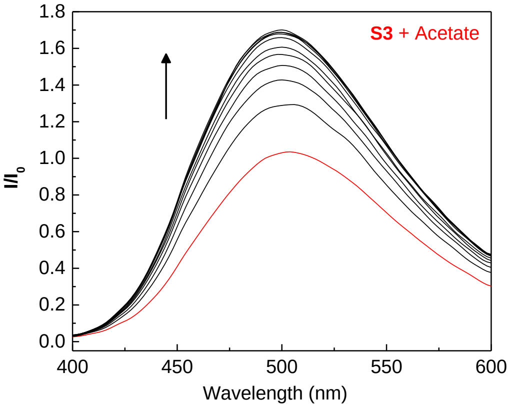
S3 + Acetate
I/I0
0.0 0.2 0.4 0.6 0.8 1.0 1.2 1.4 1.6 1.8
400 450 500 550 600
Wavelength (nm)

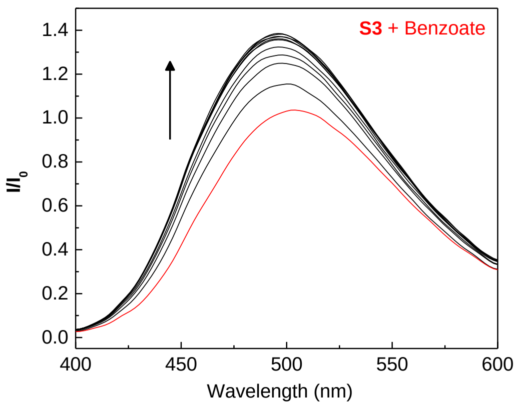
S3 + Benzoate
I/I0
0.0 0.2 0.4 0.6 0.8 1.0 1.2 1.4
400 450 500 550 600
Wavelength (nm)

Binding isotherms (S3)

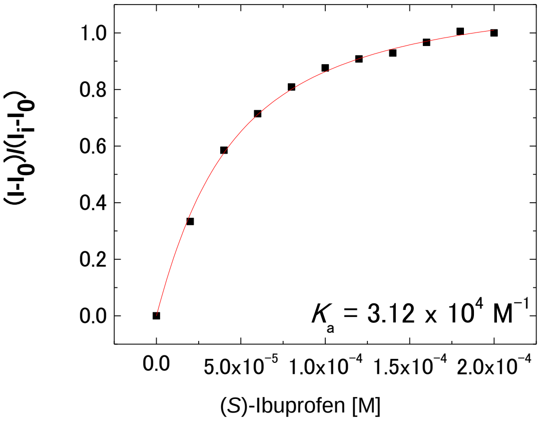

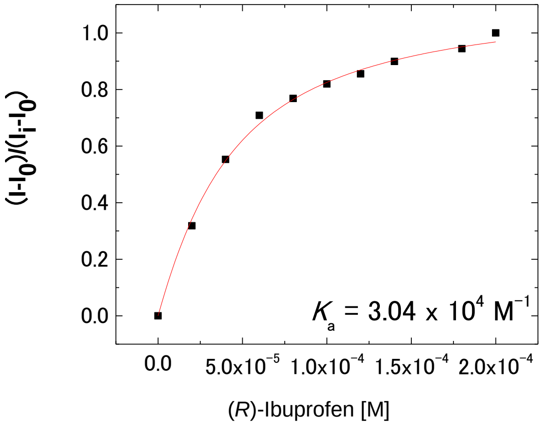

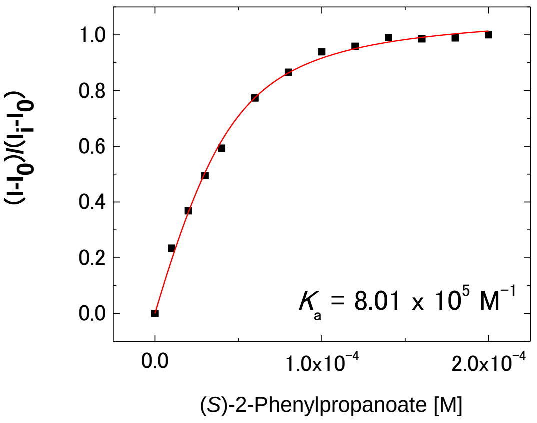

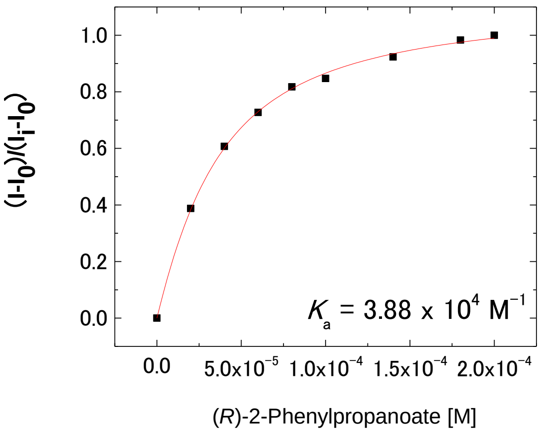

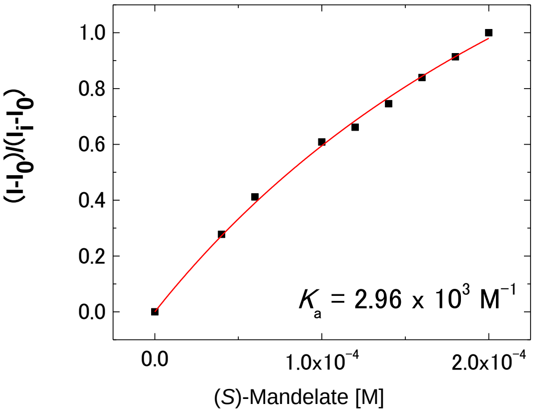

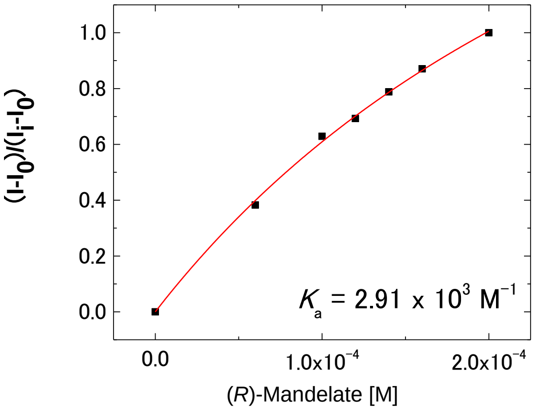

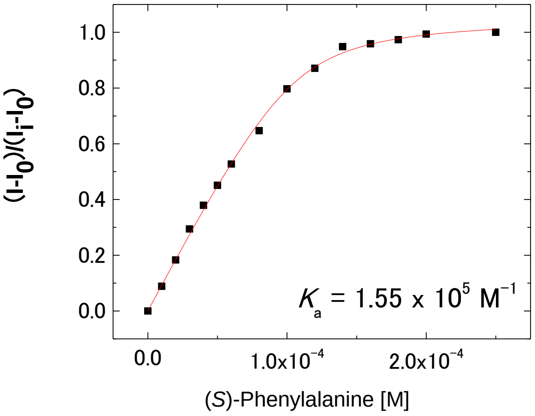
(I-I0)/(Ii-I0)
K_a = 1.55 x 10^5 M^-1
(S)-Phenylalanine [M]

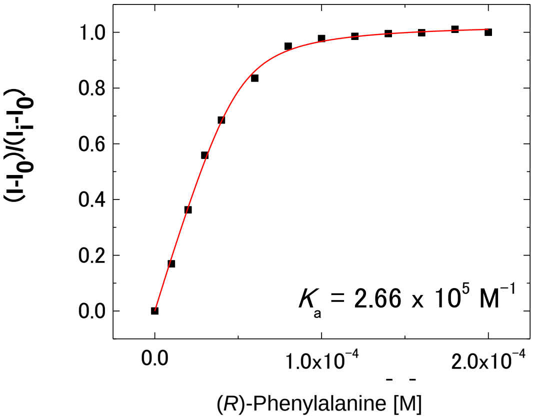
(I-I0)/(Ii-I0)
K_a = 2.66 x 10^5 M^-1
(R)-Phenylalanine [M]

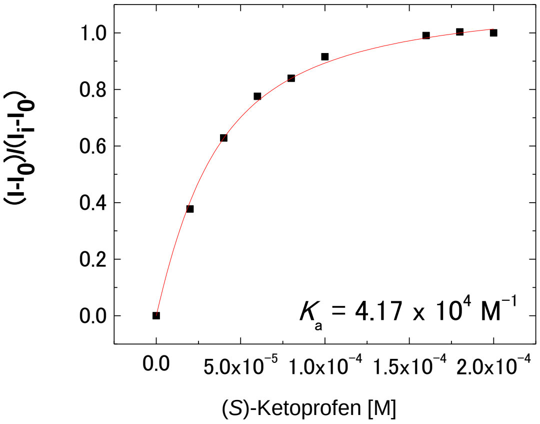
(I-I0)/(Ii-I0)
K_a = 4.17 x 10^4 M^-1
(S)-Ketoprofen [M]

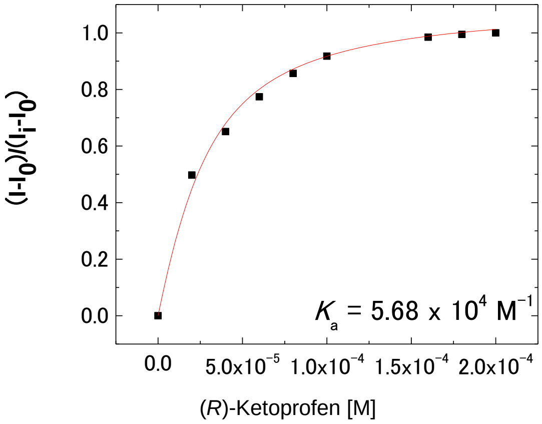
(I-I0)/(Ii-I0)
K_a = 5.68 x 10^4 M^-1
(R)-Ketoprofen [M]

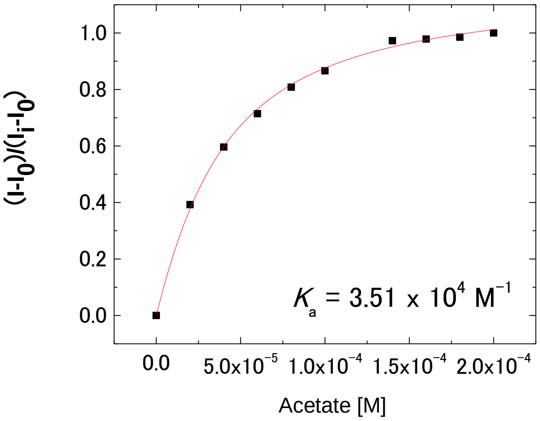
(I-I0)/(Ii-I0)
K_a = 3.51 x 10^4 M^-1
Acetate [M]

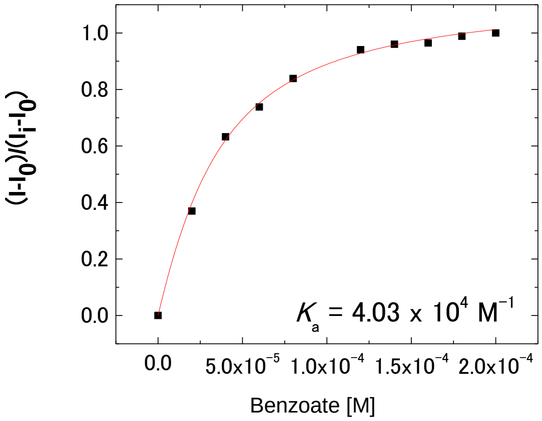
(I-I0)/(Ii-I0)
K_a = 4.03 x 10^4 M^-1
Benzoate [M]

Fluorescence titrations (S4)

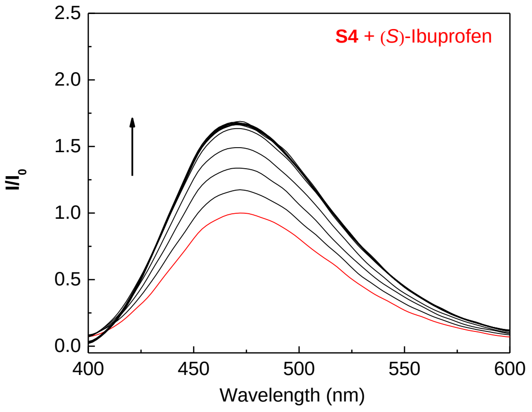

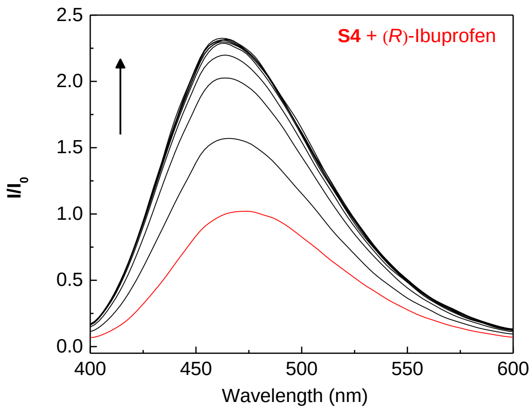

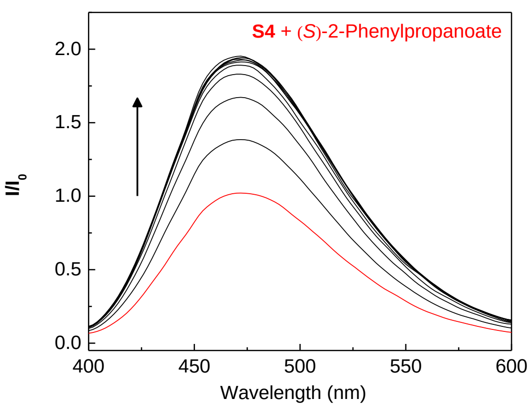

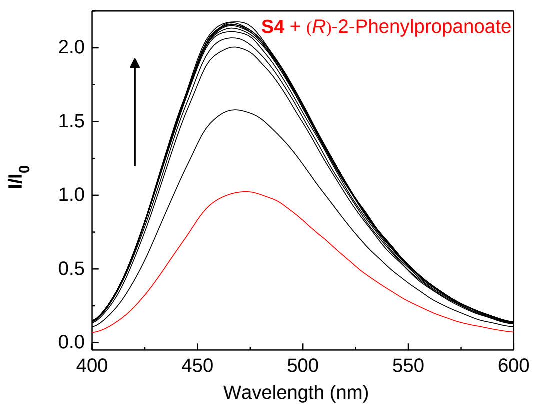

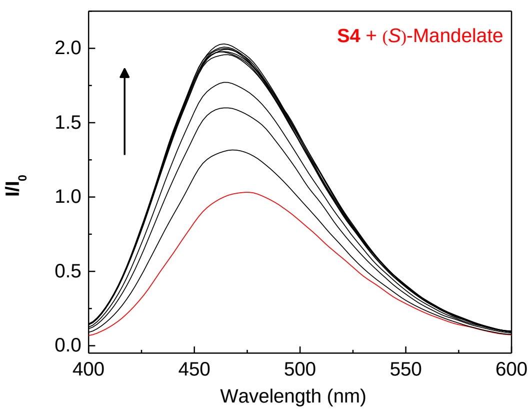

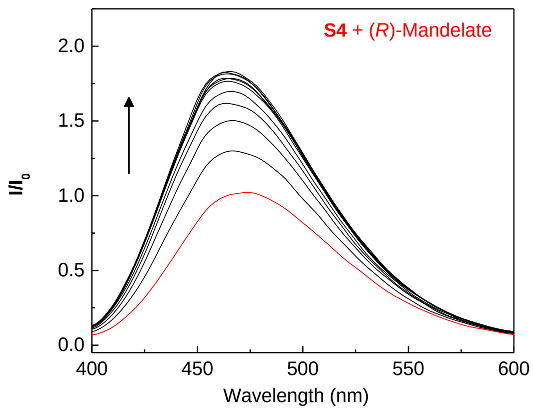

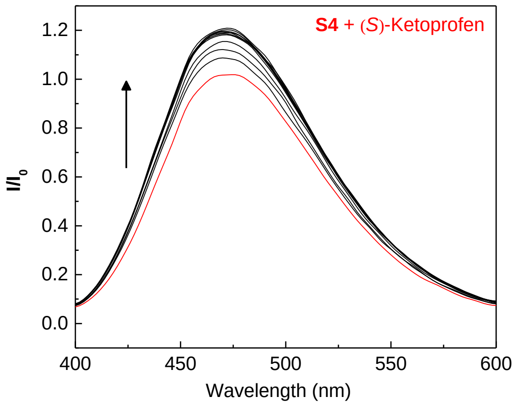
S4 + (S)-Ketoprofen
I/I0
Wavelength (nm)

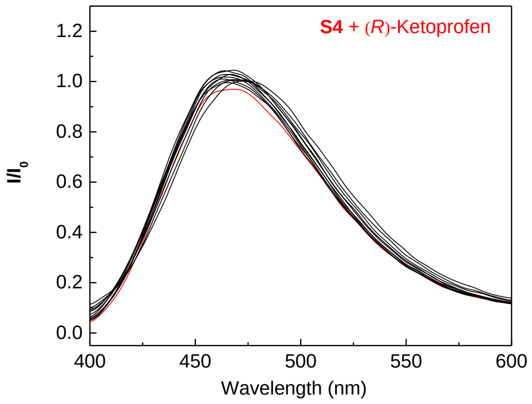
S4 + (R)-Ketoprofen
I/I0
Wavelength (nm)

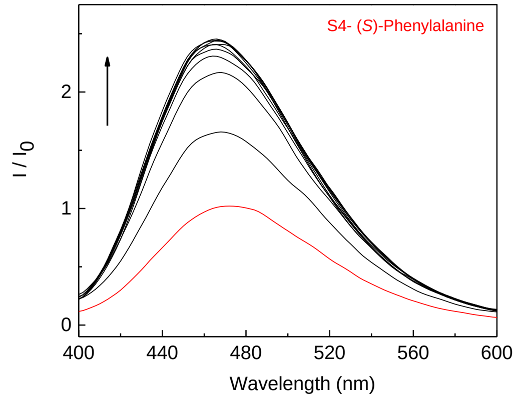
S4- (S)-Phenylalanine
I / I0
Wavelength (nm)

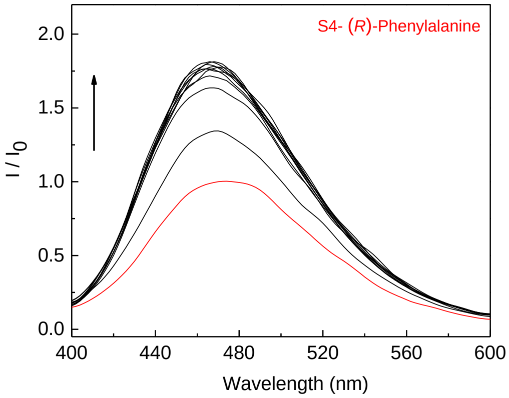
S4- (R)-Phenylalanine
I / I0
Wavelength (nm)

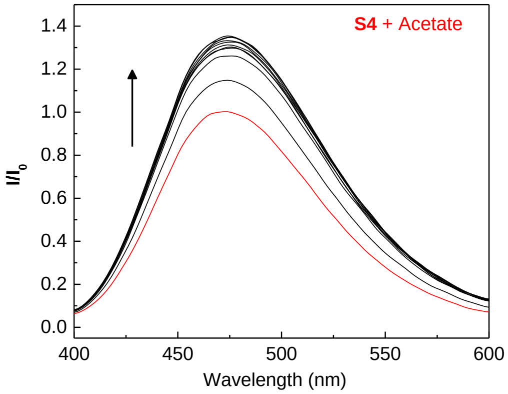
S4 + Acetate
I/I0
Wavelength (nm)

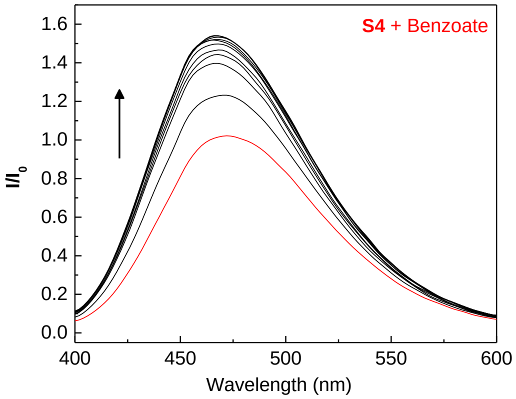
S4 + Benzoate
I/I0
Wavelength (nm)

Binding isotherms (S4)

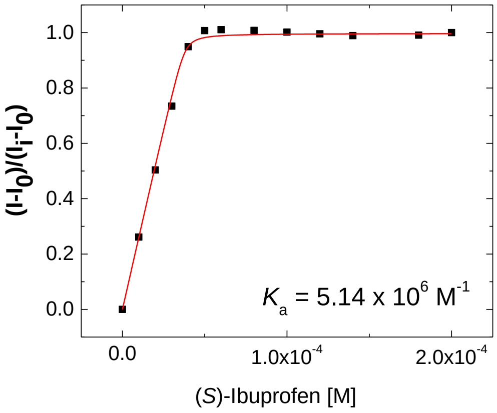

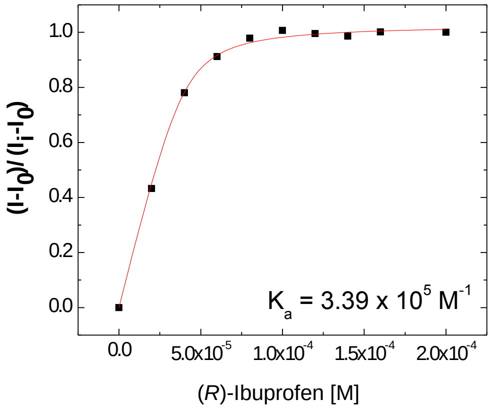

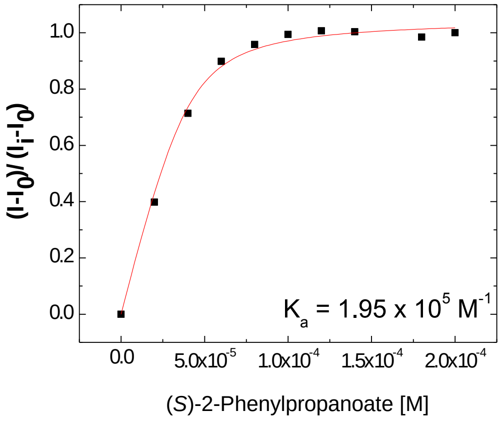

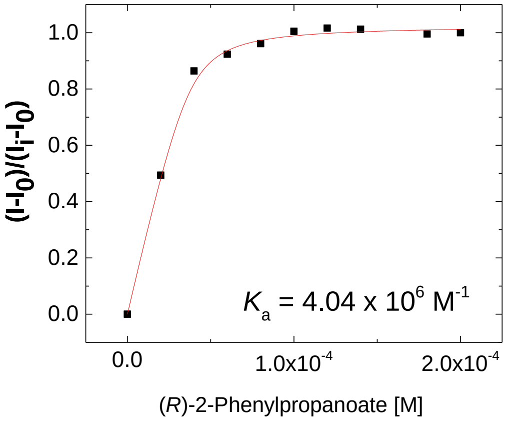

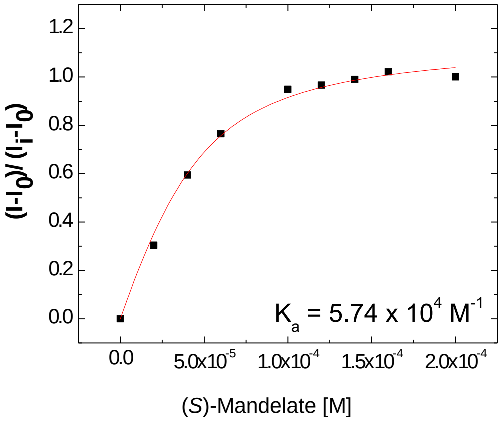

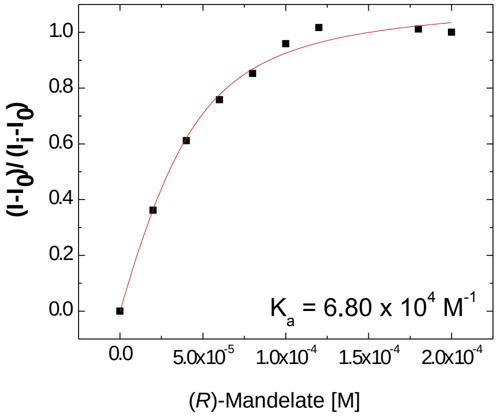

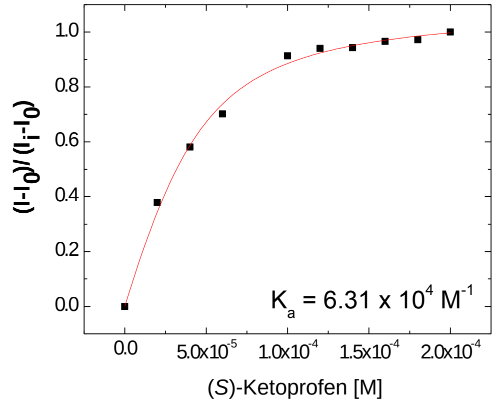
(I-I0)/ (Ii-I0)
Ka = 6.31 x 10^4 M^-1
(S)-Ketoprofen [M]

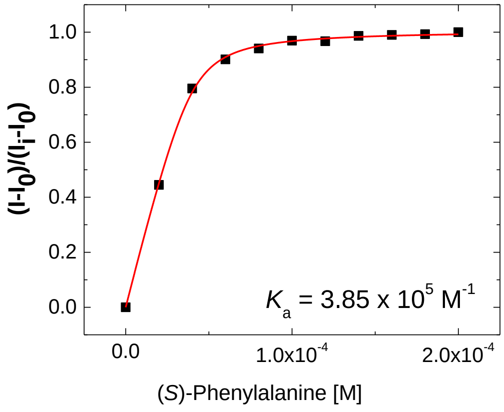
(I-I0)/(Ii-I0)
Ka = 3.85 x 10^5 M^-1
(S)-Phenylalanine [M]

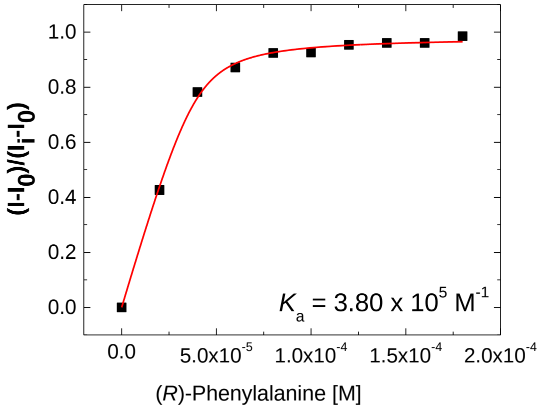
(I-I0)/(Ii-I0)
Ka = 3.80 x 10^5 M^-1
(R)-Phenylalanine [M]

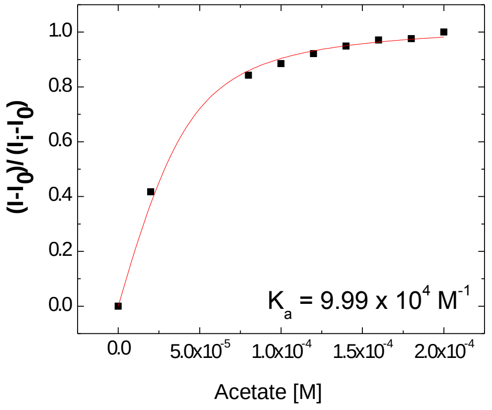
(I-I0)/ (Ii-I0)
Ka = 9.99 x 10^4 M^-1
Acetate [M]

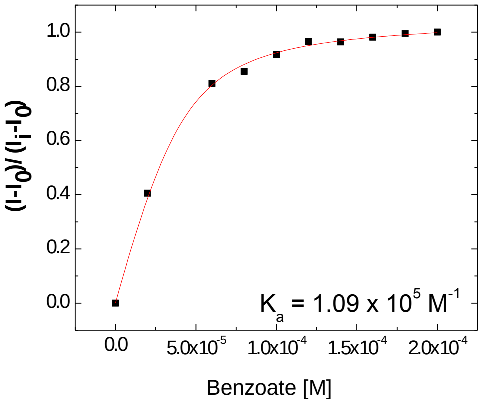
(I-I0)/ (Ii-I0)
Ka = 1.09 x 10^5 M^-1
Benzoate [M]

Association constants with percent errors

Association constants (K_a, M^{-1}) with percent errors.[a]

Guest	**S1**	% Error	**S2**	% Error	**S3**	% Error	**S4**	% Error
(*R*)-Ibuprofen	6.70×10^4	3	1.57×10^4	12	3.04×10^4	9	3.39×10^5	15
(*S*)-Ibuprofen	4.79×10^4	5	6.15×10^4	4	3.12×10^4	6	5.14×10^6	13
(*R*)-Ketoprofen	1.42×10^5	5	8.19×10^4	9	5.68×10^4	12	ND[b]	ND[b]
(*S*)-Ketoprofen	6.22×10^4	5	6.19×10^4	2	4.14×10^4	6	6.31×10^4	13
(*R*)-2-Phenylpropanoate	3.13×10^4	4	2.59×10^4	6	3.88×10^4	5	4.04×10^6	11
(*S*)-2-Phenylpropanoate	4.49×10^4	4	2.66×10^4	3	8.01×10^5	4	1.95×10^5	9
(*R*)-Mandelate	8.37×10^3	12	1.88×10^4	8	2.91×10^3	13	6.80×10^4	7
(*S*)-Mandelate	1.42×10^4	7	2.73×10^4	9	2.96×10^3	15	5.74×10^4	6
(*R*)-Phenylalanine	6.84×10^4	7	4.60×10^4	10	2.66×10^5	6	3.80×10^5	15
(*S*)-Phenylalanine	6.95×10^4	6	4.25×10^4	9	1.55×10^5	5	3.85×10^5	9
Benzoate	2.03×10^4	7	1.15×10^5	5	4.03×10^4	8	1.09×10^5	10
Acetate	2.49×10^4	6	3.25×10^4	11	3.51×10^4	6	9.99×10^4	8

[a]Fluorescence titrations were performed in propionitrile at 22 °C. All guests were added as tetrabutylammonium salts. Association constants were calculated by nonlinear least-squares method. [b]Association constant could not be calculated.

The values of enantiomeric fluorescence difference ratio (*ef*[a]).

	***ef* values**			
**Guest**	**S1**	**S2**	**S3**	**S4**
Ibuprofen	1.15	1.53	1.19*	1.96*
Ketoprofen	1.02	1.00	1.21*	6.66*
Mandelic acid	1.29	1.39	1.40	1.29
Phenylalanine	1.04*	1.06	1.02	1.80*
Phenylpropionic acid	1.25	1.23	1.71*	1.26*

(a) The value of enantiomeric fluorescence ratio is calculated by: $ef = (I_S\text{-}I_0)/(I_R\text{-}I_0)$ and $ef^* = (I_R\text{-}I_0)/(I_S\text{-}I_0)$.[5]

Mass spectrometric study of sensor and guest complexes

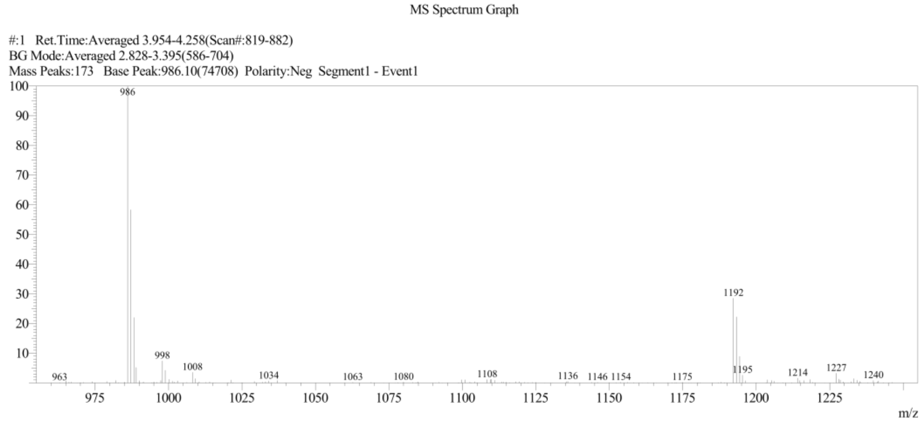

The ESI mass spectrum of the complex of **S1** and ibuprofen.

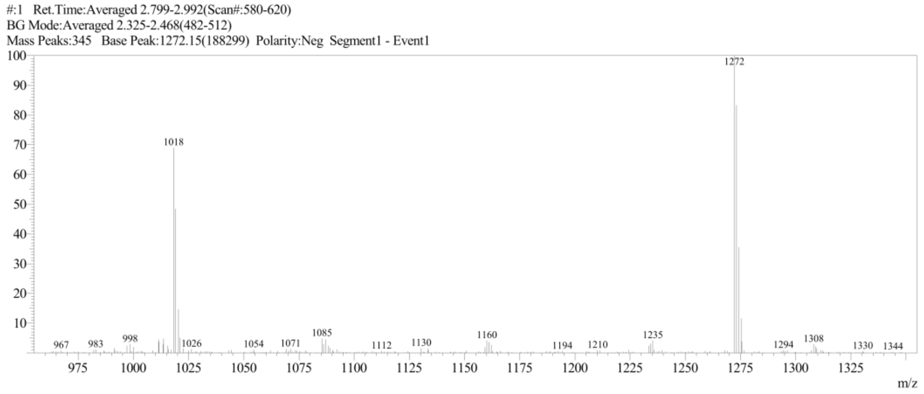

The ESI mass spectrum of the complex of **S2** and ketoprofen.

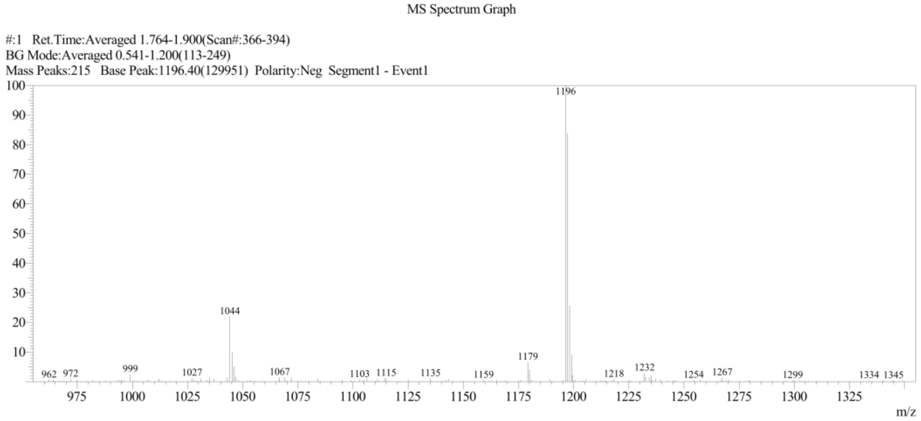

The ESI mass spectrum of the complex of **S3** and mandelate.

Qualitative linear discriminant analysis (LDA)

The jackknifed classification matrix of qualitative assay using **S1**-**S4**.

Jackknifed Classification Matrix

	Acetate	R-Ketoprofen	D-Phenyl Alanine	L-Phenyl Alanine	Control	Benzoate	S-Mandelate	R-Mandelate	S-Ibuprofen	R-Ibuprofen	S-Phenyl Propanoate	R-Phenyl Propanoate	S-Ketoprofen	%correct
Acetate	12	0	0	0	0	0	0	0	0	0	0	0	0	100
R-Ketoprofen	0	12	0	0	0	0	0	0	0	0	0	0	0	100
D-Phenyl Alanine	0	0	12	0	0	0	0	0	0	0	0	0	0	100
L-Phenyl Alanine	0	0	0	12	0	0	0	0	0	0	0	0	0	100
Control	0	0	0	0	12	0	0	0	0	0	0	0	0	100
Benzoate	0	0	0	0	0	12	0	0	0	0	0	0	0	100
S-Mandelate	0	0	0	0	0	0	12	0	0	0	0	0	0	100
R-Mandelate	0	0	0	0	0	0	0	12	0	0	0	0	0	100
S-Ibuprofen	0	0	0	0	0	0	0	0	12	0	0	0	0	100
R-Ibuprofen	0	0	0	0	0	0	0	0	0	12	0	0	0	100
S-Phenyl Propanoate	0	0	0	0	0	0	0	0	0	0	12	0	0	100
R-Phenyl Propanoate	0	0	0	0	0	0	0	0	0	0	0	12	0	100
S-Ketoprofen	0	0	0	0	0	0	0	0	0	0	0	0	12	100
Total	12	12	12	12	12	12	12	12	12	12	12	12	12	100

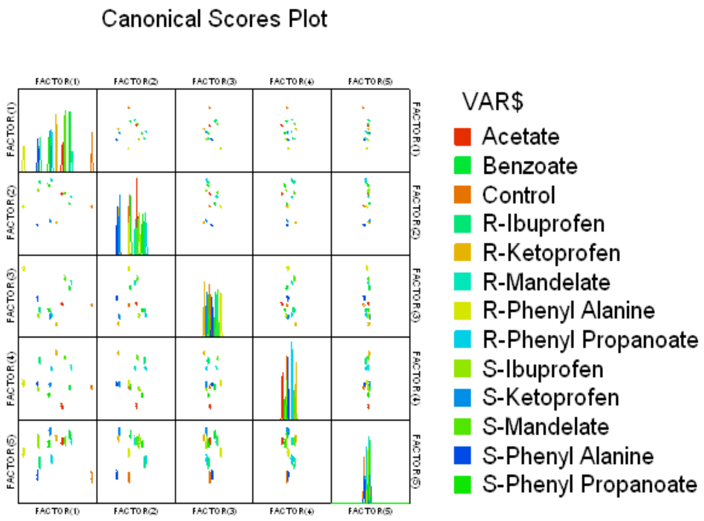

The canonical scores plot of qualitative assay.

Qualitative linear discriminant analysis using only **S4**

The jackknifed classification matrix of qualitative assay.

Jackknifed Classification Matrix	Acetate	R-Ketoprofen	D-Phenyl Alanine	L-Phenyl Alanine	Control	Benzoate	S-Mandelate	R-Mandelate	S-Ibuprofen	R-Ibuprofen	S-Phenyl Propanoate	R-Phenyl Propanoate	S-Ketoprofen	%correct
Acetate	12	0	0	0	0	0	0	0	0	0	0	0	0	100
R-Ketoprofen	0	12	0	0	0	0	0	0	0	0	0	0	0	100
D-Phenyl Alanine	0	0	12	0	0	0	0	0	0	0	0	0	0	100
L-Phenyl Alanine	0	0	0	12	0	0	0	0	0	0	0	0	0	100
Control	0	0	0	0	12	0	0	0	0	0	0	0	0	100
Benzoate	0	0	0	0	0	12	0	0	0	0	0	0	0	100
S-Mandelate	0	0	0	0	0	0	12	0	0	0	0	0	0	100
R-Mandelate	0	0	0	0	0	0	0	12	0	0	0	0	0	100
S-Ibuprofen	0	0	0	0	0	0	0	0	12	0	0	0	0	100
R-Ibuprofen	0	0	0	0	0	0	0	0	0	12	0	0	0	100
S-Phenyl Propanoate	0	0	0	0	0	0	0	0	0	0	12	0	0	100
R-Phenyl Propanoate	0	0	0	0	0	0	0	0	0	0	0	12	0	100
S-Ketoprofen	0	0	0	0	0	0	0	0	0	0	0	0	12	100
Total	12	12	12	12	12	12	12	12	12	12	12	12	12	100

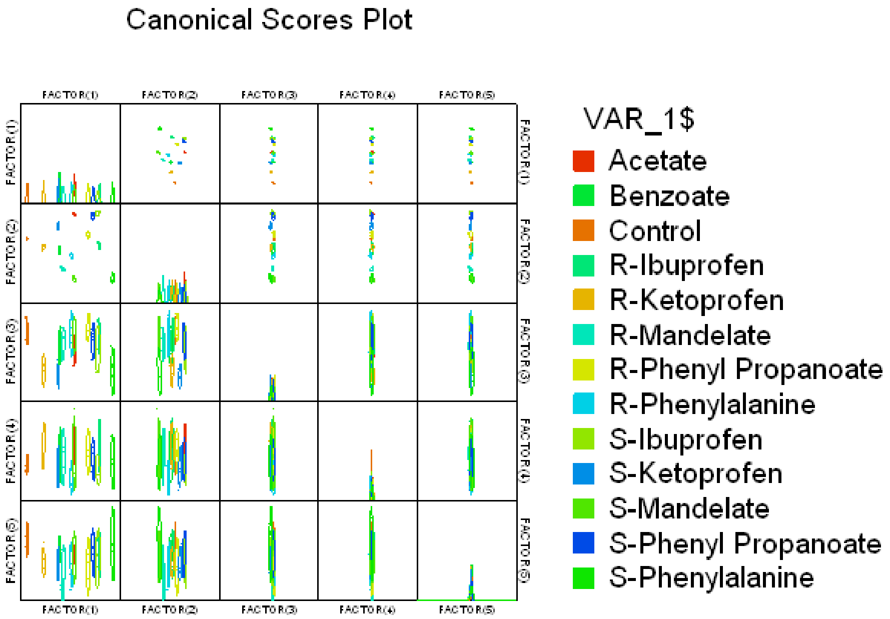

The canonical scores plot of qualitative assay.

Quantitative analysis of phenylalanine

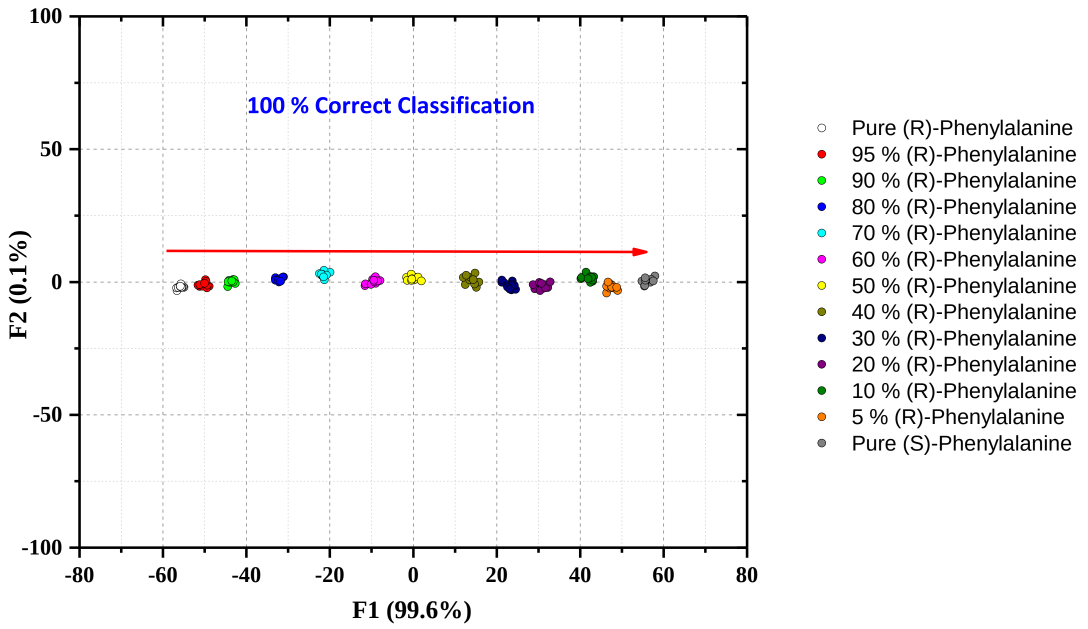

Linear discriminant analysis (LDA) of semi-quantitative assay of enantiomeric composition of phenylalanine using only **S4**.

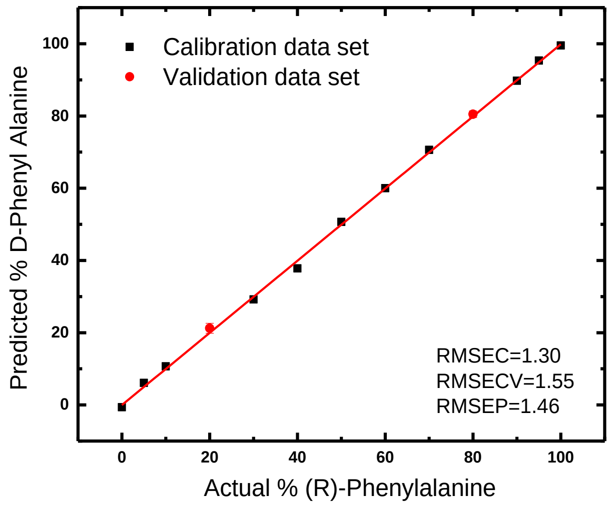

Quantitative analysis of enantiomeric composition of phenylalanine by using SVM with **S4**.

The jackknifed classification matrix of semi-qualitative assay of phenylalanine.

	10 % D-Phenyl Alanine	20 % D-Phenyl Alanine	30 % D-Phenyl Alanine	40 % D-Phenyl Alanine	5 % D-Phenyl Alanine	50 % D-Phenyl Alanine	60 % D-Phenyl Alanine	70 % D-Phenyl Alanine	80 % D-Phenyl Alanine	90 % D-Phenyl Alanine	95 % D-Phenyl Alanine	Pure D-Phenyl Alanine	Pure L-Phenyl Alanine	%correct
10 % D-Phenyl Alanine	12	0	0	0	0	0	0	0	0	0	0	0	0	100
20 % D-Phenyl Alanine	0	12	0	0	0	0	0	0	0	0	0	0	0	100
30 % D-Phenyl Alanine	0	0	12	0	0	0	0	0	0	0	0	0	0	100
40 % D-Phenyl Alanine	0	0	0	12	0	0	0	0	0	0	0	0	0	100
5 % D-Phenyl Alanine	0	0	0	0	12	0	0	0	0	0	0	0	0	100
50 % D-Phenyl Alanine	0	0	0	0	0	12	0	0	0	0	0	0	0	100
60 % D-Phenyl Alanine	0	0	0	0	0	0	12	0	0	0	0	0	0	100
70 % D-Phenyl Alanine	0	0	0	0	0	0	0	12	0	0	0	0	0	100
80 % D-Phenyl Alanine	0	0	0	0	0	0	0	0	12	0	0	0	0	100
90 % D-Phenyl Alanine	0	0	0	0	0	0	0	0	0	12	0	0	0	100
95 % D-Phenyl Alanine	0	0	0	0	0	0	0	0	0	0	12	0	0	100
Pure D-Phenyl Alanine	0	0	0	0	0	0	0	0	0	0	0	12	0	100
Pure L-Phenyl Alanine	0	0	0	0	0	0	0	0	0	0	0	0	12	100
Total	12	12	12	12	12	12	12	12	12	12	12	12	12	100

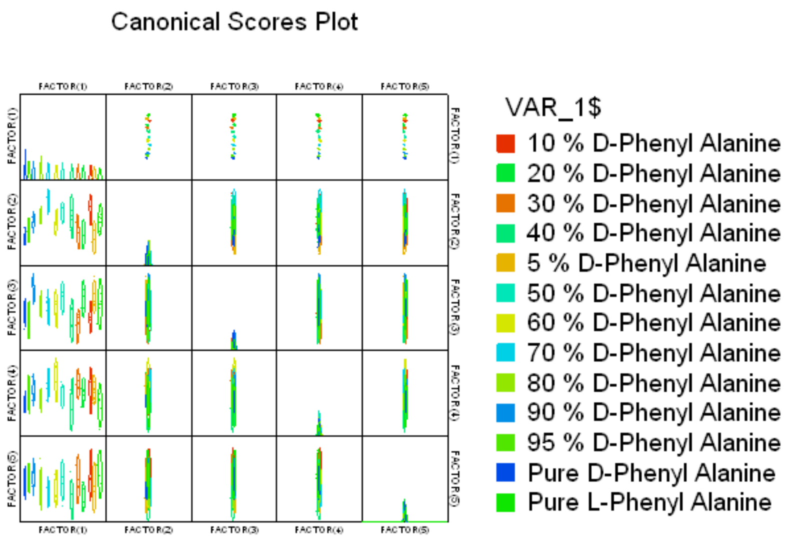

The canonical scores plot of qualitative assay of phenylalanine.

Quantitative analysis of ibuprofen

The jackknifed classification matrix of semi-qualitative assay of ibuprofen using **S1-S4**.

Jackknifed Classification Matrix

	10% S-Ibuprofen	20% S-Ibuprofen	30% S-Ibuprofen	40% S-Ibuprofen	5% S-Ibuprofen	50% S-Ibuprofen
10% S-Ibuprofen	12	0	0	0	0	0
20% S-Ibuprofen	0	12	0	0	0	0
30% S-Ibuprofen	0	0	12	0	0	0
40% S-Ibuprofen	0	0	0	12	0	0
5% S-Ibuprofen	0	0	0	0	12	0
50% S-Ibuprofen	0	0	0	0	0	12
60% S-Ibuprofen	0	0	0	0	0	0
70% S-Ibuprofen	0	0	0	0	0	0
80% S-Ibuprofen	0	0	0	0	0	0
90% S-Ibuprofen	0	0	0	0	0	0
95% S-Ibuprofen	0	0	0	0	0	0
Pure R-Ibuprofen	0	0	0	0	0	0
Pure S-Ibuprofen	0	0	0	0	0	0
Total	12	12	12	12	12	12

Jackknifed Classification Matrix (contd...)

	60% S-Ibuprofen	70% S-Ibuprofen	80% S-Ibuprofen	90% S-Ibuprofen	95% S-Ibuprofen	Pure R-Ibuprofen	Pure S-Ibuprofen	%correct
10% S-Ibuprofen	0	0	0	0	0	0	0	100
20% S-Ibuprofen	0	0	0	0	0	0	0	100
30% S-Ibuprofen	0	0	0	0	0	0	0	100
40% S-Ibuprofen	0	0	0	0	0	0	0	100
5% S-Ibuprofen	0	0	0	0	0	0	0	100
50% S-Ibuprofen	0	0	0	0	0	0	0	100
60% S-Ibuprofen	12	0	0	0	0	0	0	100
70% S-Ibuprofen	0	12	0	0	0	0	0	100
80% S-Ibuprofen	0	0	12	0	0	0	0	100
90% S-Ibuprofen	0	0	0	12	0	0	0	100
95% S-Ibuprofen	0	0	0	0	12	0	0	100
Pure R-Ibuprofen	0	0	0	0	0	12	0	100
Pure S-Ibuprofen	0	0	0	0	0	0	12	100
Total	12	12	12	12	12	12	12	100

Canonical Scores Plot

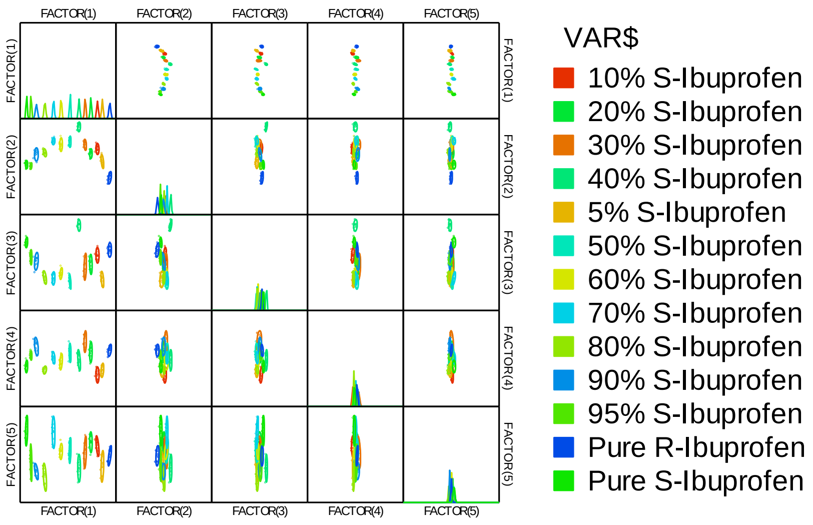

The canonical scores plot of semi-quantitative assay of ibuprofen.

Quantitative analysis of ibuprofen with impurities

Impurity: 5 % Acetate

The jackknifed classification matrix of semi-qualitative assay in the presence of 5 % acetate using **S1-S4**.

Jackknifed Classification Matrix

	10 % S-Ibuprofen	20 % S-Ibuprofen	30 % S-Ibuprofen	40 % S-Ibuprofen	5 % S-Ibuprofen	50 % S-Ibuprofen	60 % S-Ibuprofen	70 % S-Ibuprofen	80 % S-Ibuprofen	90 % S-Ibuprofen	95 % S-Ibuprofen	Pure R-Ibuprofen	Pure S-ibuprofen	%correct
10 % S-Ibuprofen	12	0	0	0	0	0	0	0	0	0	0	0	0	100
20 % S-Ibuprofen	0	12	0	0	0	0	0	0	0	0	0	0	0	100
30 % S-Ibuprofen	0	0	12	0	0	0	0	0	0	0	0	0	0	100
40 % S-Ibuprofen	0	0	0	12	0	0	0	0	0	0	0	0	0	100
5 % S-Ibuprofen	0	0	0	0	12	0	0	0	0	0	0	0	0	100
50 % S-Ibuprofen	0	0	0	0	0	12	0	0	0	0	0	0	0	100
60 % S-Ibuprofen	0	0	0	0	0	0	12	0	0	0	0	0	0	100
70 % S-Ibuprofen	0	0	0	0	0	0	0	12	0	0	0	0	0	100
80 % S-Ibuprofen	0	0	0	0	0	0	0	0	12	0	0	0	0	100
90 % S-Ibuprofen	0	0	0	0	0	0	0	0	0	12	0	0	0	100
95 % S-Ibuprofen	0	0	0	0	0	0	0	0	0	0	12	0	0	100
Pure R-Ibuprofen	0	0	0	0	0	0	0	0	0	0	0	12	0	100
Pure S-ibuprofen	0	0	0	0	0	0	0	0	0	0	0	0	12	100
Total	12	12	12	12	12	12	12	12	12	12	12	12	12	100

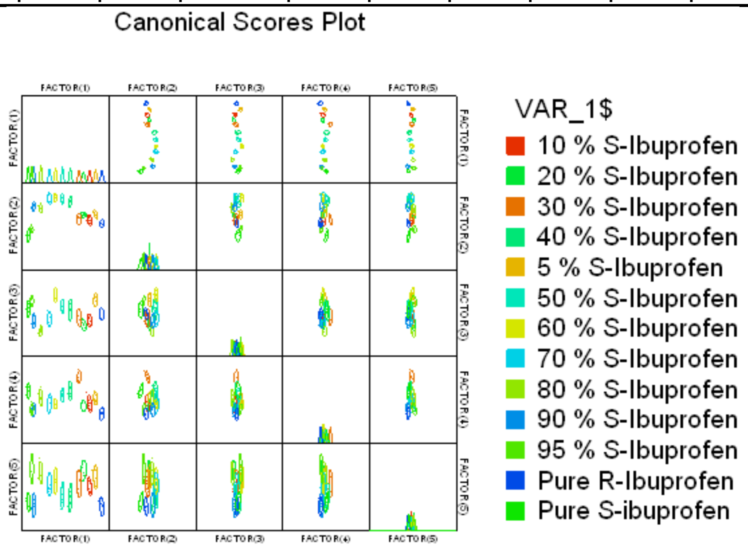

The canonical scores plot of semi-quantitative assay of ibuprofen in the presence of 5 % acetate.

Impurity: 5 % Pyrophosphate

The jackknifed classification matrix of semi-qualitative assay in the presence of 5 % pyrophosphate using **S1-S4**.

Jackknifed Classification Matrix

	10 % S-Ibuprofen	20 % S-Ibuprofen	30 % S-Ibuprofen	40 % S-Ibuprofen	5 % S-Ibuprofen	50 % S-Ibuprofen	60 % S-Ibuprofen	70 % S-Ibuprofen	80 % S-Ibuprofen	90 % S-Ibuprofen	95 % S-Ibuprofen	Pure R-Ibuprofen	Pure S-ibuprofen	%correct
10 % S-Ibuprofen	12	0	0	0	0	0	0	0	0	0	0	0	0	100
20 % S-Ibuprofen	0	12	0	0	0	0	0	0	0	0	0	0	0	100
30 % S-Ibuprofen	0	0	12	0	0	0	0	0	0	0	0	0	0	100
40 % S-Ibuprofen	0	0	0	12	0	0	0	0	0	0	0	0	0	100
5 % S-Ibuprofen	0	0	0	0	12	0	0	0	0	0	0	0	0	100
50 % S-Ibuprofen	0	0	0	0	0	12	0	0	0	0	0	0	0	100
60 % S-Ibuprofen	0	0	0	0	0	0	12	0	0	0	0	0	0	100
70 % S-Ibuprofen	0	0	0	0	0	0	0	12	0	0	0	0	0	100
80 % S-Ibuprofen	0	0	0	0	0	0	0	0	12	0	0	0	0	100
90 % S-Ibuprofen	0	0	0	0	0	0	0	0	0	12	0	0	0	100
95 % S-Ibuprofen	0	0	0	0	0	0	0	0	0	0	12	0	0	100
Pure R-Ibuprofen	0	0	0	0	0	0	0	0	0	0	0	12	0	100
Pure S-ibuprofen	0	0	0	0	0	0	0	0	0	0	0	0	12	100
Total	12	12	12	12	12	12	12	12	12	12	12	12	12	100

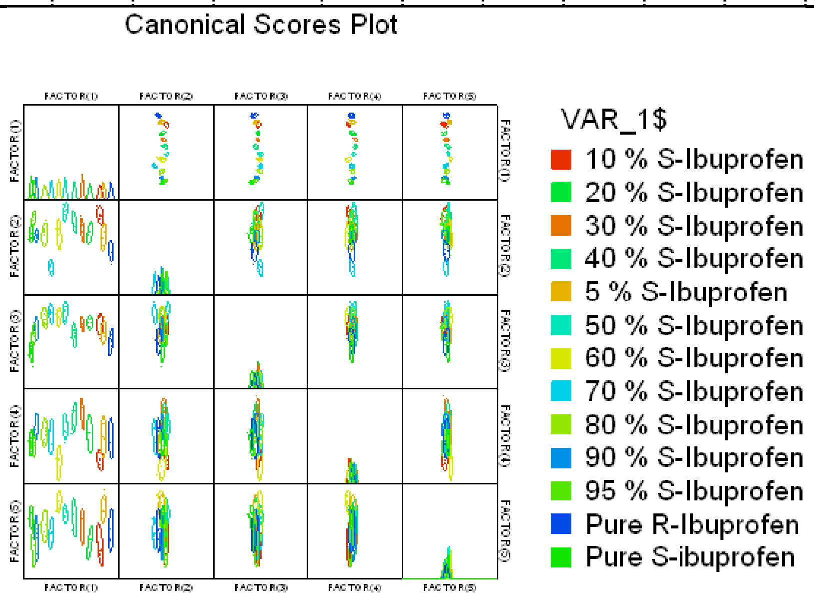

The canonical scores plot of semi-quantitative assay of ibuprofen in the presence of 5 % pyrophosphate.

Impurity: 0 to 10 % Acetate

The jackknifed classification matrix of semi-qualitative assay in the presence of 0 to 10 % increasing concentration of acetate using **S1-S4**.

Jackknifed Classification Matrix

	10 % S-Ibuprofen	20 % S-Ibuprofen	30 % S-Ibuprofen	40 % S-Ibuprofen	5 % S-Ibuprofen	50 % S-Ibuprofen	60 % S-Ibuprofen	70 % S-Ibuprofen	80 % S-Ibuprofen	90 % S-Ibuprofen	95 % S-Ibuprofen	Pure R-Ibuprofen	Pure S-ibuprofen	%correct
10 % S-Ibuprofen	12	0	0	0	0	0	0	0	0	0	0	0	0	100
20 % S-Ibuprofen	0	12	0	0	0	0	0	0	0	0	0	0	0	100
30 % S-Ibuprofen	0	0	12	0	0	0	0	0	0	0	0	0	0	100
40 % S-Ibuprofen	0	0	0	11	0	0	0	0	0	0	0	0	0	100
5 % S-Ibuprofen	0	0	0	0	12	0	0	0	0	0	0	0	0	100
50 % S-Ibuprofen	0	0	0	0	0	12	0	0	0	0	0	0	0	100
60 % S-Ibuprofen	0	0	0	0	0	0	12	0	0	0	0	0	0	100
70 % S-Ibuprofen	0	0	0	0	0	0	0	12	0	0	0	0	0	100
80 % S-Ibuprofen	0	0	0	0	0	0	0	0	12	0	0	0	0	100
90 % S-Ibuprofen	0	0	0	0	0	0	0	0	0	12	0	0	0	100
95 % S-Ibuprofen	0	0	0	0	0	0	0	0	0	0	12	0	0	100
Pure R-Ibuprofen	0	0	0	0	0	0	0	0	0	0	0	12	0	100
Pure S-ibuprofen	0	0	0	0	0	0	0	0	0	0	0	0	12	100
Total	12	12	12	11	12	12	12	12	12	12	12	12	12	100

Canonical Scores Plot

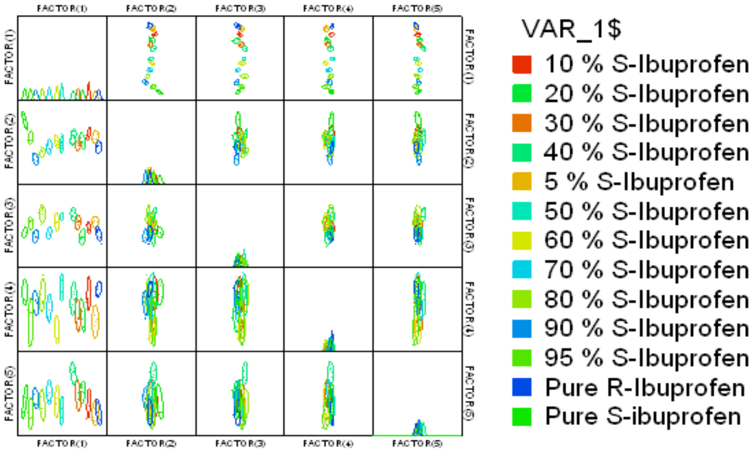

The canonical scores plot of semi-quantitative assay of ibuprofen in the presence of 0 to 10 % increasing concentration of acetate.

Impurity: 0 to 10 % Pyrophosphate

The jackknifed classification matrix of semi-qualitative assay in the presence of 0 to 10 % increasing concentration of pyrophosphate using **S1-S4**.

Jackknifed Classification Matrix	10 % S-Ibuprofen	20 % S-Ibuprofen	30 % S-Ibuprofen	40 % S-Ibuprofen	5 % S-Ibuprofen	50 % S-Ibuprofen	60 % S-Ibuprofen	70 % S-Ibuprofen	80 % S-Ibuprofen	90 % S-Ibuprofen	95 % S-Ibuprofen	Pure R-Ibuprofen	Pure S-ibuprofen	%correct
10 % S-Ibuprofen	12	0	0	0	0	0	0	0	0	0	0	0	0	100
20 % S-Ibuprofen	0	12	0	0	0	0	0	0	0	0	0	0	0	100
30 % S-Ibuprofen	0	0	12	0	0	0	0	0	0	0	0	0	0	100
40 % S-Ibuprofen	0	0	0	12	0	0	0	0	0	0	0	0	0	100
5 % S-Ibuprofen	0	0	0	0	12	0	0	0	0	0	0	0	0	100
50 % S-Ibuprofen	0	0	0	0	0	12	0	0	0	0	0	0	0	100
60 % S-Ibuprofen	0	0	0	0	0	0	12	0	0	0	0	0	0	100
70 % S-Ibuprofen	0	0	0	0	0	0	0	12	0	0	0	0	0	100
80 % S-Ibuprofen	0	0	0	0	0	0	0	0	12	0	0	0	0	100
90 % S-Ibuprofen	0	0	0	0	0	0	0	0	0	12	0	0	0	100
95 % S-Ibuprofen	0	0	0	0	0	0	0	0	0	0	12	0	0	100
Pure R-Ibuprofen	0	0	0	0	0	0	0	0	0	0	0	12	0	100
Pure S-ibuprofen	0	0	0	0	0	0	0	0	0	0	0	0	12	100
Total	12	12	12	12	12	12	12	12	12	12	12	12	12	100

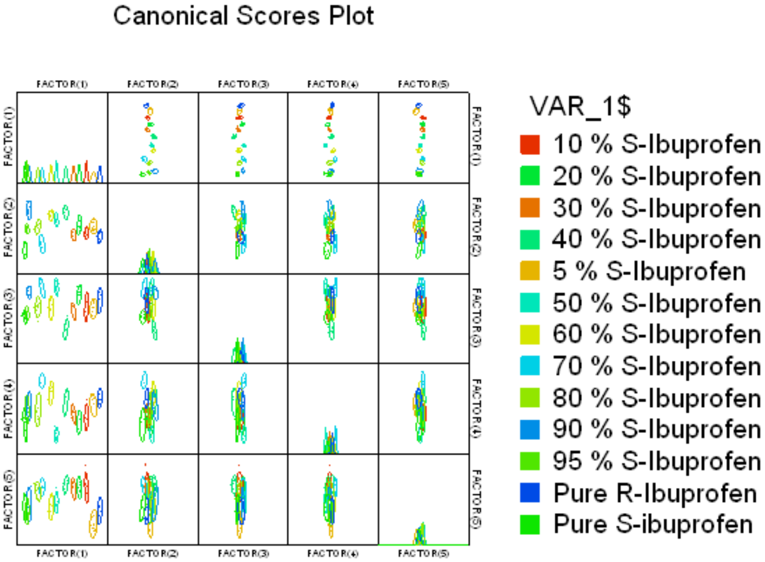

The canonical scores plot of semi-quantitative assay of ibuprofen in the presence of 0 to 10 % increasing concentration of pyrophosphate.

Quantitative analysis of ketoprofen

The jackknifed classification matrix of semi-qualitative assay of ketoprofen using **S1-S4**.

Jackknifed Classification Matrix	10 % S-Ketoprofen	20 % S-Ketoprofen	30 % S-Ketoprofen	40 % S-Ketoprofen	5 % S-Ketoprofen	50 % S-Ketoprofen	60 % S-Ketoprofen	70 % S-Ketoprofen	80 % S-Ketoprofen	90 % S-Ketoprofen	95 % S-Ketoprofen	Pure R-Ketoprofen	Pure S-Ketoprofen	%correct
10 % S-Ketoprofen	12	0	0	0	0	0	0	0	0	0	0	0	0	100
20 % S-Ketoprofen	0	12	0	0	0	0	0	0	0	0	0	0	0	100
30 % S-Ketoprofen	0	0	12	0	0	0	0	0	0	0	0	0	0	100
40 % S-Ketoprofen	0	0	0	12	0	0	0	0	0	0	0	0	0	100
5 % S-Ketoprofen	0	0	0	0	12	0	0	0	0	0	0	0	0	100
50 % S-Ketoprofen	0	0	0	0	0	12	0	0	0	0	0	0	0	100
60 % S-Ketoprofen	0	0	0	0	0	0	12	0	0	0	0	0	0	100
70 % S-Ketoprofen	0	0	0	0	0	0	0	12	0	0	0	0	0	100
80 % S-Ketoprofen	0	0	0	0	0	0	0	0	12	0	0	0	0	100
90 % S-Ketoprofen	0	0	0	0	0	0	0	0	0	12	0	0	0	100
95 % S-Ketoprofen	0	0	0	0	0	0	0	0	0	0	12	0	0	100
Pure R-Ketoprofen	0	0	0	0	0	0	0	0	0	0	0	12	0	100
Pure S-Ketoprofen	0	0	0	0	0	0	0	0	0	0	0	0	12	100
Total	12	12	12	12	12	12	12	12	12	12	12	12	12	100

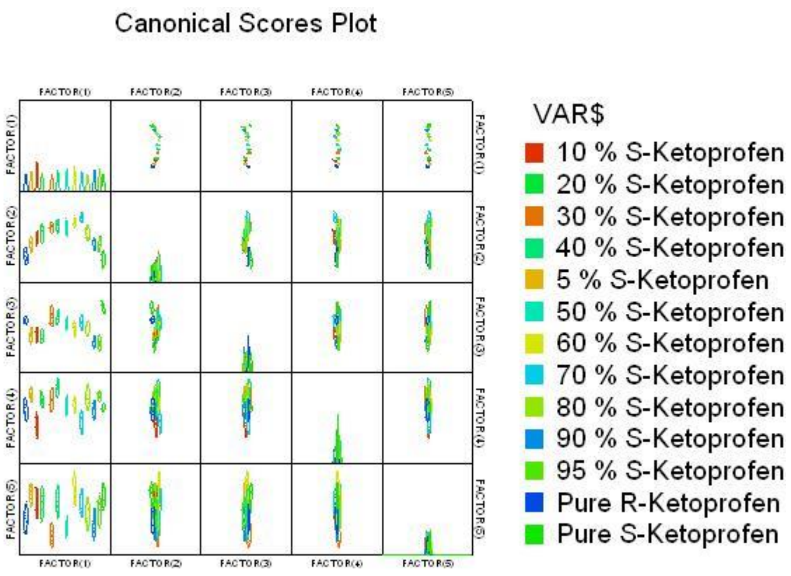

The canonical scores plot of semi-quantitative assay of ketoprofen.

DFT calculations

Computational calculations were performed at the B3LYP/6-31G* level using Spartan'08 (Wavefunction, Inc.). The optimized structure of macrocycle **S2** is shown below.

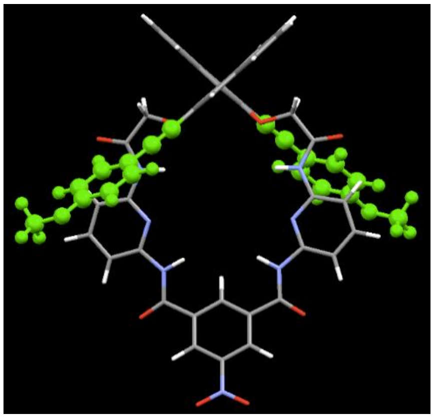

The coordinates for the optimized structure of **S2**.

O 0.0141823 -3.08537 -0.145549
O -3.15485 -4.73098 0.127977
O -6.62712 2.53816 -0.193541
O 2.85923 -0.636566 0.671278
O 5.23912 1.90515 -0.158489
O -1.02648 6.86818 -1.32694
O -7.06529 7.32716 0.471553
O -5.37122 8.6653 0.159367
N -2.56295 -2.50247 0.0329258
N -3.69286 -0.517883 -0.0601322
N -4.53831 1.58837 -0.163926
N 2.93299 1.87532 -0.105796
N 1.27846 3.41327 -0.430639
N -0.564262 4.72118 -0.647126
N -5.88614 7.5505 0.208105
C 2.392 -3.24456 -0.204746
C 3.60104 -3.46982 -0.941698
C 4.88046 -3.48168 -0.32319
C 6.02386 -3.70847 -1.05927
C 5.94867 -3.93471 -2.45395
C 4.72472 -3.94305 -3.08226
C 3.52587 -3.72167 -2.35087
C 2.26145 -3.74996 -2.98287
C 1.08701 -3.56305 -2.26953
C 1.17638 -3.31773 -0.857863
C -0.833037 -4.22291 0.0633297
C -2.31037 -3.84419 0.0706586
C -3.80221 -1.84733 0.0320306
C -5.03488 -2.51118 0.121619

C	-6.18044	-1.72082	0.0966153
C	-6.10015	-0.33331	-0.00964399
C	-4.81412	0.211937	-0.078538
C	-5.40776	2.64957	-0.21921
C	2.42911	-2.90758	1.25344
C	2.1999	-3.90785	2.25433
C	2.00529	-5.27862	1.93347
C	1.78614	-6.21567	2.92078
C	1.74955	-5.83119	4.28199
C	1.94949	-4.51492	4.62858
C	2.1873	-3.52512	3.63599
C	2.42253	-2.1751	3.98426
C	2.67883	-1.20996	3.0218
C	2.67709	-1.60471	1.64164
C	4.18704	-0.118497	0.52203
C	4.17639	1.33099	0.0465938
C	2.5887	3.16591	-0.527597
C	3.51166	4.10195	-1.01783
C	3.00601	5.33662	-1.41616
C	1.64504	5.6256	-1.32485
C	0.826952	4.61159	-0.816915
C	-1.39995	5.78021	-0.908839
C	-5.01756	6.38674	-0.0699141
C	-5.57278	5.11304	-0.0254139
C	-4.75497	4.00974	-0.287479
C	-3.40549	4.2212	-0.595911
C	-2.85397	5.50835	-0.613084
C	-3.67645	6.60953	-0.360673
H	-3.54371	1.76366	-0.126373
H	-0.593898	-4.68675	1.02796
H	-0.69757	-4.97157	-0.721669
H	-0.943182	3.90258	-0.190219
H	4.73785	-0.712838	-0.215233
H	4.73363	-0.146938	1.46921
H	-1.75573	-1.89512	-0.0654517
H	4.94824	-3.32018	0.747864
H	6.99098	-3.7168	-0.564128
H	6.85765	-4.10658	-3.02343
H	4.65305	-4.12454	-4.15186
H	2.20489	-3.92866	-4.05266
H	-5.06774	-3.58726	0.208064
H	-7.1563	-2.19406	0.163562
H	-6.96997	0.306694	-0.0304547
H	2.15047	1.29073	0.169895
H	1.64343	-7.2587	2.65194
H	1.57055	-6.57822	5.05003

H	1.93389	-4.20924	5.67191
H	2.41124	-1.88576	5.03107
H	4.5615	3.85352	-1.07486
H	3.68528	6.09018	-1.80543
H	1.22525	6.5761	-1.62082
H	-6.62137	4.9678	0.201511
H	-2.77883	3.3825	-0.884821
H	-3.27168	7.61324	-0.393537
H	2.04409	-5.58679	0.893297
C	-0.178011	-3.59157	-2.92077
C	-1.25352	-3.64489	-3.48892
C	-2.50476	-3.70528	-4.16657
C	-4.97367	-3.82264	-5.5161
C	-2.61248	-3.29762	-5.51483
C	-3.6603	-4.17426	-3.51626
C	-4.88392	-4.23377	-4.17964
C	-3.82642	-3.35415	-6.17808
H	-1.72956	-2.93283	-6.03066
H	-3.59972	-4.5019	-2.48296
H	-5.75371	-4.60119	-3.64777
H	-3.91763	-3.04134	-7.21343
C	2.93089	0.14244	3.38415
C	3.18893	1.28965	3.7007
C	3.50178	2.62404	4.08555
C	4.13948	5.25383	4.86875
C	3.07681	3.13134	5.33377
C	4.25183	3.46577	3.2443
C	4.56891	4.76693	3.62687
C	3.38968	4.42378	5.71885
H	2.49741	2.49444	5.99518
H	4.60368	3.09622	2.286
H	5.15061	5.38605	2.95378
H	3.0674	4.8194	6.67686
O	-6.11554	-3.83986	-6.2566
O	4.39863	6.50278	5.34217
C	5.15043	7.3922	4.52831
H	6.15094	6.99418	4.31532
H	5.24333	8.31494	5.10338
H	4.63525	7.60386	3.58237
C	-7.31174	-4.29796	-5.64244
H	-7.22109	-5.34117	-5.31299
H	-8.08597	-4.22648	-6.40813
H	-7.58876	-3.67058	-4.78546

References

[1] (a) A. K. Connors *Binding Constants: the Measurement of Molecular Complex Stability*; Wiley: New York, 1987. (b) A. E. Hargrove, Z. Zhong, J. L. Sessler, and E. V. Anslyn, *New J. Chem.*, 2010, **34**, 348.

[2] http://www.originlab.com/.

[3] T. R. Wu, L. Shen, J. M. Chong *Org. Lett*. 2004, **6**, 2701.

[4] T. Ema, D. Tanida, T. Sakai *J. Am. Chem. Soc*. 2007, **129**, 10591.

[5] F. Song, N. Fei, F. Li, S. Zhang, Y. Cheng, and C. Zhu, *Chem. Commun*., 2013, **49**, 2891.
